# AID/APOBEC-network reconstruction identifies pathways associated with survival in ovarian cancer

**DOI:** 10.1186/s12864-016-3001-y

**Published:** 2016-08-16

**Authors:** Martin Svoboda, Anastasia Meshcheryakova, Georg Heinze, Markus Jaritz, Dietmar Pils, Dan Cacsire Castillo-Tong, Gudrun Hager, Theresia Thalhammer, Erika Jensen-Jarolim, Peter Birner, Ioana Braicu, Jalid Sehouli, Sandrina Lambrechts, Ignace Vergote, Sven Mahner, Philip Zimmermann, Robert Zeillinger, Diana Mechtcheriakova

**Affiliations:** 1 Department of Pathophysiology and Allergy Research, Center for Pathophysiology, Infectiology and Immunology, Medical University of Vienna, Vienna, Austria; 2 Section for Clinical Biometrics, Center for Medical Statistics, Informatics, and Intelligent Systems, Medical University Vienna, Vienna, Austria; 3 Research Institute of Molecular Pathology, Vienna Biocenter, Vienna, Austria; 4 Molecular Oncology Group, Department of Obstetrics and Gynecology, Comprehensive Cancer Center, Medical University of Vienna, Vienna, Austria; 5 Comparative Medicine, Messerli Research Institute of the Veterinary University of Vienna, Medical University of Vienna and University of Vienna, Vienna, Austria; 6 Department of Pathology, Medical University of Vienna, Vienna, Austria; 7 Department of Gynecology, European Competence Center for Ovarian Cancer, Campus Virchow Klinikum, Charité - Universitätsmedizin Berlin, Berlin, Germany; 8 Division of Gynecological Oncology, Department of Obstetrics and Gynecology, Universitaire Ziekenhuizen Leuven, Katholieke Universiteit Leuven, Leuven, Belgium; 9 Department of Gynecology and Gynecologic Oncology, University Medical Center Hamburg-Eppendorf, Hamburg, Germany; 10 Nebion AG, Zürich, Switzerland

**Keywords:** The AID/APOBEC family, Multigene signature, Primary serous ovarian carcinoma, Multivariable survival models, Prognostic effect, Integrated analysis of disease-relevant pathways

## Abstract

**Background:**

Building up of pathway-/disease-relevant signatures provides a persuasive tool for understanding the functional relevance of gene alterations and gene network associations in multifactorial human diseases. Ovarian cancer is a highly complex heterogeneous malignancy in respect of tumor anatomy, tumor microenvironment including pro-/antitumor immunity and inflammation; still, it is generally treated as single disease. Thus, further approaches to investigate novel aspects of ovarian cancer pathogenesis aiming to provide a personalized strategy to clinical decision making are of high priority. Herein we assessed the contribution of the *AID/APOBEC* family and their associated genes given the remarkable ability of AID and APOBECs to edit DNA/RNA, and as such, providing tools for genetic and epigenetic alterations potentially leading to reprogramming of tumor cells, stroma and immune cells.

**Results:**

We structured the study by three consecutive analytical modules, which include the multigene-based expression profiling in a cohort of patients with primary serous ovarian cancer using a self-created AID/APOBEC-associated gene signature, building up of multivariable survival models with high predictive accuracy and nomination of top-ranked candidate/target genes according to their prognostic impact, and systems biology-based reconstruction of the AID/APOBEC-driven disease-relevant mechanisms using transcriptomics data from ovarian cancer samples. We demonstrated that inclusion of the AID/APOBEC signature-based variables significantly improves the clinicopathological variables-based survival prognostication allowing significant patient stratification. Furthermore, several of the profiling-derived variables such as *ID3*, *PTPRC/CD45*, *AID*, *APOBEC3G*, and *ID2* exceed the prognostic impact of some clinicopathological variables. We next extended the signature-/modeling-based knowledge by extracting top genes co-regulated with target molecules in ovarian cancer tissues and dissected potential networks/pathways/regulators contributing to pathomechanisms. We thereby revealed that the AID/APOBEC-related network in ovarian cancer is particularly associated with remodeling/fibrotic pathways, altered immune response, and autoimmune disorders with inflammatory background.

**Conclusions:**

The herein study is, to our knowledge, the first one linking expression of entire AID/APOBECs and interacting genes with clinical outcome with respect to survival of cancer patients. Overall, data propose a novel AID/APOBEC-derived survival model for patient risk assessment and reconstitute mapping to molecular pathways. The established study algorithm can be applied further for any biologically relevant signature and any type of diseased tissue.

**Electronic supplementary material:**

The online version of this article (doi:10.1186/s12864-016-3001-y) contains supplementary material, which is available to authorized users.

## Background

Accumulated knowledge on dysregulated cellular checkpoints associated with cancer development and systematic studies using genomic analysis tools have suggested many new classes of cancer-causing and/or cancer-promoting genes. The discovery of AID/APOBEC gene family members with their potential multifaceted contribution to malignant transformation gave a fundamental impact [1, 2]. In humans, the AID/APOBEC family consists of eleven molecules including AID (activation-induced cytidine deaminase, gene name: *AICDA*) and APOBECs (apolipoprotein B mRNA editing enzyme, catalytic polypeptide-like) with the remarkable ability to edit DNA or RNA through cytosine deamination and thus providing tools to introduce DNA or RNA alterations/damages [3–5]. Under physiological conditions, AID is expressed in activated B cells within germinal centers and responsible for the diversification processes of the immunoglobulin genes by triggering both somatic hypermutation and class switch recombination events [6]. Beside genetic modifications it has been shown that AID may also contribute to epigenetic reprogramming by deaminating methylated cytosine [7]; in conjunction with T:G mismatch repair, this leads to DNA demethylation. The APOBEC3 subfamily (containing seven members) has been implicated in the innate immune defense against endogenous transposable genetic elements, endogenous retroviruses as well as exogenous viruses [8–10] based on the ability to induce DNA damage. In contrast to other family members, APOBEC1 was characterized as RNA-editing enzyme by targeting the *ApoB* pre-mRNA [1, 11]; later additional mRNA targets have been described [12].

Given that under pathological circumstances AID and APOBECs’ aberrant expression/activity and/or aberrant mechanisms of recruitment to target(s) and/or aberrant processing of the resulting mismatches might take place, their oncogenicity and contribution to the development and/or progression of cancer have been proposed [2, 13–23]. In B-cell malignancies, AID is responsible for DNA damage leading to double-strand DNA breaks followed by translocation of oncogenes [24–28]. In respect of solid tumors, the importance of AID for oncogenesis was strengthened since it became evident that under pathophysiological circumstances including chronic inflammation the AID expression and activity is not restricted to B cells and Ig locus; AID can also mutate non-Ig genes including among others *TP53* and the *CDKN2b-CDKN2a* locus as targets [20, 28–32]. Among the organs of cancerous or inflamed tissues in which ectopic expression of AID was thus far detected in cells of non-B-cell origin are liver, esophagus, lung, stomach, and colon [20, 29, 31, 33–35]. Beside AID, APOBEC2 was recently identified as risk factor in liver and lung tumorigenesis [19]. Importantly, two independent meta-analyses-based studies identified a link between deleterious somatic mutations with cytosine mutation bias in several cancer types and APOBEC expression/enzymatic activities, with one member of the APOBEC3 subfamily, APOBEC3B, being responsible for the majority of cytosine mutations [13, 36]. It was proposed that for breast cancer APOBEC3B may represent a new marker and target [13, 37].

Here we tested the hypothesis that AID and/or other members of the AID/APOBEC family could be part of mechanism(s) contributing to the pathophysiology of ovarian cancer. The rationale behind is enhanced by additional puzzling evidence. Ovarian cancer shows a high degree of genomic instability; practically all classes of mutations, including point mutations and large genomic deletions and insertions, were demonstrated in high-grade serous ovarian cancer in several genes including *BRCA1*/*2* and mutational inactivation of *TP53* [38]. *AID* mRNA expression was shown to be induced by estrogen in an ovarian cancer cell line *in vitro* [39]. A recent study showed that *APOBEC3B* overexpression in ovarian cancer correlated with elevated levels of transversion mutations [40]; however, the clinical relevance of these findings still needs to be demonstrated including the potential prognostic relevance. Generally, the overview picture covering the mutual interrelation of all family members and their association with the clinical outcome of ovarian cancer patients is not yet available. Further aspect to consider is that the ovarian cancer cells may express several AID/APOBEC family members acting in a patient-specific manner; yet, the tumor-infiltrating immune cells of various subsets may as well express more than one molecule, each contributing to diverse, not-yet-known pathomechanisms. Thus, the systems-level overview is required. Although ovarian cancer is a heterogeneous malignancy, it is generally treated as a single disease with the use of standard chemotherapy with platinum derivatives and taxanes after surgery. The treatment strategies might undergo a substantial transformation based on the promising novel treatment options under clinical trials [41]. While high response rates to the initial regimen are observed, a relapse is seen in most of the patients due to the rapid development of drug resistance contributing to the overall poor survival characterized by a 5 year overall survival rate of < 40 % [42, 43]. Therefore, algorithms to investigate novel aspects of ovarian cancer pathophysiology aiming to identify novel molecules/pathways suitable to be used as prognostic or predictive biomarkers and/or drug targets and, thus, to provide a personalized approach to clinical decision making are of high priority.

**Fig. 1:**
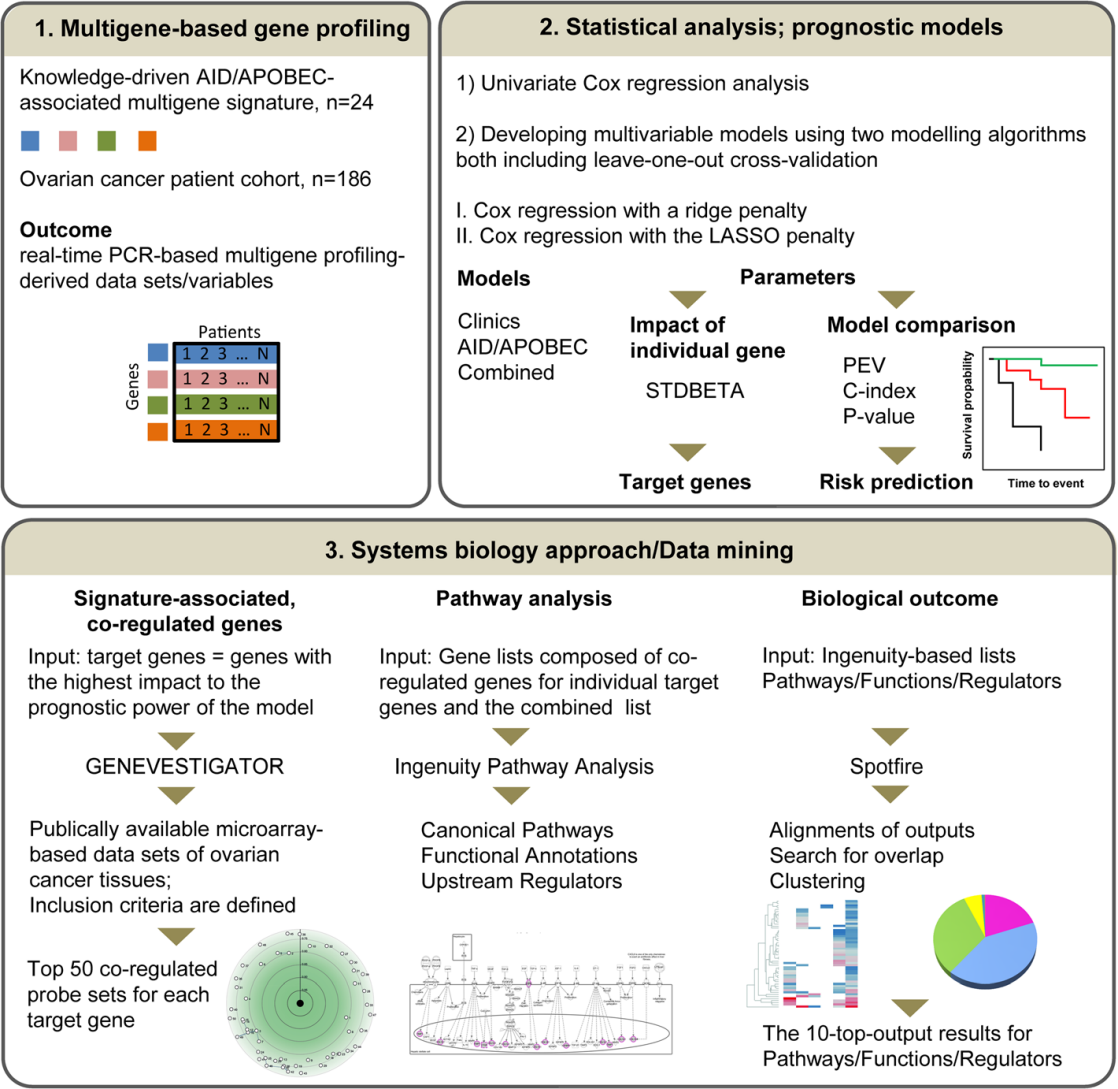
Overview of the study design: from gene expression profiling-based data sets to prognostic models for clinical outcome and biologically meaningful, disease-associated pathways. The proposed algorithm includes three major blocks. (1) The composition of the AID/APOBEC-associated multigene signature (*n* = 24) is assembled based on a knowledge-driven approach and applied for the real-time PCR-based gene expression profiling of a clinically well-characterized patient cohort with primary ovarian carcinoma (*n* = 186). (2) Twenty one profiling-derived variables are correlated with survival data. Univariate Cox regression analysis is applied to assess the prognostic effect of each individual gene and clinical variable. Multivariable Cox regression analysis is applied to build up the survival prognostic models accounting for mutual interconnections between the genes from the signature. Two different multivariable modeling algorithms are used. As outcome, three types of models are created: (i) *Clinics* – the model is based on the clinicopathological parameters only; (ii) *AID/APOBEC* – the model is based on the multigene profiling-derived data sets; and (iii) *Combined* – the model is based on the clinicopathological and gene profiling-derived variables in combination. In both algorithms the standardized coefficients (STDBETA) are used for ranking the individual variables in a model by their importance. The top-ranked genes are defined as target genes for the follow-up analyses. Important to note, parameters such as proportion of explained variation (PEV), c-index and p-value are calculated and used to compare the predictive accuracy and discriminative ability of the individual models. Alignment with patients’ survival data is illustrated by Kaplan-Meier estimates showing patient stratification into low, intermediate, and high risk groups. (3) Systems biology approach is used to assign the defined target genes with prognostic impact to disease-relevant biological pathways. Firstly, the web-based analysis platform for publically available microarray datasets (GENEVESTIGATOR) is used to extract the top genes co-regulated with the target genes in ovarian cancer tissues based on inclusion criteria specified in Methods. Secondly, the obtained gene lists are subjected to the Ingenuity-based core analysis. As input, in addition to the individual lists of co-regulated genes, the combined list (“mixed”) is used to mimic the mutual interconnections within the multigene signature. The core analysis includes alignment with Canonical Pathways, Functional Annotations & Diseases and Upstream Regulators. Thirdly, Spotfire, a data discovery and visualization software, is used for large-scale IPA-derived data processing and data mining. As final outcome, the 10-top Pathways/Functions/Regulators are defined

We and others recently showed (examples in [44–47]) that building up of pathway-/disease-relevant signatures provides a persuasive tool for understanding the functional relevance of gene alterations and gene network associations in human diseases and might be taken as basis for prognostic models assessing patient risk/survival. Evidently, interpretation of a single gene expression pattern under diseased conditions might not be sufficient to understand its role in disease pathogenesis; yet, particular genes composing a multigene signature might be reciprocally interconnected within canonical or not-yet-defined disease-relevant pathway(s). We herein aimed to build up a multigene-based model that is eligible as prognostic for patients with advanced stage of serous ovarian carcinoma and to define novel key AID/APOBEC-associated aspects of ovarian cancer. A comprehensive analysis was applied linking multigene signature-based expression profiling of ovarian cancer specimens with statistical modeling followed by systems biology-based data mining and analysis of disease-relevant biological mechanisms. An overview of the analysis steps is outlined in Fig. 1.

## Methods

### Profile of study patients

Tumor samples of epithelial ovarian cancer (EOC) were collected in the course of the European Commission’s sixth framework program project OVCAD from five European university hospitals (Ovarian Cancer: Diagnosis of a silent killer; grant agreement no. 018698) [48]. Information on clinicopathological characteristics was documented by experienced clinicians. The clinicopathological characteristics of the 186 patients with primary EOC are summarized in Table 1; the patient group is a part of the patient cohort under study of the OVCAD consortium [49, 50]. Patient inclusion criterion comprises the epithelial ovarian cancer with advanced disease (FIGO II – IV); the majority of patients had advanced-stage ovarian cancer (FIGO III and IV, 95 %), G3 tumors (74 %), and the majority of tumors was of serous histology (88 %). 71 % of patients could be optimally cytoreduced with no residual disease after initial surgery; absence of residual disease was defined as macroscopically complete resection of tumor material. All patients received standard adjuvant chemotherapy including platinum-based anti-cancer agents. Eight percent of patients received neoadjuvant chemotherapy. Patients with recurrence or progressive disease until 6 months after the end of chemotherapy were defined as chemotherapy resistant. The median age at diagnosis was 57 years (range, 26 to 85 years); the median follow-up time was 30.0 months (95 % CI: 27.4-32.6). There were 54 cases (29 %) of death related to EOC reported during the follow-up period, designated as events below.

**Table 1:** Clinicopathological characteristics of study patients

Patients (%)	186	(100.0)
Age at diagnosis [years]		
Median (range)	57	(26–85)
Progression-free survival [months]		
Median (range)	16	(1–48)
Number of recurrencies (%)	106	(57.0)
Overall survival [months]		
Median (range)	24	(1–49)
Number of deaths (%)	54	(29.0)
Histology (%)		
Serous	164	(88.2)
Non-serous	22	(11.8)
FIGO (%)		
II	9	(4.8)
III	148	(79.6)
IV	29	(15.6)
Grading (%)		
1	6	(3.2)
2	43	(23.1)
3	137	(73.7)
Residual disease after initial surgery (%)		
None	132	(71.0)
≤ 1 cm	34	(18.3)
> 1 cm	20	(10.8)
Type of chemotherapy (%, 30 missing [16.1 %])		
Adjuvant	124	(66.7)
Neoadjuvant	15	(8.1)
Intraperitoneal	17	(9.1)
Response to first-line chemotherapy (%, 1 missing)		
Responder	138	(74.2)
Non-Responder	47	(25.3)

### Cell lines

The human ovarian carcinoma cell lines A2780 and A2780ADR were obtained from the European Collection of Cell Cultures (Salisbury, Wiltshire, United Kingdom). A2780 is the parent line to the adriamycin resistant A2780ADR. Although adriamycin is not a therapy regimen for ovarian cancer, but considering that A2780 cell model is featured by high chemosensitivity to cisplatin, while A2780ADR cell line exhibits a collateral resistance to cisplatin, both cell lines are often used as *in vitro* models to study the acquisition of drug resistance [51, 52]. The human ovarian carcinoma cell lines OVCAR-3 and SK-OV-3 were obtained from the ATCC (Manassas, VA). The cell lines were maintained in phenol-red-free RPMI-1640 medium supplemented with L-glutamine (PAN-Biotech GmbH, Aidenbach, Germany), 10 % Foetal Calf Serum (FCS) (Invitrogen, Carlsbad, CA) and 1 % penicillin (10,000 U/ml)/streptomycin (10 mg/ml) solution (Invitrogen) in a humidified atmosphere at 37 °C with 5 % CO_2_.

### RNA isolation from tumor tissues and ovarian cancer cell lines

Total RNA from tissues was isolated using the ABI 1600 nucleic acid prepstation (Applied Biosystems, Foster City, CA, USA) following the instructions of the manufacturer as described previously [49]. Total RNA from A2780, A2780ADR, OVCAR-3 and SK-OV-3 cell lines was isolated using the RNeasy Mini kit (Qiagen, Hilden, Germany) including DNase I treatment. The concentration, purity and integrity of RNA samples were determined on a Nanodrop ND-1000 (Kisker-Biotech, Steinfurt, Germany) and agarose gel electrophoresis.

### Real-time PCR analysis

0.5 μg of total RNA from tissue specimens and 1 μg of total RNA from the cell lines was reverse transcribed using the High Capacity cDNA RT kit (Applied Biosystems) according to the instructions of the manufacturer. Given the patient-specific composition of various cell types within ovarian cancer tissues, for accurate normalization of mRNA between ovarian cancer tissue specimens we selected *ACTB*, *TOP1*, *UBC*, and *YWHAZ* out of a panel of 12 housekeeping genes (HKGs) as appropriate reference genes using a geNorm kit (PrimerDesign Ltd., Southampton, UK) and the geNorm software [53]. For the cancer cell lines, *EEF1A1* and *UBC* were used as appropriate reference HKGs as estimated by DataAssist software (Applied Biosystems). Primers for genes of interest composing the AID/APOBEC-associated multigene signature were designed using Primer Express 3.0 software (Applied Biosystems) and validated using a normal tissue panel (Takara, Clontech Laboratories Inc., Mountain View, USA) as previously described [45]. Primer sequences are displayed in Additional file 1: Table S1. The assay for *ACTB* was from Applied Biosystems; assays for the reference HKGs *TOP1*, *UBC*, and *YWHAZ* were purchased from PrimerDesign Ltd; primers for *ESR1* and *ESR2* were purchased from Applied Biosystems (Additional file 1: Table S2).

Real-time PCR analysis was performed on ABI 7900HT instrument equipped with SDS 2.3 software (Applied Biosystems) in the 384-well plate format using POWER SYBR Green Master Mix (Applied Biosystems) or, in case hydrolysis probe assays were used, Gene Expression Master Mix (Applied Biosystems). The qPCR Human Reference Total RNA (Clontech Laboratories Inc.) was assigned as calibrator sample to which gene expression levels of all other samples are compared. Subsequently, raw Ct values were exported into Microsoft Excel and results were calculated using the ΔΔCt method [54] as relative quantities (RQ) normalized to the geometric mean of the four or of the two HKGs specified above for ovarian cancer tissues and cells lines, respectively, and shown relative to the calibrator sample.

The composition of the AID/APOBEC-associated multigene signature used for profiling of the patient cohort and the cell lines is specified in Results.

### Statistical analysis

Profiling-derived values were log2 transformed for Cox regression models to avoid disproportional impact of outliers. Missing values were imputed using the R package mice [55]. Correlation coefficients were calculated by Pearson's correlation for log2 transformed values using SPSS. Hazard ratios and corresponding 95 % confidence intervals were estimated by univariate Cox regression analysis for both the clinicopathological variables and the gene profiling-based variables using the IBM SPSS statistical package (version 20.0; SPSS Inc., an IBM company, Chicago, USA). Regularized multivariable Cox regression was applied to develop prognostic models using two types of regularization as specified below. Calculations were performed with the R (R Foundation for Statistical Computing, Vienna, Austria) package glmnet [56]. In the first approach, Cox regression with a ridge penalty (ridge) was used for estimating multivariable models. In the second approach, models were generated by simultaneous parameter shrinkage and variable selection using the LASSO (L1-norm penalization). Both approaches introduce a penalty to the likelihood function in order to reduce the inflation of variance of the predictions (overfit) caused by a critical ratio of number of outcome events and number of variables. While by the ridge penalty all variables will enter the final model but with severely shrunken regression coefficients, the LASSO penalty selects only some of the variables for the final model, and assigns regression coefficients of 0 to all other variables. The tuning parameter lambda of the ridge and the LASSO penalties were optimized by minimizing the Cox model’s partial deviance in a leave-one-out cross-validation procedure. An additional leave-one-out cross-validation loop was wrapped around the model development process to obtain cross-validated predictors for each patient. Here, the model was re-estimated N times each time omitting one patient in turn, and the cross-validated predictor for that patient was computed as the vector product of the re-estimated regression coefficients and the patient’s corresponding gene expression values. Global p-values for each model were calculated in SPSS using univariate Cox proportional hazards analysis with the corresponding cross-validated predictors of the model as single covariate. Overall survival (OS) and progression-free survival (PFS) were shown by Kaplan-Meier graphs, stratified by quantiles of the cross-validated linear predictors, and accompanied by corresponding log-rank test p-values. Using the cross-validated predictors, we also assessed the discriminative ability of the model by determining the concordance index (c-index) [57] and its proportion of explained variation (PEV) [58]. The c-index is a discrimination measure and describes, as an average measure over all possible pairs of patients, the concordance of survival times and linear predictors derived from the model. The measure is adjusted by inverse probability weighting techniques to accommodate censored survival times. PEV describes the relative gains in predictive accuracy of the survival status at any time point during follow-up when prediction based on covariates replaces unconditional prediction. Absolute values of standardized regression coefficients (STDBETA or ${\widehat{\beta}}_j^{\ast }$,) were used for comparing and ranking the variables by their importance in prediction. The standardized regression coefficient of a variable *X*_*j*_ is the natural logarithm of the hazard ratio between two patients who differ in *X*_*j*_ by 1 SD (ceteris paribus), and can be calculated as ${\widehat{\beta}}_j$SD(*X*_*j*_). Standardized coefficients were then visually compared by depicting *Ŝ*_36_ and ${\widehat{S}}_{36}^{{{}^{\exp}}^{\left({\widehat{\beta}}_j^{\ast}\right)}}$, which are the average 36 months overall survival rate and the estimated 36 months overall survival probability in a subject whose value of *X*_*j*_ differs from the mean of *X*_*j*_ by 1 SD, respectively.

To further estimate the predictive accuracy of the above-described modeling algorithm, we performed the same analyses on the basis of 21 pseudo genes which were obtained by permuting the full block of the original AID/APOBEC-associated 21-gene data set, preserving the distributions and correlation structure within those genes. As outcome, no models could be built for *pseudo AID/APOBEC* using both ridge and LASSO penalties with respect to OS and PFS; when combined with clinicopathological variables, *pseudo AID/APOBEC* did not improve the predictive accuracy and discrimination ability of clinicopathological variables. This provides evidence that the applied modeling algorithm is robust against falsely identifying any relevance of randomly selected gene sets.

Correlation coefficients were calculated by Pearson's correlation for log2 transformed values using SPSS; Bonferroni-Holm method was used as multiple-testing correction. *P*-values ≤ 0.05 were considered as indicating statistical significance.

Group differences were assessed by two-way analysis of variance (ANOVA) and Tukey's post hoc test.

### Expression profiling of signature-associated genes in ovarian cancer cell lines using the published microarray-based data sets

We examined the expression profiles of genes comprising the AID/APOBEC signature across previously published microarray data sets using the GENEVESTIGATOR platform. GENEVESTIGATOR is a manually curated web-based analysis platform for publicly available transcriptomic data sets [59, 60]. For analysis, we selected data sets from the Affymetrix Human Genome U133 Plus 2.0 Array platform; out of a total of 54037 arrays, we selected data attributed to ovarian cancer cell lines applying the filter “Cell Lines_Pathological Cell Lines_Neoplastic Cell Lines_Ovary_All”; this selection included 149 arrays. The expression values (log2 transformed) were exported from GENEVESTIGATOR for follow-up clustering and statistical analyses. Clustering analysis and follow-up graphical representation was performed using Cluster 3.0 and Java TreeView programs.

### Analysis of signature-associated, co-expressed genes using the published microarray-based data sets

For the *in silico* identification of genes showing co-regulation with the top candidate genes ranked within the combined model (ridge) by maximal impact to the prognostic effect (our heuristic solution is to use the cutpoint at STDBETA ≥ |0.15|), we used the GENEVESTIGATOR search engine. The top candidate genes subjected to GENEVESTIGATOR-based analysis were designated in the text below as *target genes*. Members of the APOBEC3 subfamily exhibit high sequence homologies. We checked the specificity of Affymetrix probes covering the APOBEC3 members using BLAST and ensured that the eligible Affymetrix probe set (ID 204205_at) for APOBEC3G is highly specific, whereas the probe set 214995_s_at is cross-reactive with APOBEC3F and the probe set 215579_at does not recognize APOBEC3G. This is in line with previously made conclusions [13]. The specific APOBEC3G probe set was used in GENEVESTIGATOR-based analysis. For the GENEVESTIGATOR-based analysis the following inclusion criteria were applied: (i) we selected data from the Affymetrix Human Genome U133 Plus 2.0 Array platform; (ii) from a total of 709 arrays of EOC, only those with annotated FIGO stage (I-IV) were selected (*n* = 538); (iii) only those target genes were subjected for analysis which showed detectable microarray expression based on the normalized signal intensity in ovarian carcinoma tissues; (iv) analysis was restricted to samples with lowest (10th percentile as threshold) and highest (90th percentile as threshold) target gene expression levels (*n* = 106 selected, Additional file 1: Figure S1); the applied sample selection strategy leads to the exclusion of those genes which show co-expression with the target gene purely based on the non-modulated expression patterns. Additionally, to ensure that changes in target gene expression across the pre-selected tumor samples within both groups are caused by intrinsic gene regulation and not by potential differences in sample quality, a correlation analysis was done between 45 HKGs demonstrating high homogeneity with correlation coefficients > 0.99 (Additional file 1: Figure S2). Next, the lists of the top 50 co-expressed probe sets for each target gene, ranked according to the Pearson correlation coefficient, were exported for further analysis; a combined gene list has been created covering the co-expressed genes of all individual target probe sets (named as *mixed list* below). The content of the mixed list thus reflects the combined input of the multigene signature accounting for/mimicking the mutual interconnections between the individual genes. The Ingenuity Pathway Analysis (IPA) tool was used to assign the co-regulated genes to common biological pathways, biological functions and/or diseases as well as upstream regulating molecules [61]. The IPA Core analysis included the following categories: (i) Canonical Pathways, (ii) Functional Annotations, and (iii) Upstream Regulators. The significance of the association between each gene list and a canonical pathway was measured by right-tailed Fisher’s exact test. As a result, a p-value was obtained, determining the probability that the association between the genes from our data set and a Canonical Pathway/Functional Category/Upstream Regulator can be explained by chance alone. The top ranking was based on the p-value. Only significant outcomes (*p* < 0.05) were taken for follow-up analyses. For alignment of the IPA-derived large-scale data sets, data mining and data visualization the Spotfire software was used [62]. Given the complexity of the follow-up analyses, various approaches were applied. We present herein two algorithms. (I) The top 10 output results (herein designated as the 10-top-output_mixed) were ranked by the corresponding IPA-derived p-values using the mixed list as input data. Subsequently, the position of each 10-top-output_mixed candidate was assessed within the individual target-associated gene list (output_individual). Of particular interest were those which appeared in both the 10-top-output_mixed and at least one of the 10-top-output_individual. (II) The output_mixed results were aligned with the output_individual results searching for the strongest overlap and meaning the mandatory presence in output_mixed and the maximal number of the output_individual (e.g. 5 out of 5 > 4 out of 5 > 3 out of 5; named as output_overlap). Subsequently, the 10-top-output_overlap was ranked by the IPA-derived p-values of the output_mixed results. The unweighted pair group method with arithmetic mean was used as clustering method with Euclidean distance measure and average value as ordering weight.

### Study approval

The study was approved in accordance to the requirements of the ethical committees of the individual institutions participating in OVCAD (EK207/2003, ML2524, HEK190504, EK366, EK260). Informed consent for the scientific use of biological material was obtained from all patients in accordance with the requirements of the ethics committees of the institutions involved; the herein participating OVCAD partners include Department of Gynecology, European Competence Center for Ovarian Cancer at Campus Virchow Klinikum, Charité – Medical University Berlin (Berlin, Germany), Division of Gynecological Oncology, Department of Obstetrics and Gynecology, Universitaire Ziekenhuizen Leuven, Katholieke Universiteit Leuven (Leuven, Belgium), Department of Obstetrics and Gynecology, Medical University of Vienna (Vienna, Austria), and Department of Gynecology, University Medical Center Hamburg - Eppendorf (Hamburg, Germany).

## Results

### A multigene signature approach to assess the patient-specific transcriptional profiles in the context of the clinical relevance

The selection of genes composing the multigene signature is knowledge- and biology-driven. Our expert-designed gene signature is thereby influenced by previous published work and disease relevance, and in this sense is biased towards previous knowledge; importantly, this selection is not based on pre-tested prognostic impact in our study cohort and thereby does not lead to a real bias. This approach represents relatively new way of addressing the pathophysiological relevance of transcriptional profiles and methodologically has indisputable advantage of the real-time PCR-based analysis that ultimately provides results which do not need further methodological validation. It is an appropriate strategy of choice for low-level expressed genes and for genes with high sequence similarity which is truly relevant for AID and APOBEC3 subfamily, respectively. Furthermore, considering the complex cellular composition of ovarian cancer tissue and the current limited knowledge linking the cell type-specific expression patterns of AID and APOBECs with their functionality under diseased conditions, we included all members of the AID/APOBEC family, regardless of other potential ways of their regulation besides those on the transcriptional levels.

The applied gene signature includes the entire *AID/APOBEC* family consisting of *AID (AICDA), APOBEC1, APOBEC2, APOBEC3A, APOBEC3B, APOBEC3C, APOBEC3D, APOBEC3F, APOBEC3G*, *APOBEC3H, and APOBEC4; PTPRC* (also known as *CD45*), *PAX5 (*also known as *BSAP), CD23, NUGGC* (also known as *SLIP-GC*), and *PRDM1* (also known as *BLIMP1*), *ID2* and *ID3* were added accounting for ovarian cancer tissue infiltrating immune cells, B-cell biology and transcriptional control of AID, respectively [17, 63, 64]; the estrogen receptors *ESR1* and *ESR2* were included given the hormone-dependent nature of the analyzed tumor type and the potential involvement of estrogen in *AID* regulation [39]; *DPPA3* (also known as *STELLA*) and *NANOG* are pluripotency-associated genes whose expression was shown to be linked to the AID functional activity [7, 65]; *XRCC5* (also known as *KU80*) and *XRCC6* (also known as *KU70*) are involved in DNA repair mechanism downstream of AID [66]. In sum, the gene panel includes B-cell identity markers, AID/APOBEC family members, genes involved in their regulation, and their functional co-factors or target genes (*n* = 24). Gene names, Gene ID, short functional description from NCBI, synonyms and accession numbers are provided in Additional file 1: Table S3.

### Univariate associations of the individual gene expression-derived variables and the clinicopathological parameters with overall and progression free survival

To determine the clinical relevance of the gene expression data sets of each individual gene of the signature and of the clinicopathological parameters, we first used the classical Cox regression analysis strategy whereby the values were aligned with OS and PFS. Two genes showed statistically significant associations with OS, namely *AID* (HR = 1.18, 95 % CI: 1.04–1.33, *p* = 0.008) and *ID3* (HR = 1.36, 95 % CI: 1.12–1.64, *p* = 0.002), as estimated by univariate Cox regression analysis and summarized in Additional file 1: Table S4. Among clinical risk factors, five variables were associated with OS, namely age (HR = 1.03, 95 % CI: 1.01–1.06, *p* = 0.011), FIGO stage (HR = 1.83, 95 % CI: 1.02–3.29, *p* = 0.044), grading (HR = 2.10, 95 % CI: 1.03–4.31, *p* = 0.042), peritoneal carcinomatosis (HR = 4.17, 95 % CI: 1.78–9.78, *p* = 0.001), and residual disease (HR = 1.98, 95 % CI: 1.15–3.44, *p* = 0.014; Additional file 1: Table S4). With respect to PFS, no significant associations with the gene expression-derived variables were observed (Additional file 1: Table S4). Three clinical variables showed a strong association with PFS: FIGO stage (HR = 2.33, 95 % CI: 1.53–3.54, *p* < 0.001), peritoneal carcinomatosis (HR = 3.88, 95 % CI: 2.27–6.61, *p* < 0.001) and residual disease (HR = 1.95, 95 % CI: 1.29–2.94, *p* = 0.002). *APOBEC1*, *APOBEC2* and *DPPA3* mRNAs were not expressed or expressed at the detection limit in the ovarian cancer tissues of the herein investigated cohort of patients and therefore those variables were excluded from the univariate Cox regression and all subsequent analyses. Thus, the final number of the gene profiling-derived variables included to the follow-up analyses was equal to *n* = 21.

Of note, the follow-up statistical models using Cox regression with ridge and LASSO penalties are not based on the pre-selection of variables according to their significance estimated by univariate Cox regression analysis.

We further performed a correlation analysis for profiling-derived variables. As summarized in Additional file 1: Table S5 a strong correlation (*r* > 0.6; *p* < 0.001) was found between *PTPRC/CD45* and *APOBEC3* family members such as *A3C*, *A3D*, *A3G*, and *A3H* as well as *NUGGC* and *PRDM1*; between *APOBEC3D* and both *APOBEC3G* and *APOBEC3H*, *XRCC6*/*Ku70*, *NUGGC*, and *PRDM1*; between *ID2* and *ID3*; between *XRCC6*/*Ku70* and *XRCC5*/*Ku80* as well as *APOBEC3D*, *ID2* and *ID3*; between *PAX5* and *FCER2*; between *NANOG* and *XRCC6*/*Ku70* and *XRCC5*/*Ku80*; between *PRDM1* and *ID3*, *XRCC6*/*Ku70*, *XRCC5*/*Ku80*, and *NUGGC*. In respect of *AID*, the strongest correlation was found with *PTPRC*/*CD45* (*r* = 0.521; *p* < 0.001). *ESR1* and *ESR2* did not show significant correlation with any other gene of the multigene signature.

### Prognostic models for OS and PFS

**Fig. 2:**
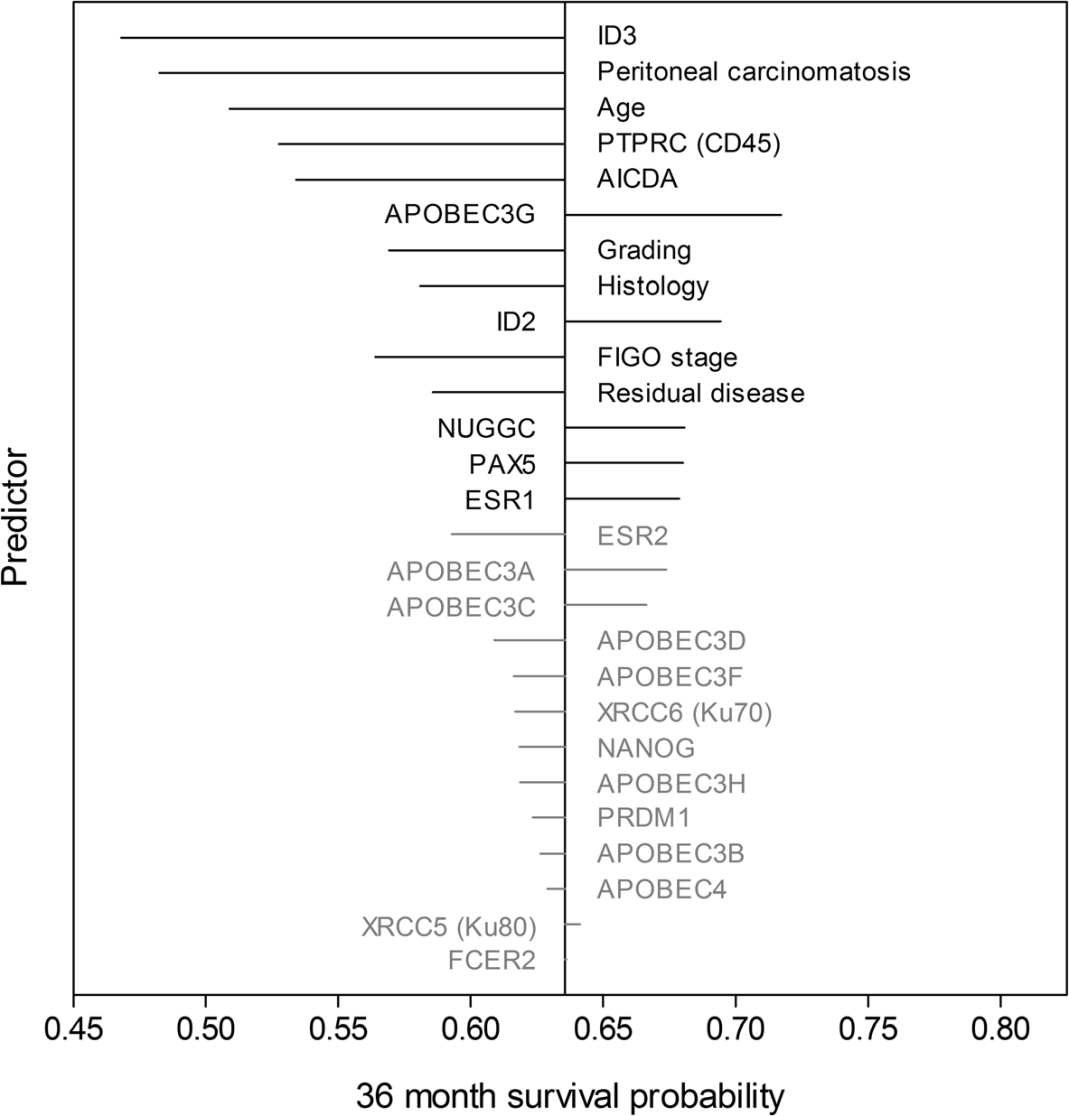
Impact of individual variables on survival prediction of the multivariable model (ridge) for OS if predictors are changed by +1 SD. Survival probabilities are estimated at 36 months of follow-up time. The length of the lines is proportional to the change in prediction in case the value of the indicated variable changes by +1 SD (*left*: negative effect; *right*: positive effect). Variables are ranked according to the absolute changes in prediction which also corresponds to the order within the combined model (Table 3). Gene profiling variables not used for follow-up analyses are displayed in *grey* color

Using Cox regression with ridge and LASSO penalties, we developed multivariable models for evaluating patient prognosis and for stratifying patients into risk groups. Calculations were done for three sets of explanatory variables: (i) using the six clinicopathological variables (*Clinics*), (ii) using the gene profiling-derived AID/APOBEC variables (*AID/APOBEC*), and (iii) combining clinical and multigene-derived variables (*Combined*). The results for the multivariable ridge models are summarized in Tables 2 and 3 and Additional file 1: Table S6. The clinicopathological variables-based model predicts both OS (PEV = 8.8 %, c-index = 0.69, *p* < 0.001) and PFS (PEV = 17.0 %, c-index = 0.68, *p* < 0.001). The AID/APOBEC model showed moderate predictive accuracy and discrimination for OS (PEV = 2.5 %, c-index = 0.59, *p* = 0.025). The combined model had the highest predictive accuracy with respect to OS (PEV = 11.1 %, c-index = 0.7, *p* < 0.001). According to their standardized regression coefficients, the following five genes proved most important for prediction within the AID/APOBEC model: *ID3*, *AID*, *APOBE3G*, *PTPRC/CD45*, and *ESR1* (Table 3). Among the covariates within the combined model, *ID3* emerged as the prognostically most important variable for OS, exceeding the clinical risk factors such as peritoneal carcinomatosis or age. Together with *ID3*, *PTPRC/CD45*, *AID*, and *APOBEC3G* showed high importance for survival prediction standing ahead of the clinical risk factors such as grading and histology (Table 3, Fig. 2).

**Table 2:** Comparative analysis of multivariable models (ridge) for prognostication of OS and PFS

	OS			PFS		
	PEV %	c-index	*P*	PEV %	c-index	*P*
Clinics	8.77	0.69	<0.001	17.0	0.68	<0.001
AID/APOBEC	2.53	0.59	0.025	n.a.	n.a.	n.a.
Combined	11.13	0.70	<0.001; 0.021*	13.6	0.65	<0.001; 0.320*

Cross-validated performance assessment of Cox regression models (ridge) by proportion of explained variation (PEV), concordance index (c-index) and p-value. Models were developed using (i) clinicopathological variables designated as Clinics; (ii) AID/APOBEC multigene-based variables designated as AID/APOBEC; (iii) their combination designated as Combined

* *p*-value for added value of AID/APOBEC on top of Clinics in bivariable models with cross-validated predictors

**Table 3:** Relative importance of individual variables in multivariable models (ridge) for OS

	Clinics					Combined			
Pos.	Variables	beta	HR	STDBETA	Pos.	Variables	beta	HR	STDBETA
1.	Peritoneal carcinomatosis	0.83	2.30	0.38	1.	*ID3*	0.30	1.35	0.52
2.	Age	0.02	1.02	0.27	2.	Peritoneal carcinomatosis	1.05	2.86	0.48
3.	Histology	0.58	1.79	0.19	3.	Age	0.03	1.03	0.40
4.	FIGO stage	0.48	1.62	0.18	4.	*PTPRC (CD45)*	0.19	1.21	0.34
5.	Residual disease	0.37	1.45	0.17	5.	*AICDA*	0.14	1.15	0.33
6.	Grading	0.34	1.41	0.15	6.	*APOBEC3G*	−0.19	0.83	−0.31
	AID/APOBEC				7.	Grading	0.53	1.70	0.23
Pos.	Variables	beta	HR	STDBETA	8.	Histology	0.67	1.96	0.22
1.	*ID3*	0.18	1.19	0.30	9.	*ID2*	−0.12	0.89	−0.22
2.	*AICDA*	0.09	1.09	0.21	10.	FIGO stage	0.50	1.64	0.18
3.	*APOBEC3G*	−0.11	0.89	−0.19	11.	Residual disease	0.36	1.44	0.17
4.	*PTPRC (CD45)*	0.08	1.08	0.14	12.	*NUGGC*	−0.08	0.92	−0.16
5.	*ESR1*	−0.06	0.94	−0.11	13.	*PAX5*	−0.06	0.94	−0.16
6.	*PRDM1*	0.06	1.06	0.10	14.	*ESR1*	−0.08	0.92	−0.16
7.	*APOBEC3B*	0.05	1.05	0.09	15.	*ESR2*	0.04	1.04	0.14
8.	*XRCC5 (Ku80)*	−0.06	0.95	−0.07	16.	*APOBEC3A*	−0.07	0.93	−0.14
9.	*APOBEC3A*	−0.03	0.97	−0.07	17.	*APOBEC3C*	−0.10	0.91	−0.11
10.	*APOBEC3C*	−0.06	0.95	−0.06	18.	*APOBEC3D*	0.06	1.06	0.09
11.	*PAX5*	−0.02	0.98	−0.06	19.	*APOBEC3F*	0.06	1.06	0.07
12.	*ID2*	−0.03	0.97	−0.05	20.	*XRCC6 (Ku70)*	0.05	1.05	0.06
13.	*XRCC6 (Ku70)*	0.04	1.04	0.05	21.	*NANOG*	0.04	1.04	0.06
14.	*FCER2*	−0.02	0.98	−0.05	22.	*APOBEC3H*	0.03	1.03	0.06
15.	*APOBEC3F*	0.03	1.03	0.03	23.	*PRDM1*	0.03	1.03	0.04
16.	*ESR2*	0.01	1.01	0.03	24.	*APOBEC3B*	0.02	1.02	0.03
17.	*APOBEC3H*	−0.01	0.99	−0.01	25.	*APOBEC4*	0.01	1.01	0.02
18.	*APOBEC4*	0.00	1.00	0.01	26.	*XRCC5 (Ku80)*	−0.02	0.98	−0.02
19.	*APOBEC3D*	0.00	1.00	−0.01	27.	*FCER2*	0.00	1.00	0.00
20.	*NUGGC*	0.00	1.00	−0.01					
21.	*NANOG*	0.00	1.00	−0.01					

Within each model, variables are ranked by descending importance as expressed by their absolute standardized regression coefficients. Histology was encoded as “0” for serous and “1” for non-serous; Peritoneal carcinomatosis was encoded as “0” for no and “1” for yes; Grading was encoded as “0” for Grade 1 and 2 and “1” for Grade 3; Residual disease was encoded as “0” for no and “1” for yes; beta, regression coefficient (log hazard ratio); HR, hazard ratio; STDBETA, standardized regression coefficients. By multivariate modeling with penalized likelihood, the multivariate-adjusted HR shows the direction and magnitude of prognostic effect if adjusted for other variables. Ridge regression shifts the HR towards the value of 1.0 to avoid overestimation bias and to decrease variance in models with many variables

**Fig. 3:**
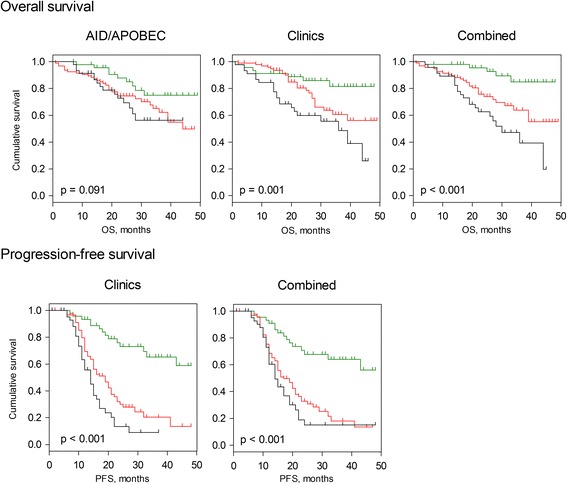
Kaplan-Meier estimates for patient stratification based on the *AID/APOBEC* model, the clinical model, and the combined one (ridge-based). Kaplan-Meier curves for OS and PFS are shown giving patients’ stratification into low risk (*n* = 46, *green*), intermediate risk (*n* = 94, *red*), and high risk (*n* = 46, *black*) groups with the 25th and 75th percentiles serving as thresholds (lower than the 25th percentile indicates low risk). No stable and well calibrated model for *AID/APOBEC* was found in respect of PFS, thus only models for *Clinics* and *Combined* are shown. P-value of the log-rank test is indicated

Patients were sub-divided into low, intermediate, and high risk groups according to their cross-validated predictors for OS based on the ridge model. Figure 3 shows the corresponding Kaplan-Meier graphs. The combined model showed statistically significant differences between the risk groups (log-rank test: *p* < 0.001) giving major improvement of patient stratification. With respect to PFS, the combination of gene expression data sets with the clinical variables did not result in improved patient stratification in comparison to the clinicopathological variables-based model (Fig. 3). Importantly, the results of the second regularization method (LASSO) were in line with those described above and indicated similar predictive abilities with respect to OS by the clinicopathological variables-based model (PEV = 7.54 %, c-index = 0.67, *p* < 0.001), by the AID/APOBEC multigene-based model (PEV = 5.76 %, c-index = 0.64, *p* < 0.001), and superior performance of the combined model (PEV = 10.80 %, c-index = 0.70, *p* < 0.001) (Additional file 1: Tables S7-S9, Additional file 1: Figure S3). The corresponding Kaplan-Meier graphs for OS and PFS using the LASSO penalization are shown in Additional file 1: Figure S4.

### Expression of genes from the AID/APOBEC multigene signature in ovarian cancer cell lines

Given the complex cellular composition of the ovarian cancer tissues we assessed the mRNA expression of genes composing the AID/APOBEC multigene signature by real-time PCR in four ovarian cancer cell lines such as A2780, A2780ADR, OVCAR-3, and SK-OV-3. Expression profiling revealed that the majority of genes were found to be expressed in at least one of the four examined cell lines (Additional file 1: Figure S5); of note, among those are the top genes, which showed the highest impact to the prognostic power of the multivariable model.

We next expanded the scope of expression analysis to a wider range of ovarian cancer cell lines (*n* = 55) across previously published microarray data sets using the GENEVESTIGATOR platform (Additional file 1: Figure S6). The analyzed set of cell lines included, among others, the ovarian cancer cell lines, which had been previously ranked and sub-grouped by their suitability as high-grade serous ovarian cancer tumors on the basis of genomic profiling [67]. With the exception of genes exhibiting low expression and/or expression at the microarray detection limit (*AICDA*, *APOBEC1*, *APOBEC3A*, *PAX5*, and *PTPRC*), the genes composing the AID/APOBEC multigene signature were expressed in various cell lines representing the above mentioned sub-groups. We next applied a hierarchical cluster analysis on the basis of expression values of the signature genes across the analyzed cell lines. The resulting two main clusters for ovarian cancer cell lines accentuate the expression differences for *APOBEC3B*, *APOBEC3C*, and *APOBEC3G* as well as of *ID2* and *ID3* (Additional file 1: Figure S7).

### Systems biology approach linking the gene expression data sets with the disease-relevant biological pathways and functions

To define the AID/APOBEC-attributed biological pathways potentially associated with the pathogenesis of ovarian cancer, a systems biology approach was applied (described in detail in Fig. 1 of study design and in Methods). The eligible genes from the profiling-derived candidate genes with the highest impact to the combined prognostic model include *AID*, *APOBEC3G, ESR1, ID2, ID3, NUGGC, PAX5, and PTPRC/CD45*. However, 3 genes were excluded such as *NUGGC*, since no corresponding probesets do exist on the U133 Plus 2.0 Array, and *AID* and *PAX5* due to the low mRNA expression levels in ovarian cancer tissue when detected by microarray. Thus, *APOBEC3G*, *ESR1*, *ID2*, *ID3*, and *PTPRC/CD45* were used as target genes for follow-up analyses. Next, for each target gene we assessed the co-expressed genes in ovarian cancer tissues by GENEVESTIGATOR. The exported gene lists are summarized in Additional file 1: Tables S10-S14. To assign the co-regulated genes to common biological pathways, biological functions and/or diseases as well as upstream regulating molecules the Ingenuity Pathway Analysis (IPA) tool was used.

### Data-driven signature-associated Canonical pathways

Results of the follow-up IPA-based core analysis in respect of the Canonical Pathways are listed in Table 4 (based on the algorithm I, where the priority is given to the outcome_mixed as specified in Methods). The Canonical Pathway designated as Hepatic Fibrosis/Hepatic Stellate Cell Activation was ranked to position 1. Of note, 8 out of the top 10 canonical pathways derived from the output_mixed were found in at least one output_individual within the 10-top positions (with p-values all < 0.05; Table 4). The strongest overlap between the 10-top-output pathways of mixed and individual was observed for *APOBEC3G* (5/10) and *PTPRC/CD45* (5/10) indicating the strongest contribution from those two genes; in contrast, no overlap was found for *ESR1*. Similarly to the algorithm I-derived results, Hepatic Fibrosis/Hepatic Stellate Cell Activation pathway was as well ranked to position 1 when the algorithm II was applied, where the priority is given to the overlap between the output_mixed and output_individual (Additional file 1: Table S15). The results of overall alignment illustrated by the heat map (Additional file 1: Figure S8, A) and by pie chart (Additional file 1: Figure S9, A) indicate that the Canonical Pathways attributed to the individual target genes are mostly diverse among each other but show a strong overlap with the mixed-attributed Canonical Pathways. To align the results of herein pathway analysis with the related studies, we additionally included the references to the published studies in the area of ovarian carcinoma and others (Table 4 and Additional file 1: Table S15).

**Table 4:** The 10-top-AID/APOBEC signature-linked Canonical Pathways identified by systems biology approach (algorithm I)

Canonical 10-top-output_mixed^a^				Canonical output_individual^b^										Related studies^c^	
				APOBEC3G		ESR1		ID2		ID3		PTPRC (CD45)			
Pos.	Canonical pathways	*P*-value	Molecules	Pos.	Molecules	Pos.	Molecules	Pos.	Molecules	Pos.	Molecules	Pos.	Molecules	Ovarian Cancer	Others
1	Hepatic Fibrosis / Hepatic Stellate Cell Activation	8.32E-08	COL1A1, IGFBP4, CCR5, FN1, CTGF, TIMP1, ACTA2, IL10RA, CCL5, FAS, EGFR					**1**	**COL1A1, IGFBP4, CTGF, TIMP1, ACTA2, FAS, EGFR**	**5**	**IGFBP4, FN1**	*14*	*CCR5, IL10RA, CCL5*	[79, 80, 98]	[81]
2	Altered T Cell and B Cell Signaling in Rheumatoid Arthritis	1.51E-06	HLA-DOA, IL15, TLR8, FCER1G, CD86, TLR3, TNFSF13B, FAS	**3**	**HLA-DOA, IL15, TLR3, TNFSF13B**							**6**	**TLR8, FCER1G, CD86**		[83]
3	Antigen Presentation Pathway	2.40E-05	PSMB9, HLA-DOA, CIITA, HLA-DPB1, HLA-DPA1	**1**	**PSMB9, HLA-DOA, CIITA, HLA-DPB1, HLA-DPA1**									[82, 99]	
4	Communication between Innate and Adaptive Immune Cells	2.45E-05	IL15, TLR8, FCER1G, CD86, CCL5, TLR3, TNFSF13B	**4**	**IL15, CCL5, TLR3, TNFSF13B**							**1**	**TLR8, FCER1G, CD86, CCL5**	[100]	
5	Role of Pattern Recognition Receptors in Recognition of Bacteria and Viruses	3.47E-05	IFIH1, CLEC7A, TLR8, CASP1, CCL5, TLR3, C3AR1	**2**	**IFIH1, CLEC7A, CASP1, CCL5, TLR3**							**9**	**TLR8, CCL5, C3AR1**		[101–103]
6	Allograft Rejection Signaling	1.35E-04	HLA-DOA, FCER1G, CD86, HLA-DPB1, HLA-DPA1, FAS	**6**	**HLA-DOA, HLA-DPB1, HLA-DPA1**							*25*	*FCER1G, CD86*		[104]
7	Crosstalk between Dendritic Cells and Natural Killer Cells	2.09E-04	CSF2RB, ACTA2, IL15, CD86, TLR3, FAS	*21*	*IL15, TLR3*			*40*	*ACTA2,FAS*			*27*	*CSF2RB, CD86*		[105, 106]
8	CCR5 Signaling in Macrophages	5.25E-04	CCR5, FCER1G, CCL5, GNG12, FAS					*27*	*GNG12, FAS*			**4**	**CCR5, FCER1G, CCL5**	[107, 108]	[109]
9	CD28 Signaling in T Helper Cells	8.13E-04	PTPRC, HLA-DOA, FCER1G, CD86, ITPR1, LCP2									**2**	**PTPRC, FCER1G, CD86, LCP2**		[110, 111]
10	Graft-versus-Host Disease Signaling	9.12E-04	HLA-DOA, FCER1G, CD86, FAS									*16*	*FCER1G, CD86*		[112]

^a^IPA nomenclature is used for canonical pathways. The 10-top-output_mixed results are shown; the ranking is based on the corresponding IPA-based *p*-value; the molecules mapped to the pathway are listed

^b^Position of the canonical pathway within the output_individual for each target gene, the corresponding *p*-value and the molecules mapped to the pathway are indicated. **Bold**, within the 10-top-output_individual; *Italic*, outside the 10-top-output_individual. Empty cell: the corresponding pathway was not significant in output_individual

^c^Literature search-based results annotating those canonical pathways in ovarian cancer or other related studies

### Data-driven signature-associated functional annotations and/or diseases

IPA was further used to align the output gene lists with the biological functions and/or diseases named as Functional Annotations. Due to affiliation of functional annotations into several functional categories, the broader IPA classification system, only algorithm II was applied. Results of the 10-top are shown in Tables 5, 6 and 7 and include the results of the output_mixed overlaid with 5, 4, or 3 output_individual. We observed the strong overlap between mixed and *ID2* > *PTPRC/CD45* > *APOBEC3G* > *ID3* and the minor overlap with *ESR1*. Within the top outcomes, the over-representation of the basic cellular functions with the accents to the cell movement linked to the immune system and inflammation-driven diseases was documented. Furthermore, at this categorical level the cancer-related processes appeared (metastasis, triple-negative breast cancer). Additionally, the total alignment patterns are illustrated by heat map (Additional file 1: Figure S8, B) and by pie chart (Additional file 1: Figure S9, B).

**Table 5:** The top-AID/APOBEC signature-linked Functional Annotations identified by systems biology approach (algorithm II; overlap with 5 individual target genes)

Functional Annotation top-output_mixed_and_5_individual								
Pos.	Functional Annotations	Mixed		APOBEC3G	ESR1	ID2	ID3	PTPRC (CD45)
		*P*-value	Molecules	Molecules	Molecules	Molecules	Molecules	Molecules
1	proliferation of cells	1.16E-16	AEBP1, AIF1, ALOX5, AR, ARMC10, BGN, C3AR1, CAMK2N1, CASP1, CCL5, CCR5, CD2, CD47, CD48, CD84, CD86, CDKN1A, CIITA, CLEC7A, COL1A1, COL6A1, COL6A2, CSF2RB, CTGF, CTSS, CYBB, DKK3, DLC1, DOCK2, EGFR, EMILIN1, ENG, EPS8, ESR1, FAS, FBN1, FCER1G, FN1, FOXP1, FYB, GBP2, HCK, HEXB, HLA-DPB1, ID1, ID2, ID3, IGFBP4, IGFBP7, IL10RA, IL15, IRF1, ITGAM, ITPR1, LAIR1, LAMB1, LCP2, LIMA1, LPAR3, LRIG1, LST1, MEIS1, MNDA, MTUS1, MUC16, NID2, NPAS3, NPDC1, PMEPA1, PMP22, PPAP2A, PRRX1, PSMB10, PTPRC, RARRES3, RHBDF1, RHOB, ROCK2, SAMSN1, SASH3, SDC2, SNAI2, SOX17, SPOCK1, SULF2, TIMP1, TLR3, TNFRSF14, TNFSF13B, VCAN, ZFP36	ALOX5, CASP1, CCL5, CIITA, CLEC7A, CTSS, GBP2, HLA-DPB1, IL15, IRF1, ITGAM, PSMB10, RARRES3, TLR3, TNFRSF14, TNFSF13B	AR, ARMC10, CD47, ESR1, LPAR3, LRIG1, MEIS1, MUC16, NPAS3, SOX17	CAMK2N1, CDKN1A, COL1A1, COL6A1, COL6A2, CTGF, DLC1, EGFR, ENG, EPS8, FAS, FOXP1, HEXB, ID1, ID2, ID3, IGFBP4, IGFBP7, LIMA1, MTUS1, NPDC1, PPAP2A, RHBDF1, RHOB, SDC2, SNAI2, SULF2, TIMP1, ZFP36	AEBP1, COL6A2, DKK3, DLC1, EMILIN1, FBN1, FN1, ID1, ID2, ID3, IGFBP4, ITPR1, LAMB1, LIMA1, PMEPA1, PMP22, PPAP2A, PRRX1, RHOB, SDC2, SNAI2, SULF2, VCAN	AIF1, C3AR1, CCL5, CCR5, CD2, CD48, CD84, CD86, CSF2RB, CYBB, DOCK2, FCER1G, FYB, HCK, IL10RA, LAIR1, LCP2, LST1, MNDA, PTPRC, SAMSN1, SASH3

IPA nomenclature was used for Functional Annotations. The top-output_mixed_and_5 individual results are shown; the ranking is based on the corresponding IPA-based p-value of the output_mixed; the molecules mapped to the Functional Annotation are listed

**Table 6:** The top-AID/APOBEC signature-linked Functional Annotations identified by systems biology approach (algorithm II; overlap with 4 individual target genes)

Functional Annotations-top-output_mixed_and_4_individual								
Pos.	Functional Annotations	Mixed		APOBEC3G	ESR1	ID2	ID3	PTPRC (CD45)
		*P*-value	Molecules	Molecules	Molecules	Molecules	Molecules	Molecules
1	arthritis	1.53E-17	AIF1, ALOX5, AR, BGN, C3AR1, CASP1, CCL5, CCR5, CD86, CDH11, CDKN1A, CIITA, COL6A1, CSF2RB, CTSS, FAS, FCER1G, FCGRT, FN1, FSTL1, GBP2, GZMA, HCK, HLA-DOA, HLA-DPA1, HLA-DPB1, IGFBP4, IGFBP7, IL10RA, IL15, IRF1, ITGAM, ITPR1, JAM3, LST1, MS4A6A, PSMB9, PTPRC, SAMD9L, SDC2, SNAI2, SPOCK1, TAGAP, TIMP1, TLR3, TNFSF13B, ZFP36	ALOX5, CASP1, CCL5, CIITA, CTSS, GBP2, HLA-DOA, HLA-DPA1, HLA-DPB1, IL15, IRF1, ITGAM, MS4A6A, PSMB9, SAMD9L, TAGAP, TLR3, TNFSF13B		CDH11, CDKN1A, COL6A1, FAS, FCGRT, IGFBP4, IGFBP7, SDC2, SNAI2, TIMP1, ZFP36	BGN, CDH11, FN1, IGFBP4, ITPR1, JAM3, SDC2, SNAI2, SPOCK1	AIF1, C3AR1, CCL5, CCR5, CD86, CSF2RB, FCER1G, GZMA, HCK, IL10RA, LST1, MS4A6A, PTPRC
2	cell death of immune cells	8.52E-15	CASP1, CCL5, CCR5, CD2, CD47, CD86, CDKN1A, CIITA, COL1A1, CSF2RB, CYBB, EGFR, ESR1, EYA2, FAS, FCER1G, FN1, GIMAP4, GZMA, HCK, ID2, ID3, IFIH1, IL15, IRF1, ITGAM, ITPR1, LAIR1, LRIG1, MEIS1, PTPRC, SNAI2, TIMP1, TLR3, TNFSF13B	CASP1, CCL5, CIITA, IFIH1, IL15, IRF1, ITGAM, TLR3, TNFSF13B	CD47, ESR1, EYA2, LRIG1, MEIS1	CDKN1A, COL1A1, EGFR, FAS, ID2, ID3, SNAI2		CCL5, CCR5, CD2, CD86, CSF2RB, CYBB, FCER1G, GIMAP4, GZMA, LAIR1, PTPRC
3	rheumatoid arthritis	2.2E-13	AIF1, ALOX5, BGN, C3AR1, CCL5, CCR5, CD86, CDH11, CIITA, CSF2RB, FCGRT, GBP2, GZMA, HCK, HLA-DOA, HLA-DPA1, HLA-DPB1, IGFBP4, IGFBP7, IL10RA, ITPR1, JAM3, LST1, MS4A6A, PSMB9, PTPRC, SAMD9L, SDC2, SNAI2, SPOCK1, TAGAP, TIMP1, TLR3, TNFSF13B, ZFP36	ALOX5, CCL5, CIITA, GBP2, HLA-DOA, HLA-DPA1, HLA-DPB1, MS4A6A, PSMB9, SAMD9L, TAGAP, TLR3, TNFSF13B		CDH11, FCGRT, IGFBP4, IGFBP7, SDC2, SNAI2, TIMP1, ZFP36	BGN, CDH11, IGFBP4, ITPR1, JAM3, SDC2, SNAI2, SPOCK1	AIF1, C3AR1, CCL5, CCR5, CD86, CSF2RB, GZMA, HCK, IL10RA, LST1, MS4A6A, PTPRC
4	apoptosis	2.9E-12	AIF1, ALOX5, ANTXR2, APOL6, AR, ARMC10, BGN, CASP1, CCL5, CCR5, CD2, CD47, CD48, CD53, CDH11, CDKN1A, CIITA, COL1A1, CSF2RB, CTGF, CTSS, CYBB, DKK3, DLC1, EGFR, ENG, ESR1, EVA1C, EYA2, FAS, FBN1, FCER1G, FN1, FOXP1, FSTL1, GIMAP4, GZMA, HCK, HEXB, ID1, ID2, ID3, IFIH1, IGFBP4, IGFBP7, IL15, IRF1, ITGAM, ITPR1, LAIR1, LRIG1, MNDA, MUC16, PMEPA1, PMP22, PTPRC, RHOB, SDC2, SGPP1, SNAI2, SOX17, SPOCK1, SULF2, TIMP1, TLR3, TNFRSF14, TNFSF13B, VCAN, XAF1, ZFP36	ALOX5, APOL6, CASP1, CCL5, CIITA, CTSS, EVA1C, IFIH1, IL15, IRF1, ITGAM, TLR3, TNFSF13B, XAF1		ANTXR2, CDKN1A, COL1A1, CTGF, DLC1, EGFR, ENG, FAS, FOXP1, HEXB, ID1, ID2, ID3, IGFBP4, IGFBP7, RHOB, SDC2, SGPP1, SNAI2, SULF2, TIMP1, ZFP36	BGN, DKK3, DLC1, FBN1, FN1, FSTL1, ID1, ID2, ID3, IGFBP4, ITPR1, PMEPA1, PMP22, RHOB, SDC2, SNAI2, SPOCK1, SULF2	CCL5, CCR5, CD2, CD48, CD53, CSF2RB, CYBB, FCER1G, GZMA, HCK, LAIR1, MNDA, PTPRC
5	necrosis	1.36E-11	ALOX5, ANTXR2, AR, ARMC10, BGN, CASP1, CCL5, CCR5, CD2, CD47, CD48, CD86, CDH11, CDKN1A, CIITA, COL1A1, COL6A1, CSF2RB, CTGF, CTSS, CYBB, DKK3, DPYD, EGFR, ENG, ESR1, EVA1C, EYA2, FAS, FCER1G, FN1, FSTL1, GIMAP4, GZMA, HCK, =ID1, ID2, ID3, IFIH1, IGFBP4, IGFBP7, IL15, IRF1, ITGAM, ITPR1, LAIR1, LRIG1, MEIS1, MUC16, PMEPA1, PMP22, PTPRC, RHOB, ROCK2, SDC2, SGPP1, SNAI2, SOX17, SPOCK1, SULF2, TIMP1, TLR3, TNFRSF14, TNFSF13B, VCAN, XAF1	ALOX5, CASP1, CCL5, CIITA, CTSS, DPYD, EVA1C, IFIH1, IL15, IRF1, ITGAM, TLR3, TNFRSF14, TNFSF13B, XAF1		ANTXR2, CDKN1A, COL1A1, COL6A1, CTGF, EGFR, ENG, FAS, ID1, ID2, ID3, IGFBP4, IGFBP7, RHOB, ROCK2, SDC2, SGPP1, SNAI2, SULF2, TIMP1	BGN, DKK3, FN1, FSTL1, ID1, ID2, ID3, IGFBP4, ITPR1, PMEPA1, PMP22, RHOB, SDC2, SNAI2, SPOCK1, SULF2	CCL5, CCR5, CD2, CD48, CD86, CSF2RB, CYBB, FCER1G, GIMAP4, GZMA, HCK, LAIR1, PTPRC

IPA nomenclature was used for Functional Annotations. Since analysis for the 10-top-output_mixed_and_5 individual revealed only one functional annotation, the results of the top-output_mixed_and_4 individual are additionally shown. The ranking is based on the corresponding IPA-based p-value of the output_mixed; the molecules mapped to the Functional Annotation are listed

**Table 7:** The 10-top-AID/APOBEC signature-linked Functional Annotations identified by systems biology approach (algorithm II; overlap with 3 individual target genes)

Functional Annotations-10-top-output_mixed_and_3_individual								
Pos.	Functional Annotations	Mixed		APOBEC3G	ESR1	ID2	ID3	PTPRC (CD45)
		*P*-value	Molecules	Molecules	Molecules	Molecules	Molecules	Molecules
1	Rheumatic Disease	1.66E-18	ACTA2, AIF1, ALOX5, AR, BGN, C3AR1, CASP1, CCL5, CCR5, CD86, CDH11, CDKN1A, CIITA, COL1A1, COL6A1, CSF2RB, CTSS, FAS, FBN1, FCER1G, FCGRT, FN1, FSTL1, GBP2, GZMA, HCK, HLA-DOA, HLA-DPA1, HLA-DPB1, IGFBP4, IGFBP7, IL10RA, IL15, IRF1, ITGAM, ITPR1, JAM3, LST1, MS4A6A, PSMB9, PTPRC, SAMD9L, SDC2, SNAI2, SPOCK1, TAGAP, TIMP1, TLR3, TLR8, TNFSF13B, ZFP36			ACTA2, CDH11, CDKN1A, COL1A1, COL6A1, FAS, FCGRT, IGFBP4, IGFBP7, SDC2, SNAI2, TIMP1, ZFP36	BGN, CDH11, FBN1, FN1, IGFBP4, ITPR1, JAM3, SDC2, SNAI2, SPOCK1	AIF1, C3AR1, CCL5, CCR5, CD86, CSF2RB, FCER1G, GZMA, HCK, IL10RA, LST1, MS4A6A, PTPRC, TLR8
2	cell movement of leukocytes	8.68E-17	AIF1, ALOX5, AR, BGN, C3AR1, CASP1, CCL5, CCR5, CD2, CD47, CD48, CD86, CDKN1A, CIITA, COL1A1, CTGF, CTSS, CYBB, DOCK2, EGFR, ENG, EPS8, ESR1, FAS, FCER1G, FN1, FYB, HCK, IL10RA, IL15, ITGAM, JAM3, LCP2, PTPRC, RHOB, ROCK2, TIMP1, TLR3, TNFRSF14, TNFSF13B	ALOX5, CASP1, CCL5, CIITA, CTSS, IL15, ITGAM, TLR3, TNFSF13B		CDKN1A, COL1A1, EGFR, ENG, EPS8, FAS, RHOB, ROCK2, TIMP1		AIF1, C3AR1, CCL5, CCR5, CD2, CD48, CD86, CYBB, DOCK2, FCER1G, FYB, HCK, IL10RA, LCP2
3	quantity of leukocytes	1.51E-16	ALOX5, C3AR1, CCL5, CCR5, CD47, CD48, CD84, CD86, CDKN1A, CIITA, CLEC7A, CSF2RB, CTSS, CYBB, DKK3, DOCK2, ESR1, FAS, FCER1G, FOXP1, FYB, HCK, HEXB, ID1, ID2, IL10RA, IL15, IRF1, ITGAM, JAM3, LAIR1, LCP2, MEIS1, PSMB10, PSMB9, PTPRC, SAMSN1, SASH3, SLA, SNAI2, TIMP1, TLR3, TNFSF13B, ZFP36	ALOX5, CCL5, CIITA, CLEC7A, CTSS, IL15, IRF1, ITGAM, PSMB10, PSMB9, TLR3, TNFSF13B		CDKN1A, FAS, FOXP1, HEXB, ID1, ID2, SNAI2, TIMP1, ZFP36		C3AR1, CCL5, CCR5, CD48, CD84, CD86, CSF2RB, CYBB, DOCK2, FCER1G, FYB, HCK, IL10RA, LAIR1, LCP2, PTPRC, SAMSN1, SASH3, SLA
4	cell movement of myeloid cells	4.14E-13	AIF1, ALOX5, AR, C3AR1, CASP1, CCL5, CCR5, CD2, CD47, CD48, CDKN1A, COL1A1, CTGF, CTSS, CYBB, DOCK2, EGFR, ENG, FAS, FCER1G, FN1, HCK, IL15, ITGAM, JAM3, PTPRC, RHOB, ROCK2, TLR3	ALOX5, CASP1, CCL5, CTSS, IL15, ITGAM, TLR3		CDKN1A, COL1A1, EGFR, ENG, FAS, RHOB, ROCK2		AIF1, C3AR1, CCL5, CCR5, CD2, CD48, CYBB, DOCK2, FCER1G, HCK
5	cell movement of phagocytes	9.33E-13	AIF1, ALOX5, AR, C3AR1, CASP1, CCL5, CCR5, CD47, CD86, CDKN1A, COL1A1, CTGF, CTSS, CYBB, DOCK2, EGFR, ENG, EPS8, FAS, FCER1G, FN1, HCK, IL10RA, IL15, ITGAM, JAM3, RHOB, TIMP1, TLR3	ALOX5, CASP1, CCL5, CTSS, ITGAM, TLR3		CDKN1A, COL1A1, EGFR, ENG, EPS8, FAS, RHOB, TIMP1		AIF1, C3AR1, CCL5, CCR5, CD86, CYBB, DOCK2, FCER1G, HCK, IL10RA
6	cell death	7.48E-11	AIF1, ALOX5, ANTXR2, APOL6, AR, ARMC10, BGN, CASP1, CCL5, CCR5, CD2, CD47, CD48, CD53, CD86, CDH11, CDKN1A, CIITA, COL1A1, COL6A1, CSF2RB, CTGF, CTSS, CYBB, DKK3, DLC1, DPYD, EGFR, ENG, ESR1, EVA1C, EYA2, FAS, FBN1, FCER1G, FN1, FOXP1, FSTL1, GIMAP4, GZMA, HCK, HEXB, ID1, ID2, ID3, IFIH1, IGFBP4, IGFBP7, IL15, IRF1, ITGAM, ITPR1, LAIR1, LCP2, LRIG1, MEIS1, MNDA, MUC16, PMEPA1, PMP22, PTPRC, RHOB, ROCK2, SDC2, SGPP1, SNAI2, SOX17, SPOCK1, SULF2, TFEC, TIMP1, TLR3, TNFRSF14, TNFSF13B, VCAN, XAF1, ZFP36	ALOX5, APOL6, CASP1, CCL5, CIITA, CTSS, DPYD, EVA1C, IFIH1, IL15, IRF1, ITGAM, TLR3, TNFRSF14, TNFSF13B, XAF1		ANTXR2, CDKN1A, COL1A1, COL6A1, CTGF, DLC1, EGFR, ENG, FAS, FOXP1, HEXB, ID1, ID2, ID3, IGFBP4, IGFBP7, RHOB, ROCK2, SDC2, SGPP1, SNAI2, SULF2, TIMP1, ZFP36		AIF1, CCL5, CCR5, CD2, CD48, CD53, CD86, CSF2RB, CYBB, FCER1G, GIMAP4, GZMA, HCK, LAIR1, LCP2, MNDA, PTPRC
7	metastasis	1.05E-10	ACTA2, AR, CASP1, CD47, CD86, CDKN1A, CTGF, CYBB, EGFR, ESR1, FN1, FOXP1, ID1, ID2, ID3, IGFBP7, IL15, IRF1, JAM3, MEIS1, NID2, PAPSS2, PRRX1, RAB31, RHOB, TIMP1, TLR8, VCAN		AR, CD47, ESR1, MEIS1	ACTA2, CDKN1A, CTGF, EGFR, FOXP1, ID1, ID2, ID3, IGFBP7, PAPSS2, RAB31, TIMP1	FN1, ID1, ID2, ID3, JAM3, PRRX1	
8	triple-negative breast cancer	1.15E-10	AR, CDH11, CDKN1A, CTGF, DLC1, EGFR, EPS8, ESR1, FAS, HCK, IRF1, LAMB1, TIMP1, WFDC2		AR, ESR1, WFDC2	CDH11, CDKN1A, CTGF, DLC1, EGFR, EPS8, FAS, TIMP1	CDH11, DLC1, LAMB1	
9	binding of cells	1.52E-10	AR, BGN, CCL5, CCR5, CD2, CD47, CD48, CD84, CD86, CLEC7A, CSF2RB, ENG, FAS, FN1, FYB, HCK, HS3ST1, ITGAM, JAM3, LCP2, MUC16, PTPRC, RHOB, SDC2, SNX9			ENG, FAS, HS3ST1, RHOB, SDC2, SNX9	BGN, FN1, JAM3, RHOB, SDC2	CCL5, CCR5, CD2, CD48, CD86, CSF2RB, FYB, HCK, LCP2, PTPRC
10	Lymphocyte migration	2.14E-10	ALOX5, CCL5, CCR5, CD2, CD47, CD48, CD86, COL1A1, CTSS, DOCK2, EGFR, ENG, FAS, FN1, FYB, IL15, JAM3, TIMP1, TNFRSF14, TNFSF13B	ALOX5, CCL5, IL15, TNFSF13B		COL1A1, EGFR, ENG, FAS, TIMP1		CCL5, CCR5, CD2, CD48, CD86, DOCK2, FYB

IPA nomenclature was used for Functional Annotations. Since analysis for the 10-top-output_mixed_and_4 individual revealed only 5 functional annotations, the results of the 10-top-output_mixed_and_3 individual are additionally shown. The ranking is based on the corresponding IPA-based p-value of the output_mixed; the molecules mapped to the Functional Annotation are listed

### Data-driven signature-associated upstream regulators

To include the biological information for the higher-level overview, the IPA-based analysis for Upstream Regulators was performed. In this case, both algorithms I and II were used. The 10-top most significantly over-represented regulators, when applying algorithm I, were LPS, IFNalpha, IFNgamma, TGFbeta1, TNF, IL10, STAT3, IL6, IL13, and tretinoin (Table 8). Of note, 6 out of 10 regulators derived from the output_mixed were found in at least one output_individual within the 10-top positions (with p-values all < 0.05). The strongest overlap between the 10-top-output_mixed and _individual was observed for *APOBEC3G* (4/10) and *PTPRC/CD45* (2/10); in contrast, no overlap was found for *ESR1*, thus the observed distribution is similar to the one described for Canonical Pathways analysis (Table 4). Complementary, the heat map and pie chart illustrate the total overlap patterns between output_mixed and output_individuals (Figures S8, C and S9, C). When applying the algorithm II, the following additional molecules were identified such as APOE, CD44, TECAM1, Ifi204, alpha Catenin, INFbeta1, TGFBR1, SPI1, and CD40 (Additional file 1: Tables S16 and S17).

**Table 8:** The 10-top-AID/APOBEC signature-linked Upstream Regulators identified by systems biology approach (algorithm I)

Upstream Regulators-10-top-output_mixed^a^				Upstream Regulators output_individual^b^									
				APOBEC3G		ESR1		ID2		ID3		PTPRC (CD45)	
Pos.	Upstream Regulator	*P*-value	Molecules	Pos.	Molecules	Pos.	Molecules	Pos.	Molecules	Pos.	Molecules	Pos.	Molecules
1	lipopolysaccharide	2.52E-27	ACTA2, ALOX5, APOBEC3F, APOBEC3G, APOL6, AR, BGN, C3AR1, CARD6, CASP1, CCL5, CCR5, CD48, CD53, CD86, CDH11, CDKN1A, CIITA, CLEC7A, COL1A1, COL5A1, COL6A1, CSF2RB, CTGF, CYBB, DKK3, FAS, FBN1, FCER1G, FCGRT, FN1, FYB, GBP2, GZMA, HCK, ID2, IFIH1, IGFBP4, IL10RA, IL15, IRF1, ITGAM, KIAA1551, LAMB1, LCP2, LST1, NID1, NID2, PAPSS2, PLEK, PSMB10, PSMB9, RARRES3, RHOB, SAMSN1, TFEC, TIMP1, TLR3, TLR8, TNFSF13B, TRIM22, VCAN, XAF1, ZFP36										
2	Interferon alpha	3.48E-19	APOBEC3F, APOBEC3G, APOL3/APOL4, C3AR1, CASP1, CCL5, CCR5, CD86, CDKN1A, CIITA, CSF2RB, EGFR, FAS, GBP2, IFIH1, IGFBP4, IL10RA, IL15, IRF1, ITGAM, MNDA, PSMB9, RARRES3, TLR3, TLR8, TNFSF13B, TRIM22	4	APOBEC3F, APOBEC3G, APOL3/APOL4, CASP1, CIITA, IL15, IRF1, TLR3, TNFSF13B			591	CDKN1A, IGFBP4			146	CSF2RB, TLR8
3	IFNG	7.59E-18	AIF1, APOBEC3G, APOL6, CARD6, CASP1, CCL5, CCR5, CD2, CD86, CDKN1A, CIITA, COL1A1, CSF2RB, CTGF, CTSS, CYBB, FAS, FCER1G, FN1, GBP2, GMPR, HLA-DOA, ID1, IFIH1, IGFBP4, IL10RA, IL15, IRF1, ITGAM, LCP2, LST1, MNDA, PLEK, PSMB10, PSMB9, RARRES3, RHOB, SNAI2, TIMP1, TLR3, TLR8, TNFRSF14, TNFSF13B, TRIM22, ZFP36	1	APOL6, CARD6, CASP1, CCL5, CIITA, CTSS, GBP2, IFIH1, IL15, IRF1, ITGAM, PSMB10, PSMB9, RARRES3, TLR3, TNFSF13B, TRIM22							7	AIF1, CCL5, CCR5, CD2, CD86, CYBB, FCER1G
4	TGFB1	8.38E-18	ACTA2, ALOX5, BGN, CASP1, CCL5, CCR5, CD86, CDH11, CDKN1A, CIITA, COL1A1, COL5A1, COL6A1, COL6A2, CTGF, CTSS, CYBB, DKK3, DOCK2, EMILIN1, ENG, FAS, FBN1, FCER1G, FN1, GMPR, GZMA, HEXB, ID1, ID2, ID3, IFIH1, IGFBP4, IGFBP7, IL10RA, IL15, IRF1, ITGAM, ITPR1, PDLIM5, PLOD1, PMEPA1, PTPRC, RAB31, RHOB, SNAI2, SPOCK1, TIMP1, TNFRSF14, TNFSF13B, VCAN, ZFP36	161	ALOX5, CCL5, IRF1, ITGAM			3	ACTA2, CDH11, CDKN1A, COL1A1, CTGF, ENG, FAS, HEXB, ID1, RAB31, RHOB, SNAI2, TIMP1, ZFP36	3	BGN, CDH11, COL5A1, FBN1, FN1, ID1, PMEPA1, RHOB, SNAI2, SPOCK1		
5	TNF	1.25E-16	ABR, AEBP1, ALOX5, AR, BGN, CARD16, CARD6, CASP1, CCL5, CCR5, CD47, CD86, CDH11, CDKN1A, CIITA, COL1A1, CSF2RB, CTGF, CTSS, CYBB, EGFR, ENG, ESR1, FAS, FCER1G, FCGRT, FN1, GBP2, HEXB, ID1, ID3, IFIH1, IGFBP4, IL10RA, IL15, IRF1, ITGAM, ITPR1, NID1, PPAP2A, PSMB10, PSMB9, RARRES3, SDC2, TIMP1, TLR3, TLR8, TNFSF13B, ZFP36	7	CASP1, CCL5, CTSS, GBP2, IL15, IRF1, ITGAM, PSMB10, PSMB9, RARRES3, TLR3, TNFSF13B			69	CDH11, CDKN1A, EGFR, FAS, HEXB, NID1, PPAP2A, TIMP1	52	AEBP1, CDH11, FN1, ITPR1, NID1, PPAP2A	181	CCL5, CCR5, CD86, CYBB
6	IL10	1.91E-15	ACTA2, CCL5, CCR5, CD2, CD86, CDKN1A, CIITA, CLEC7A, COL1A1, CSF2RB, CTSS, FAS, FCER1G, GZMA, IGFBP4, IL10RA, IRF1, PSMB9, TIMP1, TLR3, TLR8, TNFSF13B, VCAN, ZFP36	87	CCL5, CIITA, TLR3			97	ACTA2, CDKN1A, TIMP1, ZFP36			45	CCL5, CD2, CD86
7	STAT3	5.98E-15	CASP1, CCL5, CCR5, CD86, CDKN1A, CIITA, COL5A1, ESR1, FAS, FCER1G, FN1, GBP2, ID2, IFIH1, IRF1, ITGAM, PSMB9, SMAD9, TIMP1, TLR3, TRIM14, TRIM22, VCAN, XAF1, ZFP36	9	CASP1, CIITA, GBP2, IRF1, ITGAM, PSMB9, TLR3, XAF1			461	CDKN1A, TIMP1, ZFP36	106	FN1, SMAD9, VCAN		
8	IL6	2.87E-14	BGN, CASP1, CCL5, CCR5, CD48, CD53, CD86, CDKN1A, CIITA, CSF2RB, CTGF, CYBB, EGFR, FAS, FCGRT, FN1, GBP2, ID1, ID2, IGFBP4, IL15, IRF1, ITGAM, PSMB10, PSMB9, PTPRC, RNASE6, TIMP1, TLR3, TLR8	13	CASP1, CIITA, GBP2, IRF1, ITGAM, PSMB9, TLR3			211	CDKN1A, ID1, ID2, TIMP1				
9	IL13	7.28E-14	C3AR1, CASP1, CCL5, CCR5, CD48, CD86, CLEC7A, COL1A1, COL6A2, CTGF, CTSS, CYBB, EGFR, FAS, FGD2, HOMER2, NID1, SAMSN1, SLA, SNAI2, TFEC, TIMP1, TNFSF13B	70	CASP1, CCL5, CTSS, FGD2			512	FAS, NID1, TIMP1			1	C3AR1, CCL5, CCR5, CD48, CD86, CYBB, SAMSN1, SLA
10	tretinoin	1.15E-13	C3AR1, CASP1, CCR5, CD53, CD86, CDKN1A, COL1A1, CTGF, CYBB, DDX60L, DLC1, EGFR, EYA2, FCER1G, FN1, GZMA, HS3ST1, ID1, ID2, ID3, IFIH1, IGFBP4, IGFBP7, IL10RA, IRF1, ITGAM, LAMB1, MEIS1, MNDA, NID2, PLEK, PSMB9, RARRES3, SAMD9L, SLA, SMAD9, SOX17, THSD4, TIMP1, TLR3, TRIM22, XAF1										

^a^The 10-top-output_mixed results are shown; the ranking is based on the corresponding IPA-based p-value; the molecules associated with the corresponding Upstream Regulator are listed

^b^Position of the Upstream Regulator within the output_individual for each target gene, the molecules associated with the Upstream Regulator are indicated. Color code: grey, within the 10-top-output_individual; white, outside the 10-top-output_individual. Empty cell: the corresponding Upstream Regulator was not significant in output_individual

## Discussion

### Multigene profiling and survival models

The herein presented study is, to our knowledge, the first one linking expression of the entire *AID*/*APOBEC* family and interacting genes with clinical outcome with respect to survival of cancer patients. High efforts are invested in the field of cancer research to evaluate the applicability of gene expression for the use in risk prediction; nevertheless, no established standard is available for the implemented methodology to study gene expression profiles in the pathophysiology of disease. Different approaches have certain advantages and disadvantages. Data-driven approaches using curated microarray expression data from various studies offer the advantage of a transcriptome-wide screening, but face a lack of sensitivity for very low-level expressed genes and/or the specificity for genes with high sequence similarity; the latter two aspects are fully relevant for AID and APOBEC3 subfamily, respectively, as discussed herein and by others [13]. A knowledge-driven approach, where the composition of a gene signature characterizing certain biological aspects is assembled based on data mining followed by real-time PCR-based gene expression profiling of clinical specimens has by definition the advantage of high sensitivity and reproducibility as real-time PCR methodology is the gold standard in expression profiling. To maximize the outcome, we applied herein a rational integration of both approaches.

We structured the study by three consecutive analytical modules: (i) multigene-based expression profiling, (ii) statistical modeling for survival prediction and (iii) delineation of AID/APOBEC-associated gene network(s) and pathways by applying bioinformatics tools (as summarized in Fig. 1). Each module resulted in novel outcomes with respect to the pathophysiology of ovarian cancer; their combination in turn provided an advantageous comprehensive overview. The herein established study algorithm, named by us as MuSiCO (from **Mu**ltigene **Si**gnature to the Patient-Orientated **C**linical **O**utcome), can be further applied for any gene-/pathway-/disease-related signature and any type of tumor under investigation.

One objective here is the development of proper multivariable-based models for evaluating patient prognosis on the basis of the patient-specific gene expression data sets. Although proceedings in whole genome analysis have triggered the developments of statistical methods for survival models [68], multivariable Cox regression analysis of multigene-based prognostic models is still a challenging task based on the following aspects. Classical statistical regression methods based on maximum likelihood have been widely used when the number of outcome events highly exceeds the number of variables. In practice, however, we frequently face restricted availability of well-characterized high quality clinical samples. Thus, in case of a multigene approach, a relatively large number of profiling-derived variables is often accompanied by relatively few outcome events (with respect to OS this is the number of cancer-related deaths and not the number of patients in the examined cohort). Given that, herein we established and applied state-of-the-art algorithms for multivariable modeling allowing (i) to reduce the risk of getting an overoptimistic or overfitted survival model and, thus, to increase predictive accuracy; (ii) to be wrapped in a cross-validation loop in order to estimate the performance of the model and, importantly, to compare the prognostic power and accuracy between different models; and (iii) to be able to rank the contributions of individual variables to predictions by their importance. We explicitly applied the leave-one-out strategy of cross-validation instead of dividing the data set in to a training and test set. We believe that the latter approach is inferior for several reasons: (i) in a situation of a relatively small number of events, it would waste a lot of data which would be needed to obtain more stable estimates; (ii) it can easily be manipulated by selecting a split that yields optimal results. To characterize the performances of our prognostic models, we used PEV, c-index, and p-value, and these parameters could be used to compare models within one study but also to make comparisons between independent studies and laboratories. Thereby, by usage of those appropriate measures, the truly independent validation can be performed by an independent group of researchers and using an independent cohort of patients.

For the examined cohort of patients we herein confirmed strong prognostic relevance of clinical risk factors and showed that six clinicopathological variables such as peritoneal carcinomatosis, age, histology, FIGO stage, residual disease, and grading can be assembled into a survival model which has prognostic power for both OS and PFS. Importantly, by inclusion of the AID/APOBEC signature-based variables, the combined model significantly improved the prognostication of OS. Furthermore, several of the gene profiling-derived variables within the combined model such as *ID3*, *PTPRC/CD45*, *AID*, *APOBEC3G*, and *ID2* exceed the prognostic impact of some clinicopathological variables to the model. Remarkably, in both models (ridge and LASSO) *ID3* was ranked at the 1st position overcoming the impact of all six clinical and profiling-derived variables. Given in addition the strong significance of *ID3* in univariate Cox regression analysis, the data nominate *ID3* as prognostic factor for OS. Moreover, since higher *ID3* mRNA levels were associated with poor survival, further functional studies are needed to validate whether ID3 might in addition act as a “driver” of pathogenesis of ovarian cancer. ID molecules (ID1-4) are functional inhibitors/antagonists of the basic helix-loop-helix transcription factors and thus control the expression of multiple targets including among others AID [63, 64]. The critical implications of dysregulated IDs in multiple cancer hallmarks are highly recognized (reviewed in [69]) including contribution to pathomechanisms of ovarian cancer [38, 70–72]. Small molecule inhibitors of IDs are in development and might be considered as novel combinatorial therapeutic approach for treatment of cancer [73, 74].

For several cancer types (breast, bladder, cervical, head and neck and lung) an APOBEC mutation pattern was identified and the APOBEC-mediated mutagenesis was found to correlate with *APOBEC* mRNA levels, particularly with *APOBEC3B* [13, 36]. Furthermore, during our data mining, the study of Leonard et al. was published [40] showing elevated expression of *APOBEC3B* in the majority of ovarian cancer cell lines examined and in a subset of high-grade primary ovarian cancer in comparison to the normal ovarian or fallopian tube epithelial cells and non-malignant ovarian tissues, respectively. Although a direct comparison of expression levels between serous tumor samples and normal ovarian tissues is the point of debates, which has been as well discussed by the authors, the accompanied functional studies revealed a positive association between *APOBEC3B* expression in cancer tissues from 16 patients and elevated levels of transversion mutations, thus, suggesting a contributing role of *APOBEC3B* in genomic instability attributed to ovarian cancer. Against the logical expectations, our data did not reveal a prognostic relevance of *APOBEC3B* mRNA levels in the examined cohort of patients when assessed by univariate Cox regression analysis. In the multivariable prognostic models such as *AID/APOBEC* or *Combined*, according to standardized regression coefficients-based ranking *APOBEC3B* was assigned to the positions 7 and 24, respectively, and, thus, showed a moderate/minimal impact on the prognostic ability of the models. Our data, however, does not exclude any additional ways of regulation of APOBEC3B activity in a patient-specific manner with respect to disease pathobiology. Besides *AID*, among *APOBEC3* subfamily members, *APOBEC3G* contributed to the prognostic models (both ridge and LASSO). This might indicate that in tumor cells during the cancer progression the interplay between individual APOBEC3 family members plays a contributing role. Additionally, one should consider the complex composition of the ovarian cancer tissues used for the gene expression profiling, which besides the tumor cells includes the tumor stroma with significant component attributed to infiltrated immune cell populations; thus, the *AID/APOBEC* mRNA expression values likely reflex the sum from all positive cells. Indeed, on the one side, the herein performed expression analysis of the signature genes using a wide range of ovarian cancer cell lines showed that those innate/adaptive immunity-related genes might as well be expressed in ovarian tumor cells *per se*; our data are generally in line with the profiling results reported recently [40]. On the other side, the correlation analysis of multigene-derived data sets across ovarian cancer tissues revealed strong positive association between *PTPRC/CD45*, the classical immune cell marker, and *APOBEC3* subfamily members such as *A3C*, *A3D*, *A3H*, and the prognostically relevant *A3G* as well as *AID*. Furthermore, although weaker, a positive association was observed with *PAX5*, the B-cell transcription factor. These data suggest that, besides expression by tumor cells, certain contributions from immune cells, including B lymphocytes, to the total mRNA expression levels of individual *APOBECs* indeed might take place which thereby impacts the prognostic power of the model. Of note, no significant correlation was found between *PTPRC/CD45* and *APOBEC3B*, thus, likely excluding the major impact of the CD45-positive immune cells to this variable.

It is important to emphasize that within the prognostic model *APOBEC3G* behaves as a protective factor with potential anti-tumor action since higher *APOBEC3G* mRNA expression levels were associated with better clinical outcome in respect of OS. Previous cell-based studies showed that *APOBEC3G* does not fall into the subclass of *APOBECs* (which among others includes *APOBEC3B*) grouped based on mutational specificity for TC motifs [75] suggesting somewhat different *APOBEC3G*-mediated biological consequences. Considering the APOBEC3 functions in virus, naked foreign DNA or retrotransposon restriction, a potential association between the APOBEC expression, the APOBEC-mediated cancer-related mutagenesis and the viral infection/viral carcinogenesis is appealing and was discussed recently, when two cancer types, cervical and head and neck cancer, which are highly associated with human papillomavirus, HPV, were found among those six types with strong enrichment of APOBEC-mediated mutagenic patterns [36, 76]; HPV in turn is one of the known APOBEC3-targeted viruses [77, 78] (besides well-studied HIV-1, the list includes HTLV, HCV, HBV, HPV, HSV-1, and EBV). Such association in ovarian cancer is currently not known.

### Data-driven disease-relevant pathways

**Fig. 4:**
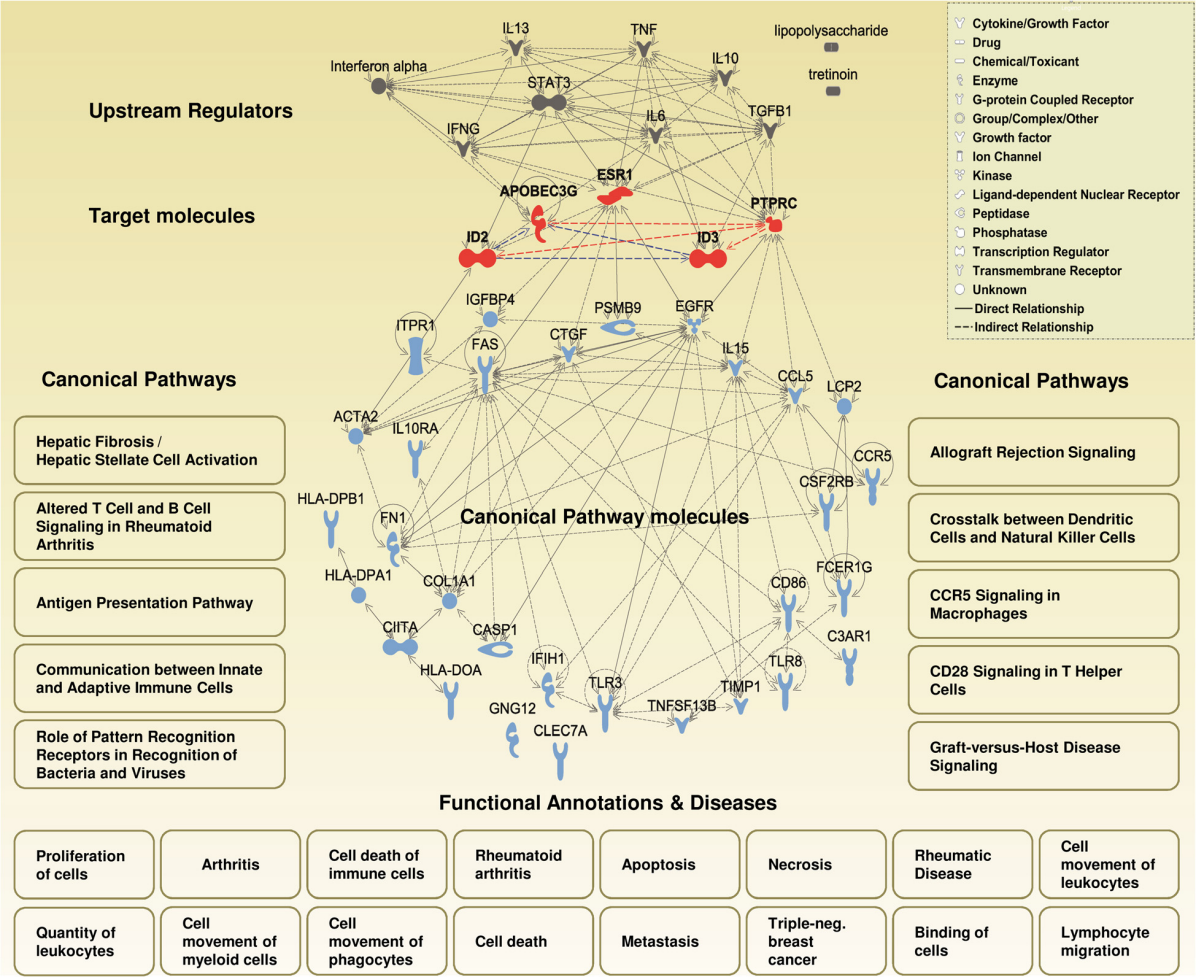
Representation of the top Canonical Pathways, Functional Annotations and Upstream Regulators detected upon analysis of target genes and their co-regulated genes. A reconstructed gene network was created using the Ingenuity Pathway Analysis Software (IPA) on the basis of the target molecules, the molecules from the 10-top Canonical Pathways (see Table 4, *Molecules*) and the 10-top Upstream Regulators (see Table 8). Solid lines in grey display the IPA-identified direct interactions between the molecules; dashed lines display indirect interactions. The multigene approach-based correlation analysis was used to find additional biological associations between the target genes. Statistically significant study-based associations (SPSS program, Additional file 1: Table S5) are displayed by *dashed lines*; *red* for correlation coefficient ≥ 0.6, *p* < 0.001; *blue* for correlation coefficient < 0.6, *p* < 0.001. The 10-top Canonical Pathways are listed according to the IPA-based ranking (from *left* to *right*). For a complete overview, also the most significant Functional Annotations & Diseases are shown (see Tables 5, 6 and 7)

The third analytical module applied herein allowed us to extend the signature- and modeling-based knowledge and dissect potential mechanisms/pathways/factors contributing to disease pathogenesis and patient survival. The reconstructed network was created and visualized using the IPA software on the basis of the target molecules defined by prognostic modeling and the molecules from co-expressed genes derived from the 10-top Canonical Pathways and Upstream Regulators (Fig. 4). This integration and visualization of both experimental and *in silico* microarray-based data illustrate the existence of mutual interconnections between four target genes such as *PTPRC/CD45*, *ID3*, *APOBEC3G*, *ID2* and point to more separate biological function(s) of the node around *ESR1* in advanced stage serous ovarian cancer.

Generally, the 10-top Canonical Pathways contain molecules which are characteristic for tissue remodeling/fibrotic pathway, altered immune response including antigen presentation mechanisms, and communication between various immune cell populations involved in innate and adaptive immune responses. It gives a link to autoimmune disorders with inflammatory background as rheumatoid arthritis and to transplant rejection by the recipient’s immune system and, unsuspectedly, does not highlight the cancer-related processes. Notably, among the top Canonical Pathways, the first top-ranked was Hepatic Fibrosis/Hepatic Stellate Cell Activation based on such molecules as COL1A1, IGFBP4, CCR5, FN1, CTGF, TIMP1, ACTA2, IL10RA, CCL5, FAS, EGFR. The significant association between fibrosis and the clinical outcome of ovarian cancer patients was observed recently by others, although it was identified by applying completely different approaches such as miRNA screening or histological examinations, respectively [79, 80]. Surprisingly, the same pathway was identified as one of the most relevant canonical pathways in granulosa cells from bovine ovarian follicles during atresia, which represents one of the physiological processes in healthy ovaries [81]. Thus, aberrant modulation of the pathway’s underlying molecules might turn the physiological processes to the direction of malignant transformation.

Multiple Canonical Pathways within the identified 10-top are linked to the antigen processing and presentation machinery and antigen recognition by lymphocytes (Fig. 4 and Table 4, pos. 2, 3, 6, 9, and 10); HLA class II transcripts are among the molecules underlying these pathways. In this respect it is interesting to note that the recent study by Yoshihara et al. [82] identified the antigen presentation pathway to be significantly modulated (as estimated by downregulation of HLA class I molecules) in a high risk group compared with a low risk group of patients with high-grade serous ovarian cancer.

The top Functional Annotations & Diseases and Upstream Regulators of the AID/APOBEC-associated network reconstruction further indicate the particular significance of immunity, aberrant immunity/autoimmunity and inflammation. Rather unexpectedly, the categories such as rheumatic diseases, arthritis, rheumatoid arthritis were top-ranked together with more broad functions such as proliferation of cells, binding of cells, cell movement including movement of various immune cell subsets (lymphocytes, myeloid cells, phagocytes), and quantity of leukocytes. The identified association with rheumatoid arthritis – the progressive inflammatory autoimmune disorder – further points out to the importance/relevance of inflammation and suggests an autoimmune phenomenon as potential novel aspect in pathophysiology of ovarian cancer. It is important to note that the cytokines most directly implicated in the pathophysiology of rheumatoid arthritis are proinflammatory TNFalpha and IL-6 [83]; herein these molecules are ranked within the 10-top most significantly over-represented regulators. Intriguingly, both TNFalpha and in particular IL-6 have been previously shown to promote epithelial ovarian tumorigenesis and cancer progression (reviewed in [84]). Numerous preclinical and translational studies emphasize the rational of targeting the IL-6/IL-6-signaling pathways in cancer, considering among others ovarian carcinoma, either as single treatment or in combination with other chemotherapeutic drugs [85, 86]; reviewed in [87]. The present study supports this notion. Furthermore, it proposes for consideration/reconsideration the assessment of IL-6 and other markers of arthritis including systemic autoantibodies and, as proposed previously, C-reactive protein [88] for monitoring the disease and therapy response in ovarian cancer. Noteworthy, data of recent epidemiological studies suggest an increased risk of developing ovarian cancer for patients with rheumatoid arthritis at advanced stage of disease; for entire, unstratified patient group the association was reported to be inverse [89].

Besides that, the data unexpectedly suggest that the therapeutic regiments considered for treatment of HIV by mechanism(s) of enhancing the APOBEC3G expression/activity might be added for consideration for the stratified group of high risk patients with serous ovarian cancer. Among those are IFNalpha and novel IFN-related mimetics preserving beneficial antiviral roles while minimizing negative effects [90]. It is important to emphasize that based on different argumentations and accenting the immunomodulatory and antiproliferative activities of IFNs family, the attempts have been already made to establish IFN as a standard in the treatment of ovarian cancer [91, 92]. However, the results were not monosemantic among the various clinical trials. This stresses the complexity of the disease and strongly indicates the necessity to stratify the patient population prior to drug application. Small molecules as agonists or antagonists [93, 94], which are able to modulate specifically the APOBEC3G and APOBEC3B levels and activities, respectively, can be as well considered as starting points for further development of combinatorial drug applications in ovarian cancer. Still, there is much to clarify regarding the expression patterns of APOBEC3G (as well as APOBEC3B) on the protein transcript levels in respect of the immune contexture and tumor anatomy applying the methodology of computerized assessment of large-scale ovarian cancer tissue sections (examples in [95, 96]).

We herein used a well-characterized patient cohort at advanced stage of ovarian cancer. This cohort reflects the current clinical situation in the medical care of ovarian cancer patients as most cases of ovarian cancer are diagnosed at advanced stages of disease due to inconspicuous symptoms and lack of reliable biomarkers [97]. Based on that, the data-driven conclusion likely suggests the contribution of *AID/APOBEC*-triggered mechanisms to the disease progression. However, their cancer-causing role cannot be as well excluded.

## Conclusions

The herein defined analysis algorithm, MuSiCO, allows to establish a link between AID/APOBEC-associated gene expression profiles and patient survival, and to further delineate novel disease-associated pathways/networks. Based on the results of complex multivariable modeling, we propose a novel strategy for risk assessment of patients with primary ovarian cancer by integration of AID/APOBEC signature-based data sets and clinical risk factors into a *combined* survival model. We evaluated the performance of various prognostic models based on PEV, c-index and p-value. We propose to use these parameters to compare models not only within one study but also to make comparisons between independent studies and laboratories. Furthermore, we reconstructed a gene regulatory network on the basis of target molecules defined by prognostic modeling and the molecules from co-expressed genes derived from curated transcriptome-based expression data from the serous ovarian cancer-based studies. These findings link the expression pattern of AID/APOBEC-associated genes with remodeling/fibrotic pathways, altered immune response, and autoimmune disorders with inflammatory background (Fig. 4), and propose for a consideration of potential novel biomarkers and/or targets and therapeutic regiments, although with a strong indication for necessity to stratify the patient population prior to drug application. Among them are APOBEC3G, AID, ID3, IL-6, IFNalpha and novel IFN-related mimetics. This study additionally suggest to consolidate the acquired knowledge and research efforts in the fields of virology and cancer research around AID/APOBECs expression and functionality as well as drug targeting and drugs in development.

## Abbreviations

AID (or AICDA), activation-induced cytidine deaminase; APOBEC, apolipoprotein B mRNA editing enzyme, catalytic polypeptide-like; C-index, concordance index; EOC, epithelial ovarian cancer; FIGO, International Federation of Obstetrics and Gynecology; HR, hazard ratio; CI, confidence interval; IPA, Ingenuity Pathway Analysis; MuSiCO, from Multigene Signature to the Patient-Orientated Clinical Outcome; OS, overall survival; PEV, proportion of explained variation; PFS, progression free survival

## Additional file

Additional file 1: The following additional data are available with the online version of this paper. **Figure S1.** Graphical view of the expression range of target genes used in GENEVESTIGATOR-based analysis. **Figure S2.** Correlation analysis of reference HKGs expression values in microarray data sets. **Figure S3.** Figure shows the impact of individual variables on survival prediction of the multivariable model (LASSO) for OS if predictors are changed by +1 SD. **Figure S4.** Figure shows LASSO-based Kaplan/Meier estimates for OS and PFS. **Figure S5.** Figure shows expression profiles of the AID/APOBEC-based multigene signature in ovarian cancer cell lines. **Figure S6.** Figure shows the extended analysis of expression profiles of the AID/APOBEC signature genes in a wide range of ovarian cancer cell lines (*n* = 55). **Figure S7.** Shown is the result of hierarchical clustering for individual genes composing the AID/APOBEC signature across arrays/samples of 55 ovarian cancer cell lines. **Figure S8.** The heat maps show the distribution of the Canonical Pathways/Functional Annotations/Upstream Regulators between corresponding target genes. **Figure S9.** The pie charts indicate the overlap of Canonical Pathways/Functional Annotations/Upstream Regulators between output_mixed and output_individual. **Table S1.** Real-time PCR primer sequences. **Table S2.** Real-time PCR primers. **Table S3.** Genes composing the AID/APOBEC multigene signature. **Table S4.** Univariate Cox regression analysis of clinicopathological variables and gene profiling-derived data sets for OS and PFS. **Table S5.** Correlation analysis for the AID/APOBEC multigene-derived variables. **Table S6.** Multivariable models (ridge) for PFS. **Table S7.** Comparative analysis of multivariable models (LASSO) for prognostication of OS and PFS. **Table S8.** Multivariable models (LASSO) for OS. **Table S9.** Multivariable models (LASSO) for PFS. **Table S10.** Top 50 Affymetrix probe sets co-regulated with *APOBEC3G*. **Table S11.** Top 50 Affymetrix probe sets co-regulated with *ESR1*. **Table S12.** Top 50 Affymetrix probe sets co-regulated with *ID2*. **Table S13.** Top 50 Affymetrix probe sets co-regulated with *ID3.* **Table S14.** Top 50 Affymetrix probe sets co-regulated with *PTPRC/CD45.* **Table S15.** 10-top-AID/APOBEC signature-linked Canonical Pathways. **Table S16.** Top-AID/APOBEC signature-linked Upstream Regulators. **Table S17.** 10-top-AID/APOBEC signature-linked Upstream Regulators. (PDF 4761 kb)
