# Supplementary material for: AID/APOBEC-network reconstruction identifies pathways associated with survival in ovarian cancer
Source: BMC Genomics. 2016 Aug 16;17:643. doi: 10.1186/s12864-016-3001-y (PMC4986275; doi:10.1186/s12864-016-3001-y)
Supplement: Additional file 1: — The following additional data are available with the online version of this paper. Figure S1. Graphical view of the expression range of target genes used in GENEVESTIGATOR-based analysis. Figure S2. Correlation analysis of reference HKGs expression values in microarray data sets. Figure S3. Figure shows the impact of individual variables on survival prediction of the multivariable model (LASSO) for OS if predictors are changed by +1 SD. Figure S4. Figure shows LASSO-based Kaplan/Meier estimates for OS and PFS. Figure S5. Figure shows expression profiles of the AID/APOBEC-based multigene signature in ovarian cancer cell lines. Figure S6. Figure shows the extended analysis of expression profiles of the AID/APOBEC signature genes in a wide range of ovarian cancer cell lines (n = 55). Figure S7. Shown is the result of hierarchical clustering for individual genes composing the AID/APOBEC signature across arrays/samples of 55 ovarian cancer cell lines. Figure S8. The heat maps show the distribution of the Canonical Pathways/Functional Annotations/Upstream Regulators between corresponding target genes. Figure S9. The pie charts indicate the overlap of Canonical Pathways/Functional Annotations/Upstream Regulators between output_mixed and output_individual. Table S1. Real-time PCR primer sequences. Table S2. Real-time PCR primers. Table S3. Genes composing the AID/APOBEC multigene signature. Table S4. Univariate Cox regression analysis of clinicopathological variables and gene profiling-derived data sets for OS and PFS. Table S5. Correlation analysis for the AID/APOBEC multigene-derived variables. Table S6. Multivariable models (ridge) for PFS. Table S7. Comparative analysis of multivariable models (LASSO) for prognostication of OS and PFS. Table S8. Multivariable models (LASSO) for OS. Table S9. Multivariable models (LASSO) for PFS. Table S10. Top 50 Affymetrix probe sets co-regulated with APOBEC3G. Table S11. Top 50 Affymetrix probe sets co-regulated with ESR1. Table S12. [file 12864_2016_3001_MOESM1_ESM.pdf]

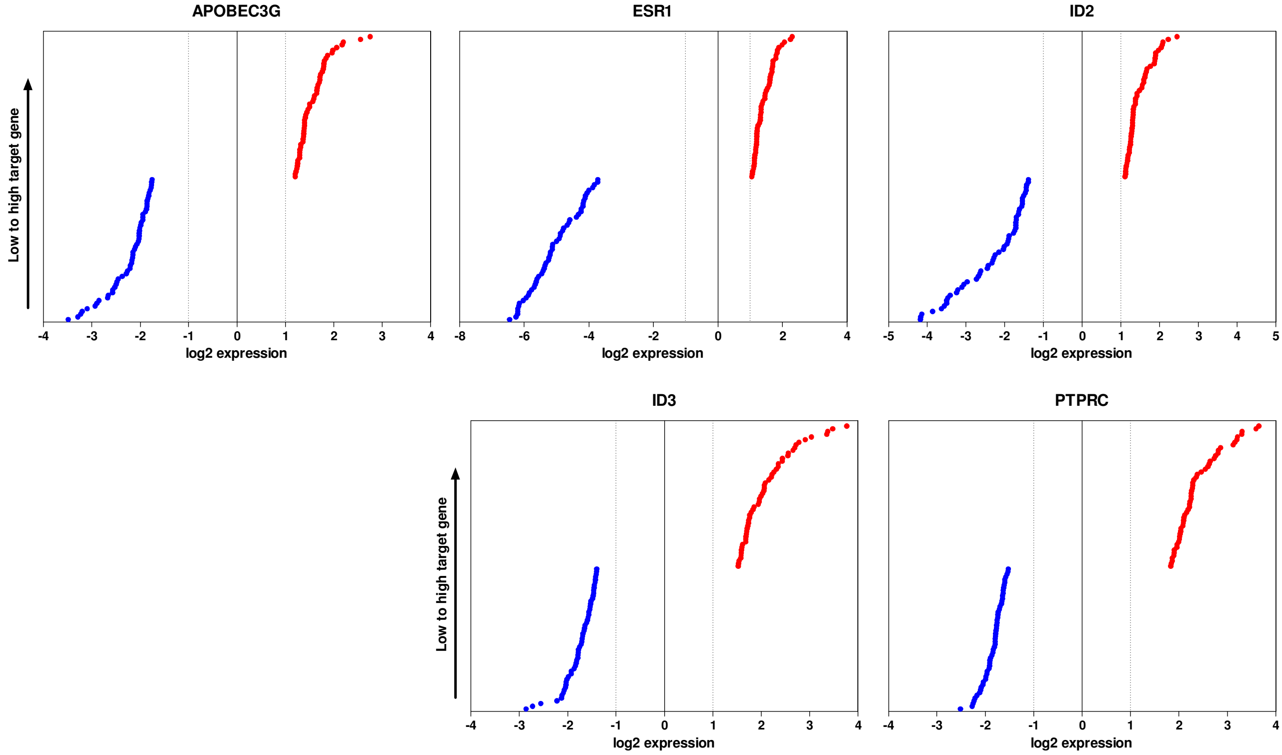

Figure S1 Graphical view of the expression range of target genes from ovarian cancer samples used in GENEVESTIGATOR-based analysis. Signal values from 536 ovarian cancer samples were extracted from public microarrays data sets using GENEVESTIGATOR and samples with profound difference to the median target gene expression value were used for further analysis. The 10th and 90th percentiles were defined as cutpoints to designate the target gene^{low} and the target gene^{high} microarray data sets, respectively; the color code: blue dots, ≤10th percentile; red dots, ≥90th percentile. Signal values were log2-transformed and are shown relative to the median expression intensity. Additional gridlines (light grey) highlight the 2-fold differences in expression intensities.

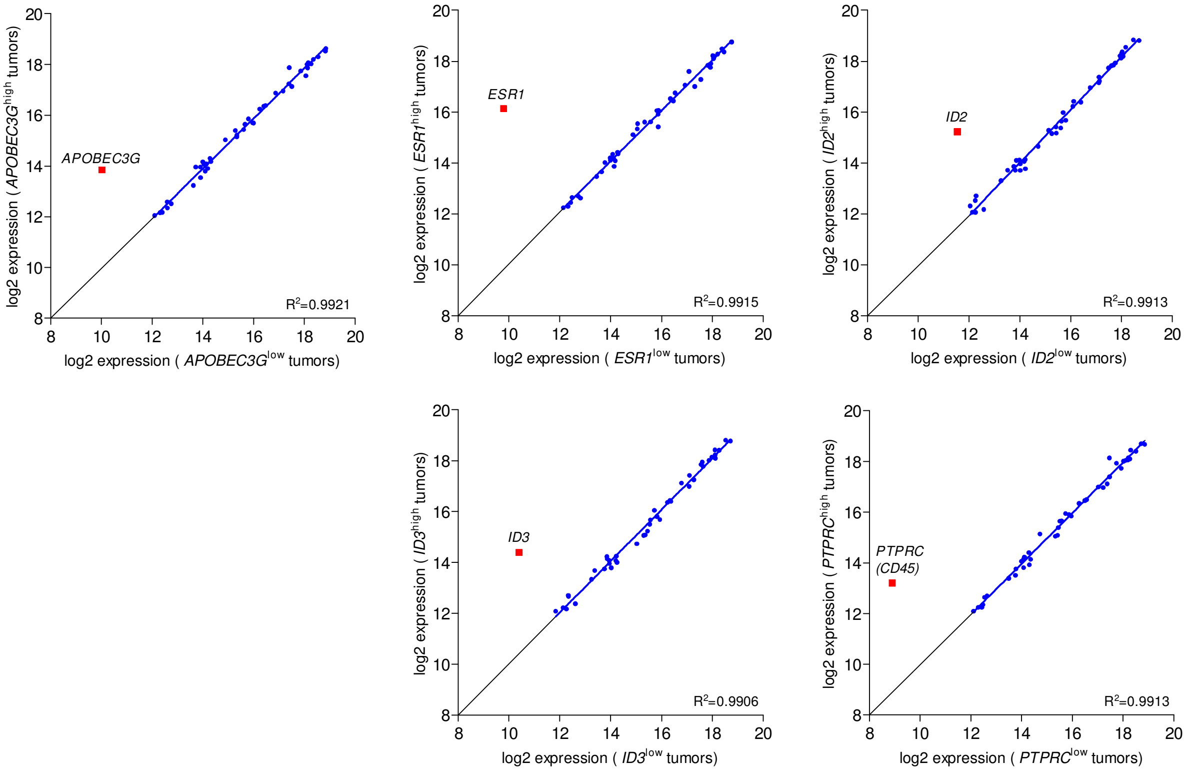

Figure S2 Correlation analysis of reference HKGs expression values in the target gene^{high}/90th percentile and the target gene^{low}/10th percentile microarray data sets. To assess homogeneity between the target gene^{high} (n=53) and the target gene^{low} (n=53) groups of tumor samples, the Pearson correlation analysis across a panel of reference HKGs was done using GraphPad Prism 5 software. The log2 transformed mean expression value of each target gene depicted as red square in the scatter plot is shown in relation to the log2 transformed data points for reference HKGs (n=45, blue dots). The correlation analysis between the target gene^{high} and low expressing tumors revealed high homogeneity across a panel of reference HKGs (R2>0.99) for each target gene, as expected, thus indicating that the changes in target gene expression are likely to be caused by intrinsic gene regulation and not by external influences, e.g. sample quality and that those data sets cannot be considered as target gene expression outliers.

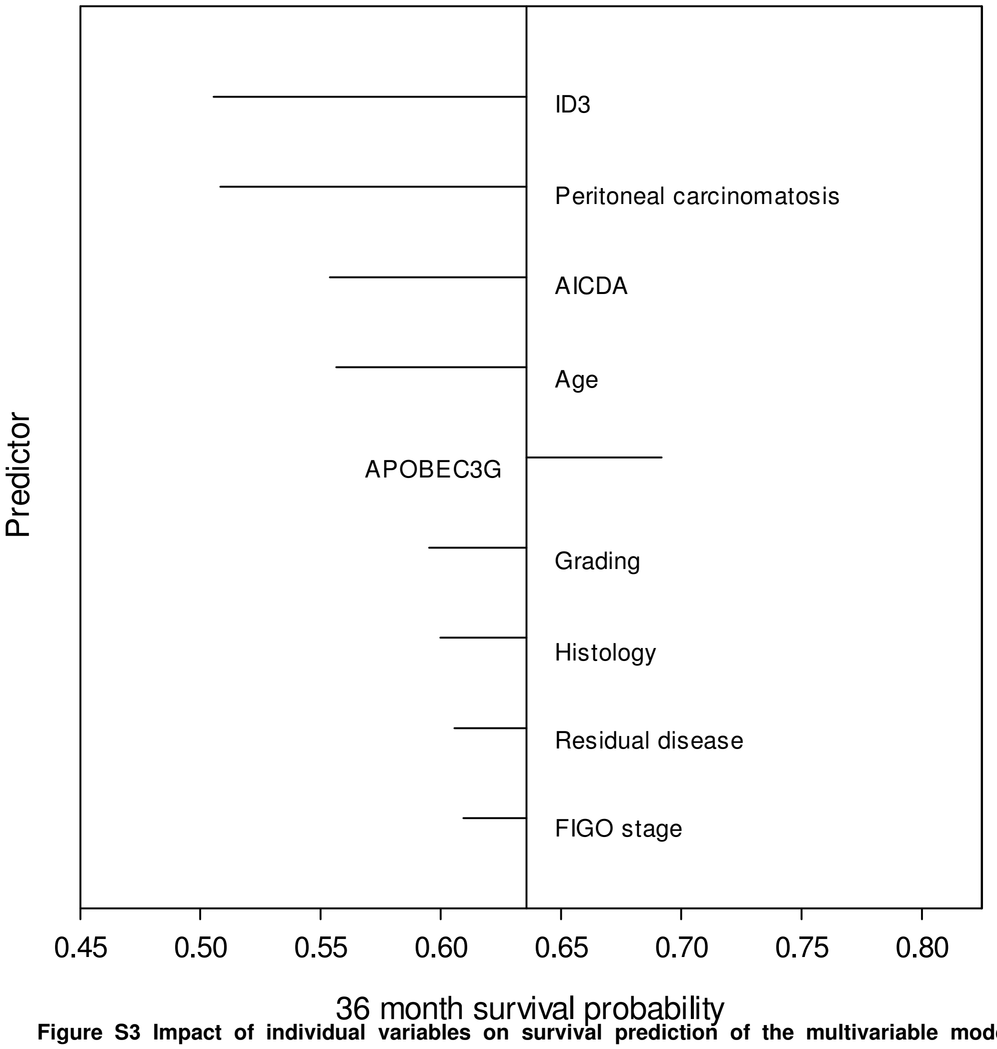

Figure S3 Impact of individual variables on survival prediction of the multivariable model (LASSO) for OS if predictors are changed by +1 SD. Survival probabilities are estimated at 36 months of follow up time. The length of the lines is proportional to the change in prediction in case the predictor of the indicated variable changes by +1 SD (left: negative effect; right: positive effect). Variables are ranked according to the absolute values of change in prediction and correspond to the order within the combined model (Supplementary Table S8).

Overall survival

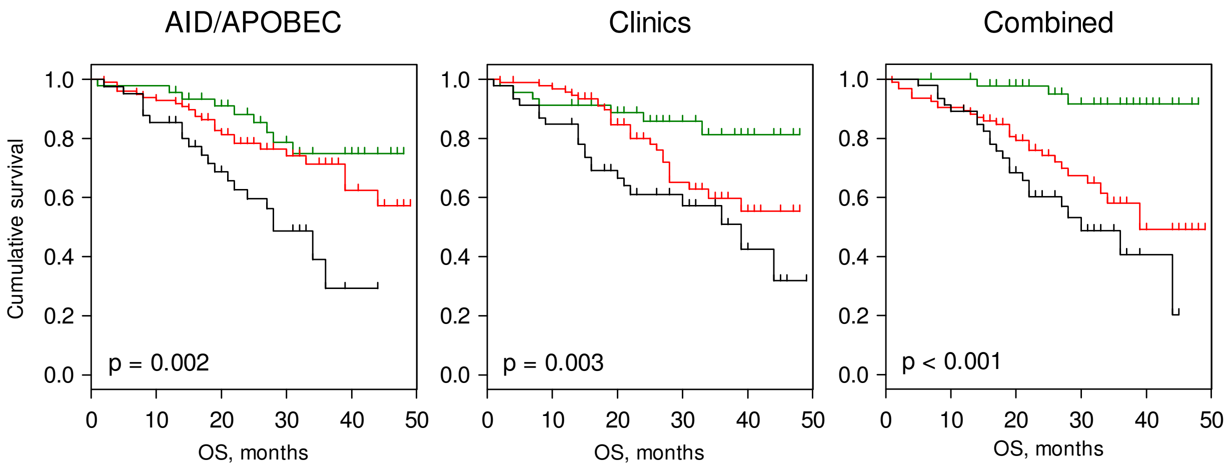

Progression-free survival

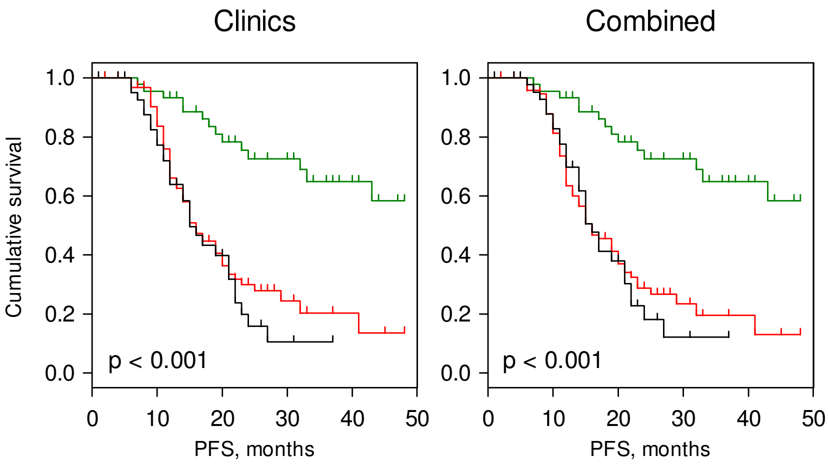

Figure S4 Kaplan-Meier estimates for patient stratification based on the AID/APOBEC model, the clinical model, and the combined one (LASSO-based). Kaplan-Meier curves for OS and PFS are shown giving patients' stratification into low risk (n=46, green), intermediate risk (n=94, red), and high risk (n=46, black) groups with the 25th and 75th percentiles serving as thresholds (lower than the 25th percentile indicates low risk). No stable and well calibrated model for AID/APOBEC was found in respect of PFS, thus only models for Clinics and Combined are shown. P-value of the log-rank test is indicated.

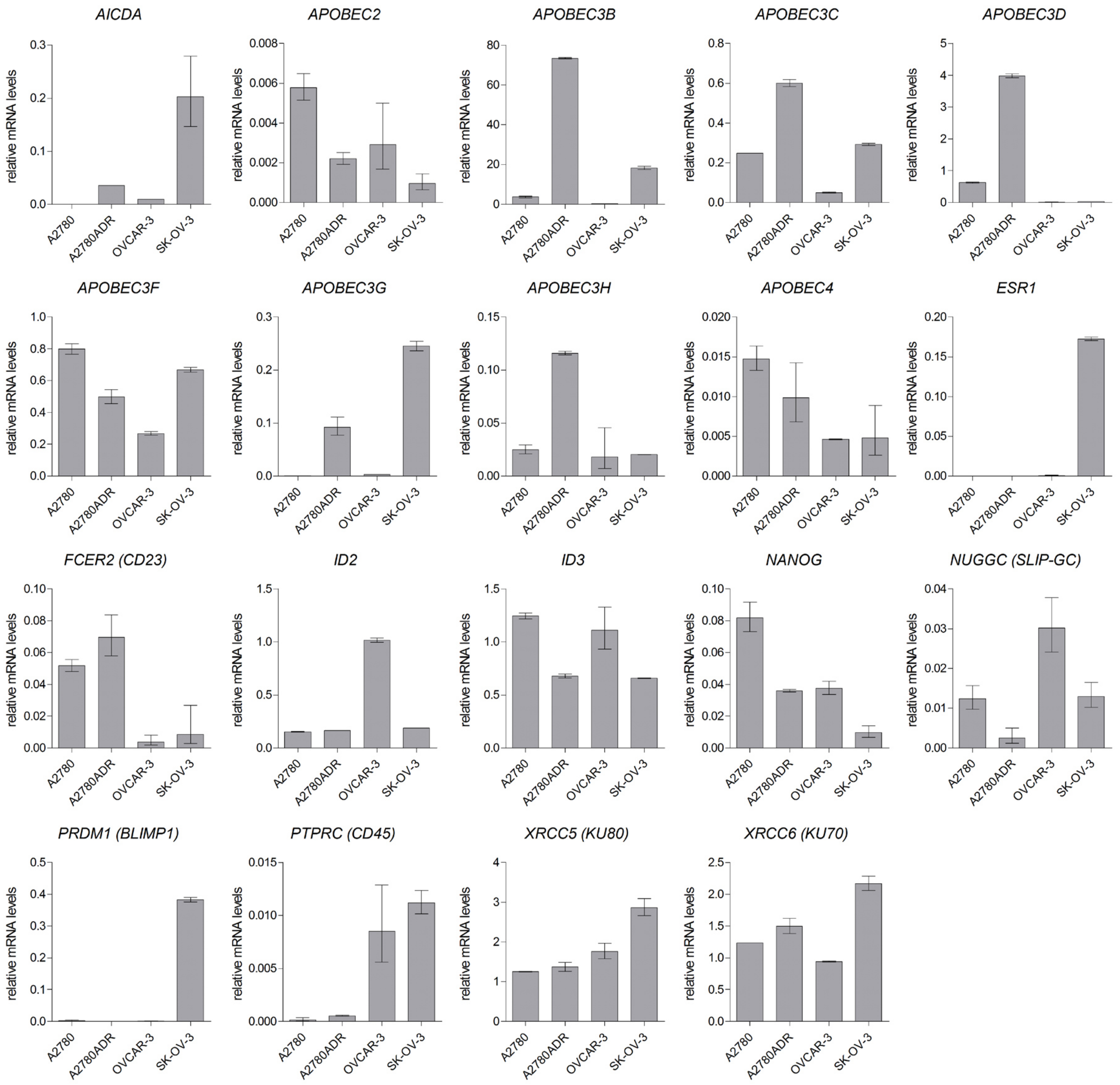

Figure S5 Expression profiles of genes from the AID/APOBEC-based multigene signature in ovarian cancer cell lines. Real-time PCR analysis was performed using the ΔΔCt method for relative quantification. Expression mRNA levels of the target gene were normalized to the average of the HKGs and calculated relative to the corresponding mRNA levels within qPCR human reference total RNA (equated to 1.0). Genes such as APOBEC1, APOBEC3A, ESR2, and PAX5 exhibiting expression at or below the detection limit are not shown. In respect of the APOBEC3 subfamily, A3B/C/D/F/H mRNAs were detected in all examined cell lines; APOBEC3G mRNA was detected in SK-OV-3 and the drug resistant A2780ADR. AID mRNA expression was detected in SK-OV-3 and A2780ADR. Comparison of expression profiles of the parental A2780 cell line and the corresponding drug resistant A2780ADR cell line revealed enhanced mRNA expression levels for the majority of the APOBEC3 subfamily members in A2780ADR cells. Results are shown as mean values in one experiment ± SD and are representative of two independent experiments. An interesting finding of the current study, although beyond the major focus, is the detection of enhanced mRNA expression levels for the majority of the APOBEC3 subfamily members in the ovarian cancer cell line A2780ADR (resistant to adriamycin/doxorubicin; collateral resistance to cisplatin) in comparison to the parental A2780 cells (drug-sensitive; high chemosensitivity to cisplatin). The knowledge collected through HIV-1 and APOBEC3G interrelation analysis suggests that the low levels of APOBEC3G activity may promote infrequent mutations and lead to beneficial viral variants resistant to anti-HIV-1 treatment regimen [1]. Considering the potential analogy in APOBEC3-mediated editing of genomic DNA, APOBEC3 subfamily members, particularly APOBEC3B, which was already shown to contribute to the mutational pattern of ovarian cancer cells [2], may facilitate the drug-resistant adaptation. This novel data-driven hypothesis regarding the complex ways underlying the acquisition of drug resistance needs further experimentations with a wider range of drug-resistant cell lines.

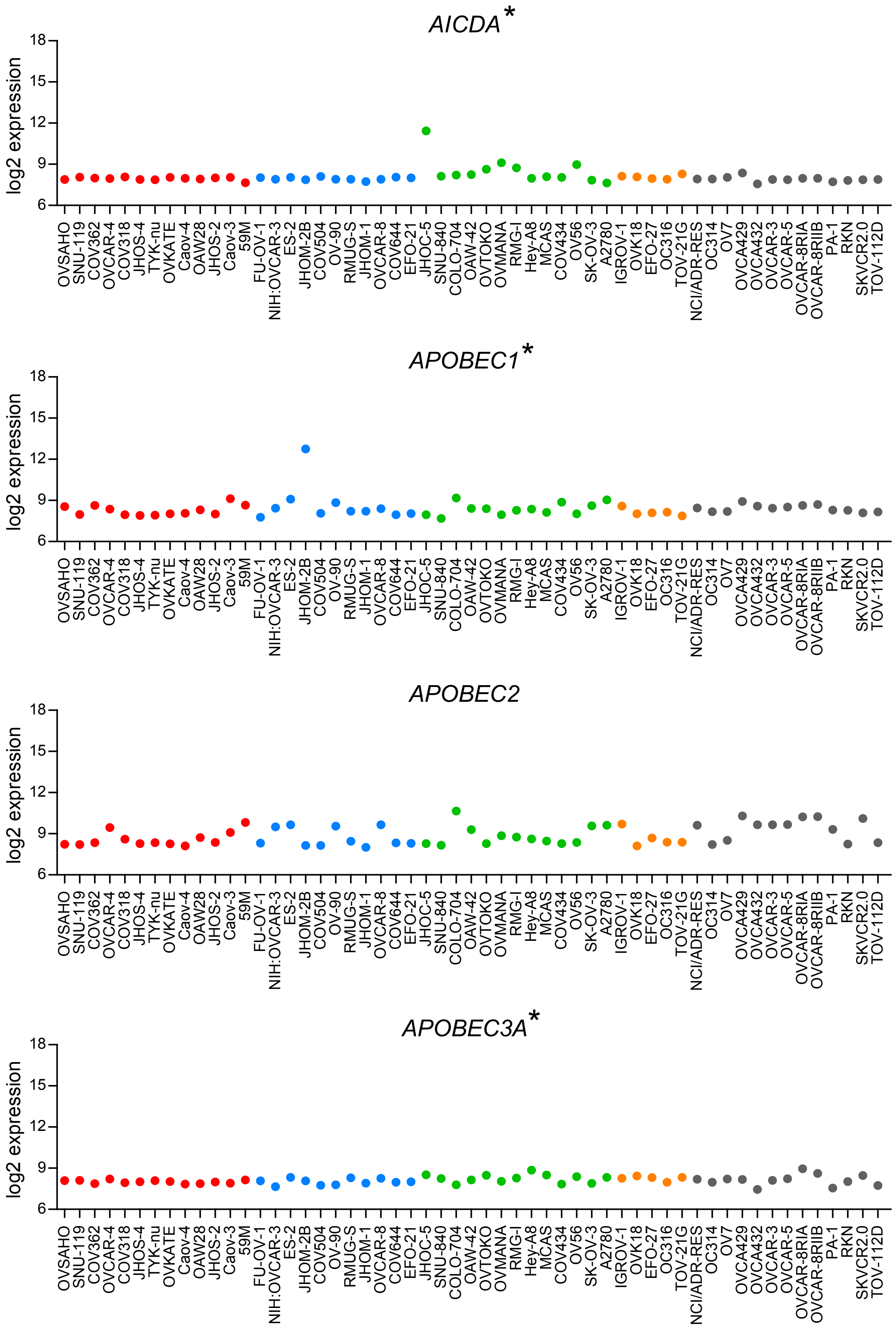

Figure S6 part 1

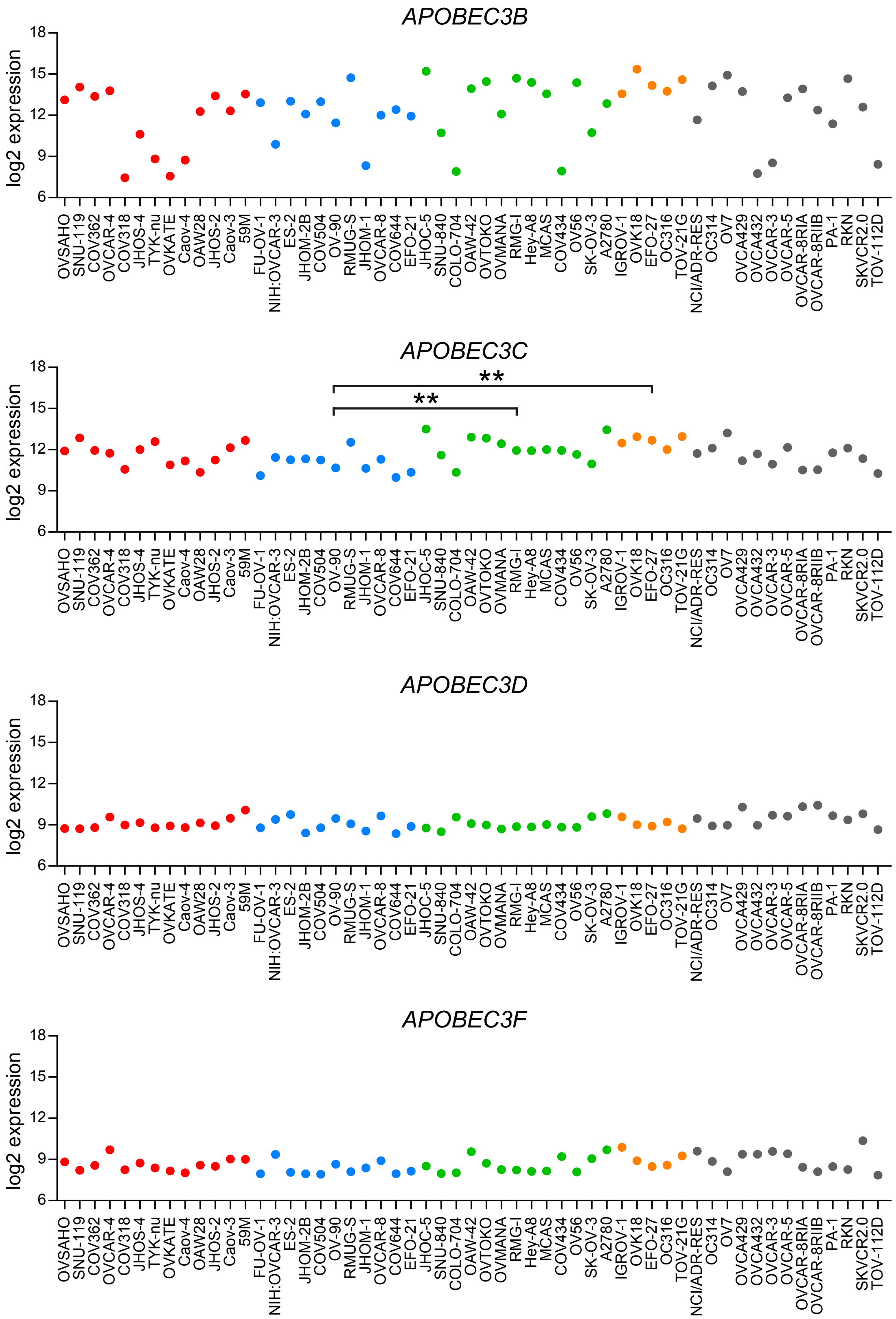

Figure S6 part 2

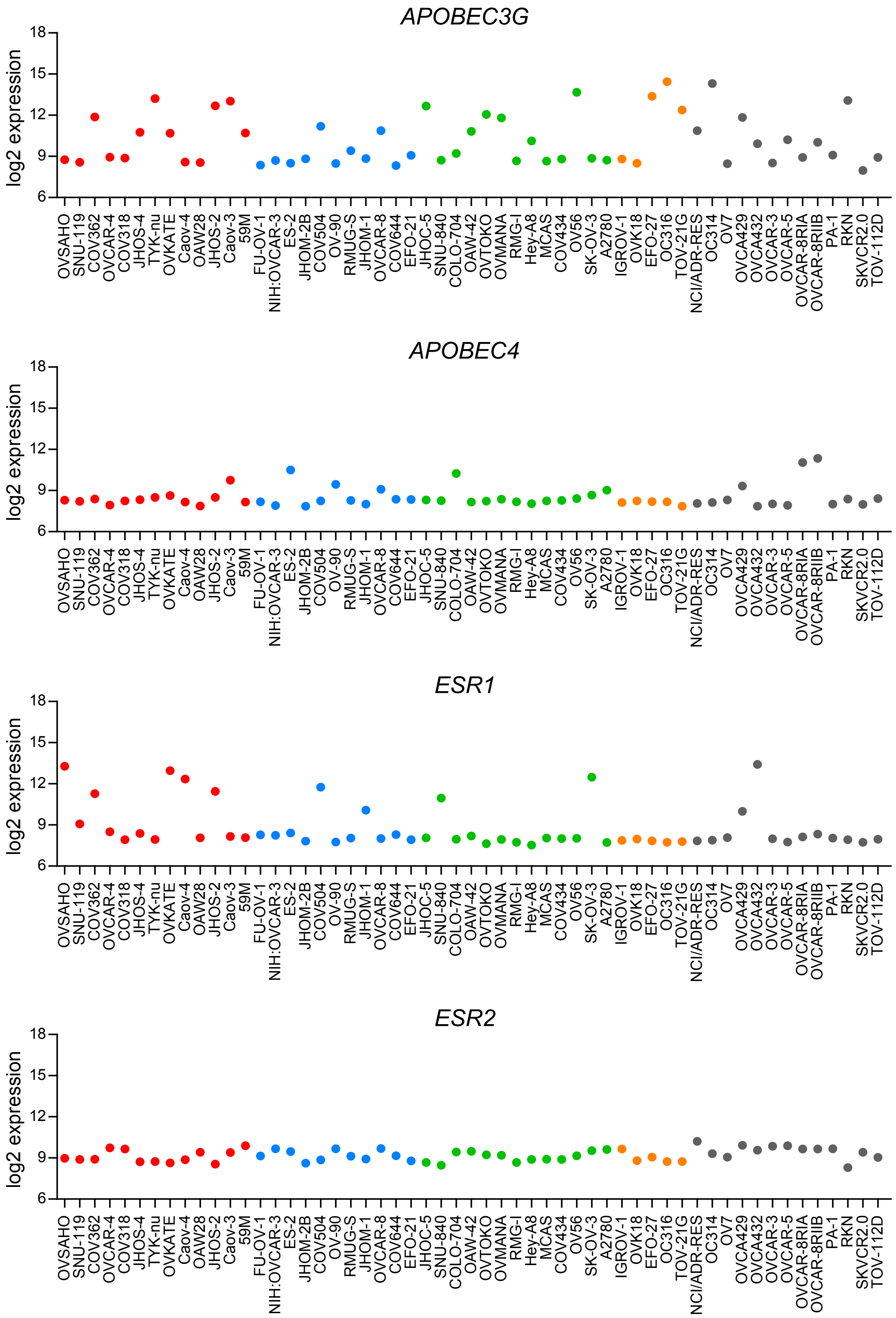

Figure S6 part 3

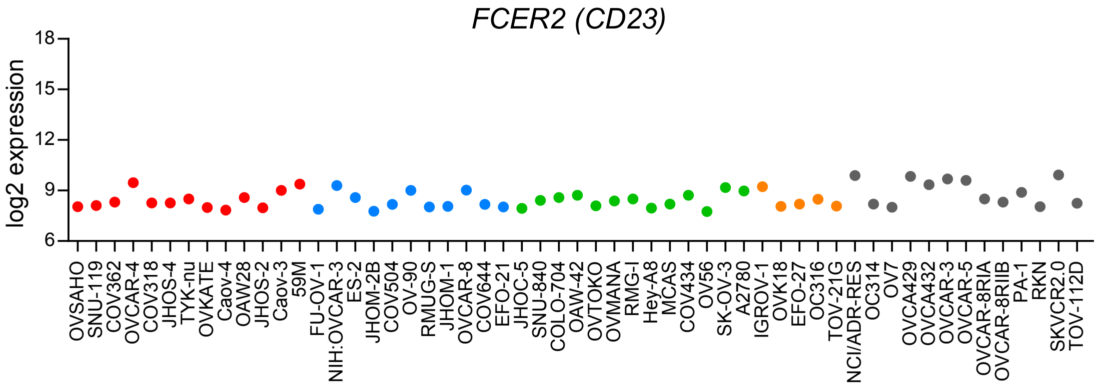

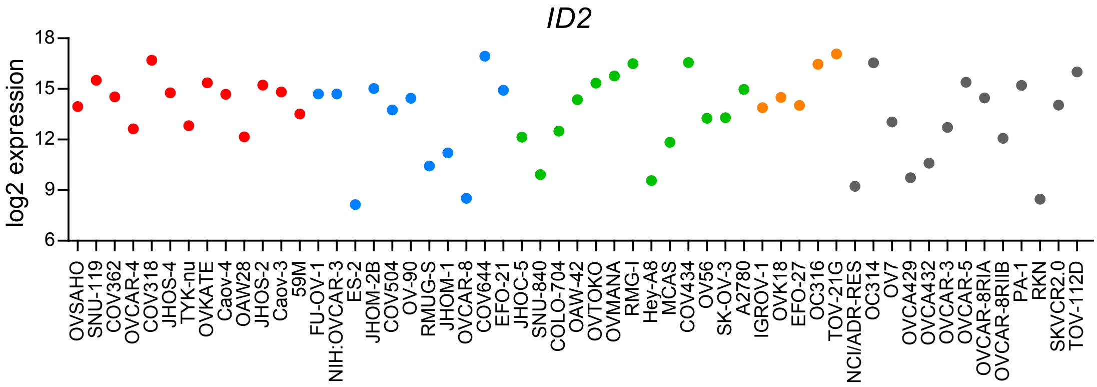

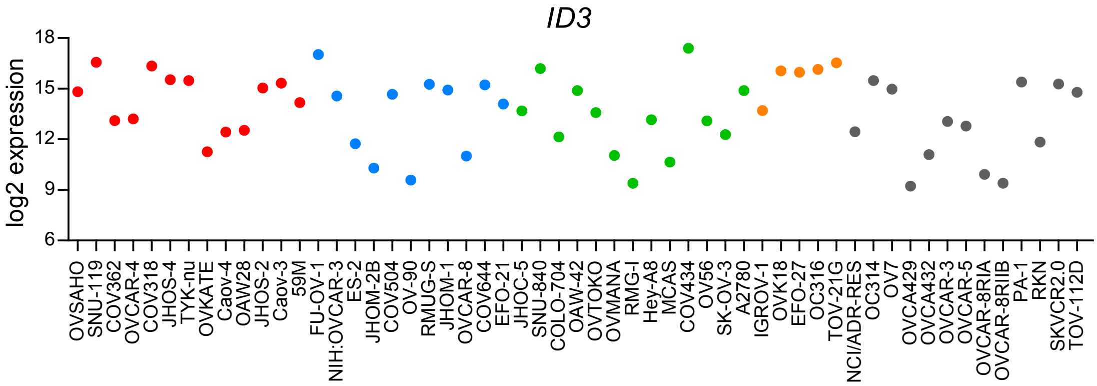

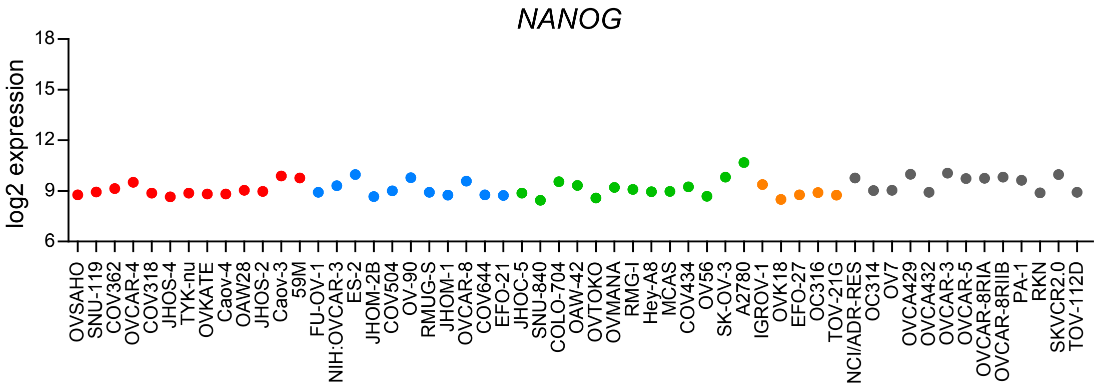

Figure S6 part 4

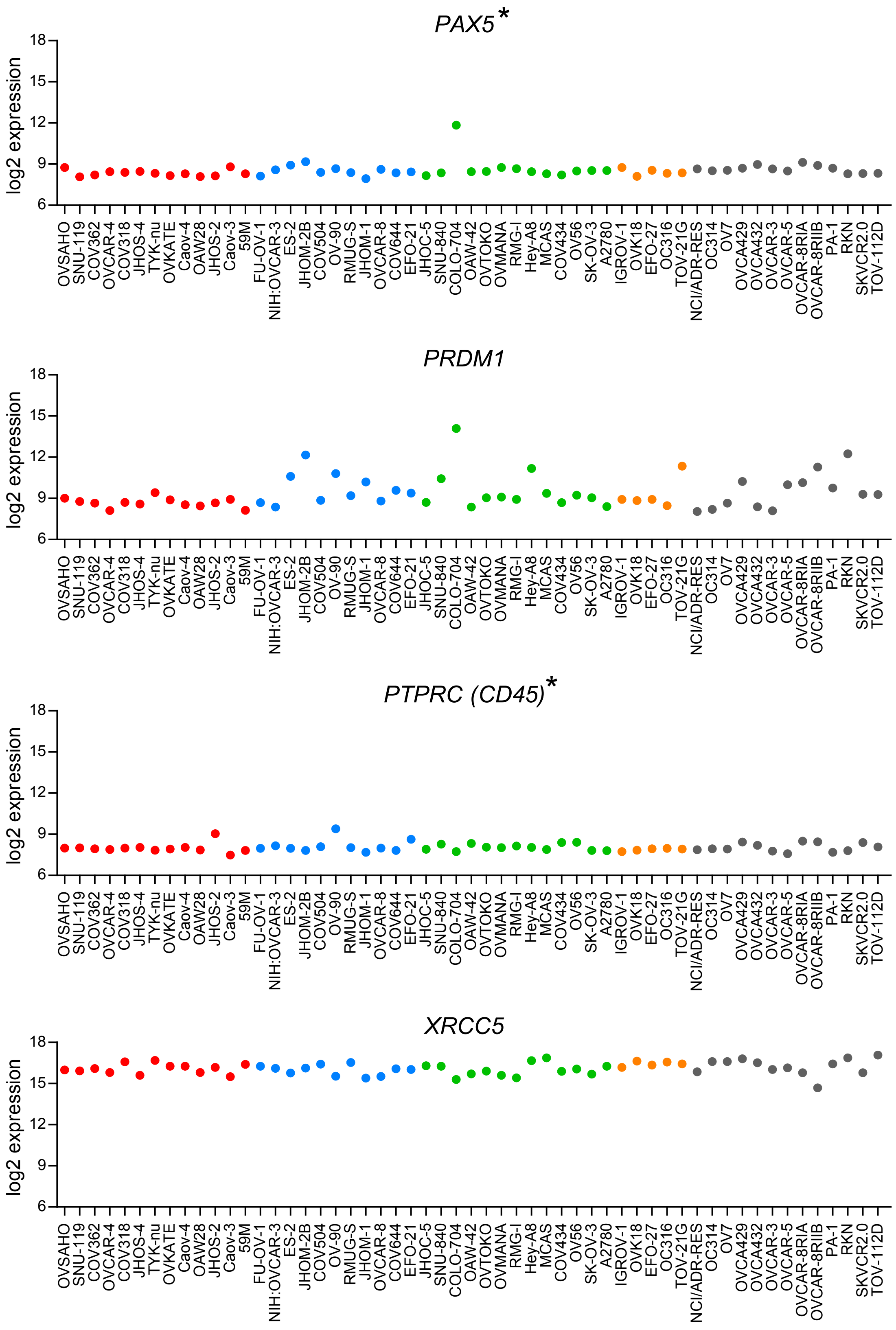

Figure S6 part 5

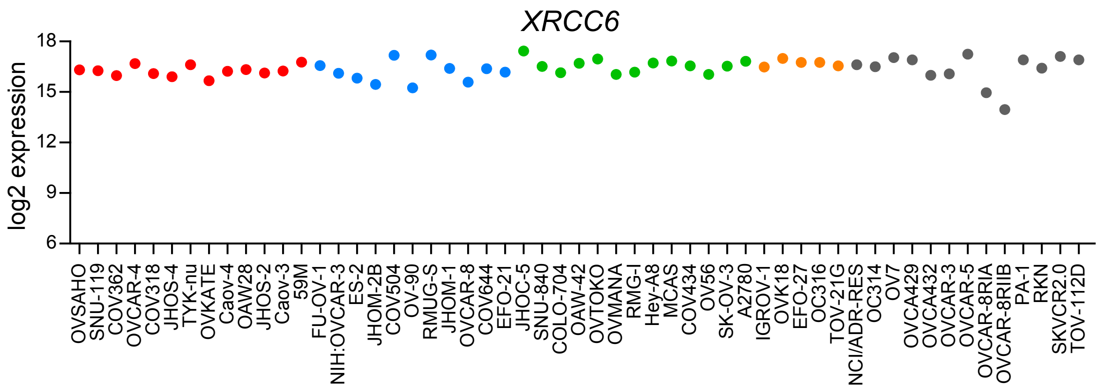

Figure S6 Expression profiles of genes from the AID/APOBEC-based multigene signature in a wide range of ovarian cancer cell lines across previously published microarray data sets. Expression values (log2 transformed) for individual genes across 55 ovarian cancer cell lines were extracted using GENEVESTIGATOR. Cell lines were grouped into sub-categories according to Domcke et al. Color code: *red*, likely high-grade serous; *blue*, possibly high-grade serous; *green*, unlikely high-grade serous; *orange*, hyper-mutated; *grey*, unclassified. Genes exhibiting low expression or expression at microarray detection limit are indicated by asterisks. Only statistically significant differences between the groups assessed by two-way analysis of variance (ANOVA) are indicated; ** p<0.01.

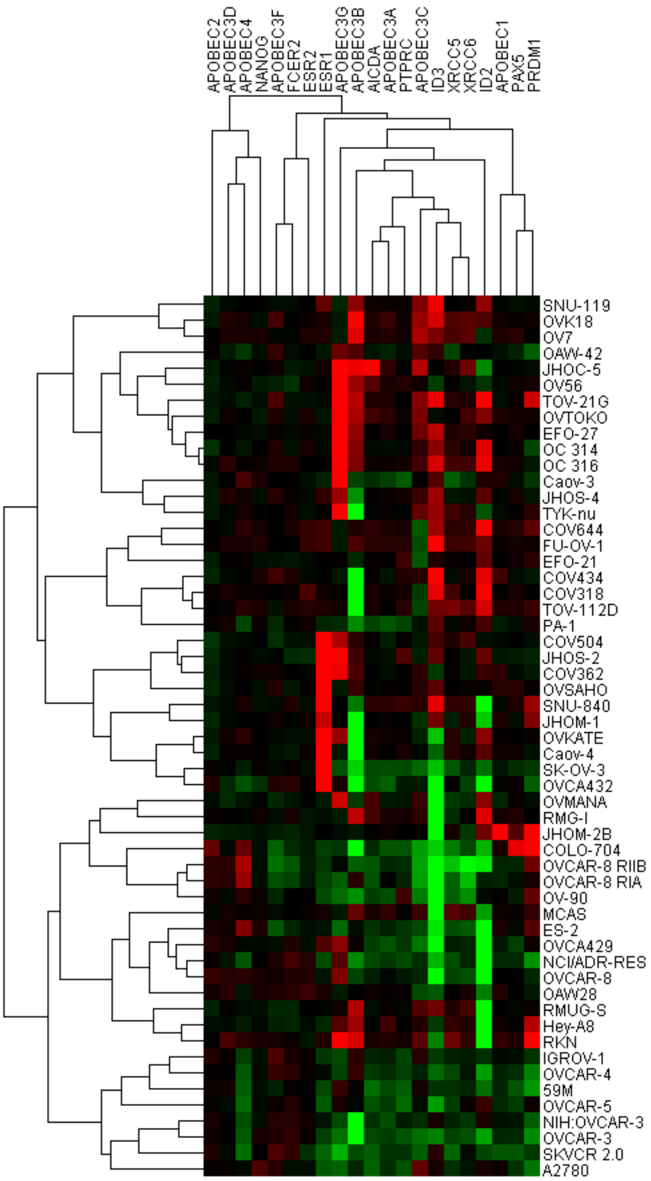

Figure S7 Hierarchical clustering of expression data sets. Pearson uncentered hierarchical clustering was applied on log2 transformed expression values for individual genes composing the AID/APOBEC multigene signature across arrays/samples of 55 ovarian cancer cell lines. Shown is the result of a clustering run representing a pair of trees, one for genes and one for arrays/samples (Cluster/TreeView programs). Color code: *red* represents higher expression and *green* indicates lower expression.

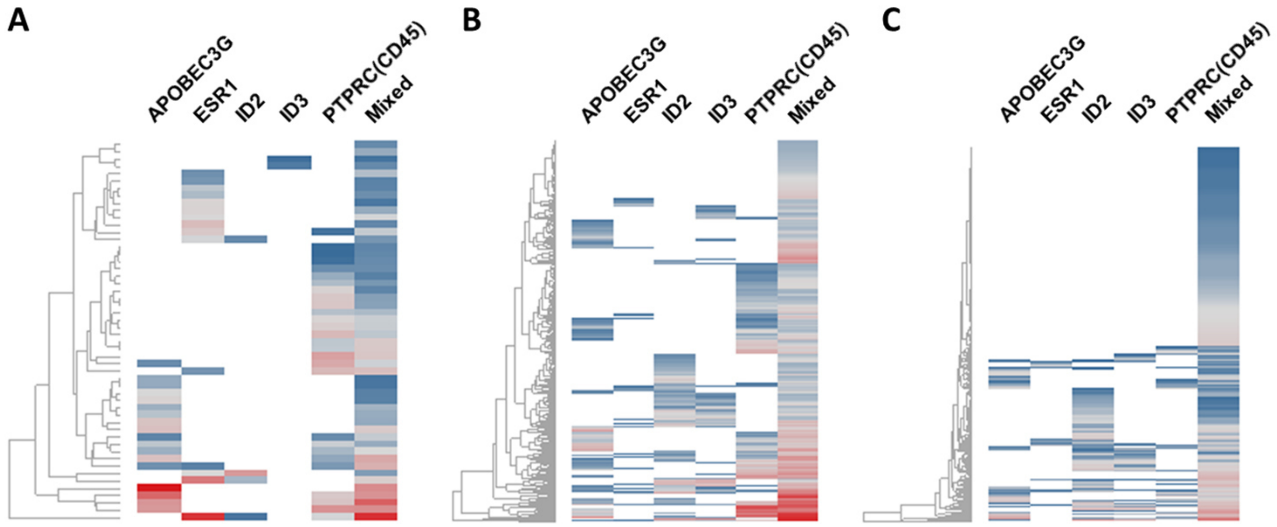

Figure S8 The heat map of (A) Canonical Pathways, (B) Functional Annotations, and (C) Upstream Regulators. The row dendrogram indicates the hierarchical clustering of the IPA-derived pathways from mixed and individual outputs; in matrix representation is followed by hierarchical clustering using Euclidean distance measure. Color code for the corresponding –log10(p-value): red, max; grey, average; blue, min. Blanks indicate that the corresponding Canonical Pathway/Functional Annotation/Upstream Regulator was not found as significant in output_individual.

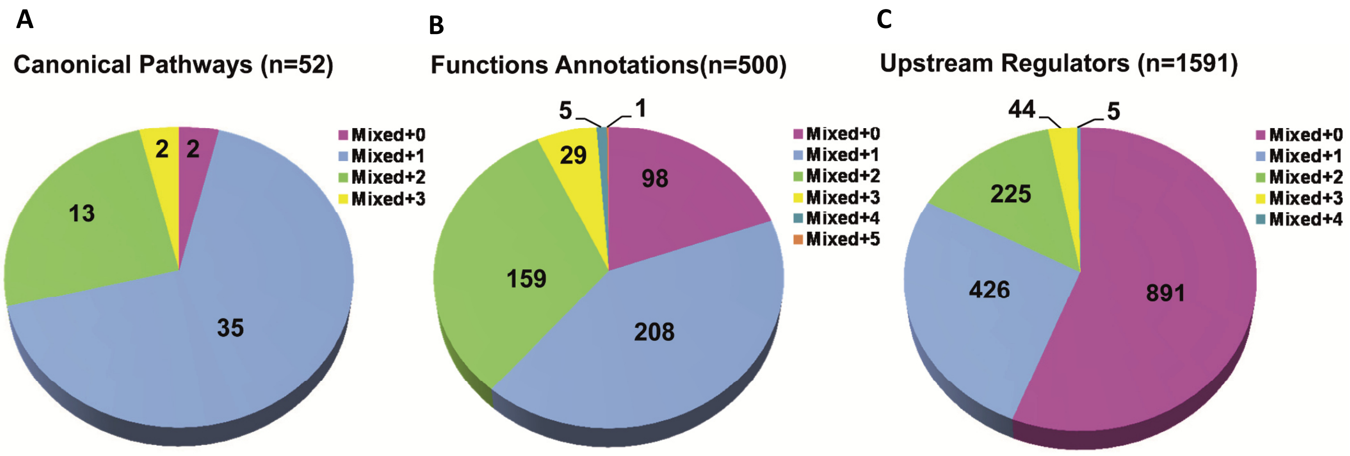

Figure S9 Analysis of the number of (A) Canonical Pathways, (B) Functional Annotations, and (C) Upstream Regulators overlapping between output_mixed and output_individual. Count of Canonical Pathways/Functional Annotations/Upstream Regulators present in a given category of the overlap (output_overlap) is shown by pie chart. Each category is characterized by the mandatory presence in output_mixed and in indicated number of the output_individual (e.g. mixed+0, the presence in output_mixed only; mixed+1; the presence in output_mixed and in any one of the output_individual). (A) Out of 52 pathways (i) 2 are assigned to mixed only, (ii) 35 are found in mixed and any one individual, (iii) 13 are found in mixed and any two individual, (iv) 2 are found in mixed and any three individual, (v) none is found in mixed and any four or all five individual target genes. (B) 98 out of 500 Functional Annotations were assigned to the mixed only; furthermore, the strongest overlaps were found for mixed and any one (208/500) and two (159/500) individual. (C) The major contribution to the over-represented Upstream Regulators is given by the outcome_mixed (891/1591); none is assigned to mixed and all five individual.

Table S1 Real-time PCR primer sequences.

Gene symbol	Synonym	Accession number NCBI	Sequences of primers	
			Forward	Reverse
AICDA	*AID*	NM_020661	GGACTTTGGTTATCTTCGCAATAAG	GTCGGGCACAGTCGTAGCA
APOBEC1	*BEDP*	NM_001644.3	AACAAAATCGGCAAGGTCTCA	GCAGTGATAATACTCTGATGCTCTCAT
APOBEC2	*ARP1*	NM_006789.3	GAAGTAGGGCAACTGGGCTTT	TGTCCCAGATGGCTGTACATG
APOBEC3A	*ARP3*	NM_145699.3	AGAAGGGACAAGCACATGGAA	AAGTGAATATGTGTGGATCCATCAA
APOBEC3B	*ARP4*	NM_001270411.1	GCCTTGGTACAAATTCGATGAAA	GAAAGTGAATGTGTCTGGATCCAT
APOBEC3C	*ARP5*	NM_014508	GCATATCTAAGAGGCTGAACATGAAT	TGGAAGTAGAATGTGCCTGGATAC
APOBEC3D	*ARP6*	NM_152426.3	ACTACCCAAACGTCAGTCGAATC	GCAGTCGGTTGCCACAGAA
APOBEC3F	*ARP8*	NM_145298.5	GGAGGGCTGTCCTGAAACCT	AAAAGTTGTAGGAGAATGTGTCTCGAT
APOBEC3G	*ARP9*	NM_021822	GTGGAGCGCATGCACAATG	GGCCTTCAAGGAAACCGTGT
APOBEC3H	*ARP10*	NM_001166003.1	CAGCTGACGCCGCAGAAT	GACTTGATCTCGTTAATAAAGCAAATTTC
APOBEC4	*C1orf169*	NM_203454.2	AATTGATGGTTTGCAGCTAGAAGA	CCATGATTTGCTAGGTACTCCTCAT
DPPA3	*STELLA*	NM_199286	GCGGAGTTCGTACGCATGA	CGCAGAAACTGCAGGGACAT
EEF1A1	*EF-Tu*	NM_001402.5	ATTACAGGGACATCTCAGGCTGAC	CATTCTTGGAGATACCAGCTTCAA
FCER2	*CD23*	NM_002002	AGGTGTCCAGCGGCTTTGT	AGCACTTCCGTTGGAAATTGA
ID2	*GIG8*	NM_002166	TGTGGCTGAATAAGCGGTGTT	TCAGCACTTAAAAGATTCCGTGAA
ID3	*HEIR-1*	NM_002167	GCTCACTCCGGAACTTGTCATC	CCAGCACCTGCGTTCTGGA
NANOG		NM_024865	ATGCCTCACACGGAGACTGTCT	TGACCGGGACCTTGTCTTCC
NUGGC	*SLIP-GC*	NM_001010906	GGGCAGCATCACCACTATGCT	TTCACTCCCGACATCTGCAA
PAX5	*BSAP*	NM_016734	CGTACAACGACTCCTGGAGGTTC	GGCGGCAGCGCTATAATAGTA
PRDM1	*BLIMP1*	NM_001198	CGGAGAGCTGACAATGATGAATC	GGGACATTCTTTGGGCAGAGT
PTPRC	*CD45*	NM_002838	CCCTCAAAGATCATTTCATAATTTTACC	GTAGGCATGTAATGATAAAACATATTTCG
XRCC5	*KU80*	NM_021141	CTCCCACCGAGGCACAGT	TTTGGTGGTTGGAAACAAGTCTT
XRCC6	*KU70*	NM_001469	CAATCCTGAAGGGAAAGTTACCA	GCTGATGTGGGTCTTCAGCTC

Table S2 Real-time PCR primers [A].

Gene symbol	Synonym	Accession number NCBI	Supplier	Assay ID
ACTB		NM_001101.2	Applied Biosystems	4326315E
ESR1	*ESRA*	NM_000125.3	Applied Biosystems	Hs00174860_m1
ESR2	*ESRB*	NM_001040275.1	Applied Biosystems	Hs00230957_m1
TOP1	*HEIR-1*	NM_003286.2	PrimerDesign Ltd	HK-DD-300 (TOP1)
UBC	*HMG-20*	NM_021009.5	PrimerDesign Ltd	HK-DD-300 (UBC)
YWHAZ	*14-3-3-zeta*	NM_003406.3	PrimerDesign Ltd	HK-DD-300 (YWHAZ)

[A] Primer sequences are proprietary information of the manufacturer

Table S3 Genes composing the AID/APOBEC multigene signature. Gene symbols, synonyms, full names, accession numbers and a short description from NCBI are provided.

Symbol	Synonym	Name	NCBI accession number	Short functional description
**AICDA**	**AID**	activation-induced cytidine deaminase	NM_020661.2	This gene encodes a RNA-editing deaminase that is a member of the cytidine deaminase family. The protein is involved in somatic hypermutation, gene conversion, and class-switch recombination of immunoglobulin genes. Defects in this gene are the cause of autosomal recessive hyper-IgM immunodeficiency syndrome type 2 (HIGM2). [provided by NCBI RefSeq, Feb 2009]
**APOBEC1**	**BEDP**	apolipoprotein B mRNA editing enzyme, catalytic polypeptide 1	NM_001644.3	This gene encodes a member of the cytidine deaminase enzyme family. The encoded protein forms a multiple-protein editing holoenzyme with APOBEC1 complementation factor (ACF) and APOBEC1 stimulating protein (ASP). This holoenzyme is involved in the editing of C-to-U nucleotide bases in apolipoprotein B and neurofibromatosis-1 mRNAs. [provided by NCBI RefSeq, Jul 2008]
**APOBEC2**	**ARP1**	apolipoprotein B mRNA editing enzyme, catalytic polypeptide-like 2	NM_006789.3	APOBEC2 (apolipoprotein B mRNA editing enzyme, catalytic polypeptide-like 2) is a protein-coding gene. Diseases associated with APOBEC2 include tonsillitis, and thyroiditis. GO annotations related to this gene include cytidine deaminase activity and RNA binding. An important paralog of this gene is APOBEC3G. [provided by www.genecards.org]
**APOBEC3A**	**ARP3**	apolipoprotein B mRNA editing enzyme, catalytic polypeptide-like 3A	NM_001270406.1; NM_145699.3	This gene is a member of the cytidine deaminase gene family. It is one of seven related genes or pseudogenes found in a cluster, thought to result from gene duplication, on chromosome 22. Members of the cluster encode proteins that are structurally and functionally related to the C to U RNA-editing cytidine deaminase APOBEC1. The protein encoded by this gene lacks the zinc binding activity of other family members. The protein plays a role in immunity, by restricting transmission of foreign DNA such as viruses. One mechanism of foreign DNA restriction is deamination of foreign double-stranded DNA cytidines to uridines, which leads to DNA degradation. However, other mechanisms are also thought to be involved, as anti-viral effect is not dependent on deaminase activity. Two transcript variants encoding different isoforms have been found for this gene. [provided by NCBI RefSeq, Jul 2012]
**APOBEC3B**	**ARP4**	apolipoprotein B mRNA editing enzyme, catalytic polypeptide-like 3B	NM_001270411.1	This gene is a member of the cytidine deaminase gene family. It is one of seven related genes or pseudogenes found in a cluster, thought to result from gene duplication, on chromosome 22. Members of the cluster encode proteins that are structurally and functionally related to the C to U RNA-editing cytidine deaminase APOBEC1. It is thought that the proteins may be RNA editing enzymes and have roles in growth or cell cycle control. A hybrid gene results from the deletion of approximately 29.5 kb of sequence between this gene, APOBEC3B, and the adjacent gene APOBEC3A. The breakpoints of the deletion are within the two genes, so the deletion allele is predicted to have the promoter and coding region of APOBEC3A, but the 3' UTR of APOBEC3B. Two transcript variants encoding different isoforms have been found for this gene. [provided by NCBI RefSeq, Jul 2012]
**APOBEC3C**	**ARP5**	apolipoprotein B mRNA editing enzyme, catalytic polypeptide-like 3C	NM_014508	This gene is a member of the cytidine deaminase gene family. It is one of seven related genes or pseudogenes found in a cluster thought to result from gene duplication, on chromosome 22. Members of the cluster encode proteins that are structurally and functionally related to the C to U RNA-editing cytidine deaminase APOBEC1. It is thought that the proteins may be RNA editing enzymes and have roles in growth or cell cycle control. [provided by NCBI RefSeq, Jul 2008]
**APOBEC3D**	**A3D; ARP6; APOBEC3E**	apolipoprotein B mRNA editing enzyme, catalytic polypeptide-like 3D	NM_152426.3	This gene is a member of the cytidine deaminase gene family. It is one of a group of related genes found in a cluster, thought to result from gene duplication, on chromosome 22. Members of the cluster encode proteins that are structurally and functionally related to the C to U RNA-editing cytidine deaminase APOBEC1 and inhibit retroviruses, such as HIV, by deaminating cytosine residues in nascent retroviral cDNA. [provided by NCBI RefSeq, Jul 2008]
**APOBEC3F**	**ARP8**	apolipoprotein B mRNA editing enzyme, catalytic polypeptide-like 3F		This gene is a member of the cytidine deaminase gene family. It is one of seven related genes or pseudogenes found in a cluster, thought to result from gene duplication, on chromosome 22. Members of the cluster encode proteins that are structurally and functionally related to the C to U RNA-editing cytidine deaminase APOBEC1. It is thought that the proteins may be RNA editing enzymes and have roles in growth or cell cycle control. Alternatively spliced transcript variants encoding different isoforms have been identified. [provided by NCBI RefSeq, Jul 2008]

Table S3 (continued) Genes composing the AID/APOBEC multigene signature. Gene symbols, synonyms, full names, accession numbers and a short description from NCBI are provided.

Symbol	Synonym	Name	NCBI accession number	Short functional description
**APOBEC3G**	**ARP9; CEM15**	apolipoprotein B mRNA editing enzyme, catalytic polypeptide-like 3G	NM_021822	This gene is a member of the cytidine deaminase gene family. It is one of seven related genes or pseudogenes found in a cluster, thought to result from gene duplication, on chromosome 22. Members of the cluster encode proteins that are structurally and functionally related to the C to U RNA-editing cytidine deaminase APOBEC1. It is thought that the proteins may be RNA editing enzymes and have roles in growth or cell cycle control. The protein encoded by this gene has been found to be a specific inhibitor of human immunodeficiency virus-1 (HIV-1) infectivity. [provided by NCBI RefSeq, Jul 2008]
**APOBEC3H**	**ARP10**	apolipoprotein B mRNA editing enzyme, catalytic polypeptide-like 3H	NM_001166002.1; NM_001166003.1; NM_001166004.1; NM_181773.3	This gene encodes a member of the apolipoprotein B mRNA-editing enzyme catalytic polypeptide 3 family of proteins. The encoded protein is a cytidine deaminase that has antiretroviral activity by generating lethal hypermutations in viral genomes. Polymorphisms and alternative splicing in this gene influence its antiretroviral activity and are associated with increased resistence to human immunodeficiency virus type 1 infection in certain populations. Alternative splicing results in multiple transcript variants.[provided by NCBI RefSeq, Oct 2009]
**APOBEC4**	**C1orf169**	apolipoprotein B mRNA editing enzyme, catalytic polypeptide-like 4 (putative)	NM_203454.2	This gene encodes a member of the AID/APOBEC family of polynucleotide (deoxy)cytidine deaminases, which convert cytidine to uridine. Other AID/APOBEC family members are involved in mRNA editing, somatic hypermutation and recombination of immunoglobulin genes, and innate immunity to retroviral infection. [provided by NCBI RefSeq, Jul 2008]
**DPPA3**	**STELLA**	developmental pluripotency associated 3	NM_199286	This gene encodes a protein that in mice may function as a maternal factor during the preimplantation stage of development. In mice, this gene may play a role in transcriptional repression, cell division, and maintenance of cell pluripotentiality. In humans, related intronless loci are located on chromosomes 14 and X. [provided by NCBI RefSeq, Jul 2008]
**ESR1**	**ER; ESR; Era; ESRA; NR3A1**	estrogen receptor 1	NM_000125.3; NM_001122740.1; NM_001122741.1; NM_001122742.1	This gene encodes an estrogen receptor, a ligand-activated transcription factor composed of several domains important for hormone binding, DNA binding, and activation of transcription. The protein localizes to the nucleus where it may form a homodimer or a heterodimer with estrogen receptor 2. Estrogen and its receptors are essential for sexual development and reproductive function, but also play a role in other tissues such as bone. Estrogen receptors are also involved in pathological processes including breast cancer, endometrial cancer, and osteoporosis. Alternative splicing results in several transcript variants, which differ in their 5' UTRs and use different promoters. [provided by NCBI RefSeq, Jul 2008]
**ESR2**	**Erb; ESRB; ESTRB; NR3A2; ER-BETA;**	estrogen receptor 2 (ER beta)	NM_001040275.1; NM_001214902.1; NM_001214903.1; NM_001271876.1; NM_001271877.1; NM_001437.2;	This gene encodes a member of the family of estrogen receptors and superfamily of nuclear receptor transcription factors. The gene product contains an N-terminal DNA binding domain and C-terminal ligand binding domain and is localized to the nucleus, cytoplasm, and mitochondria. Upon binding to 17beta-estradiol or related ligands, the encoded protein forms homo- or hetero-dimers that interact with specific DNA sequences to activate transcription. Some isoforms dominantly inhibit the activity of other estrogen receptor family members. Several alternatively spliced transcript variants of this gene have been described, but the full-length nature of some of these variants has not been fully characterized. [provided by NCBI RefSeq, Jul 2008]
**FCER2**	**CD23**	Fc fragment of IgE, low affinity II, receptor for (CD23)	NM_002002	The protein encoded by this gene is a B-cell specific antigen, and a low-affinity receptor for IgE. It has essential roles in B cell growth and differentiation, and the regulation of IgE production. This protein also exists as a soluble secreted form, then functioning as a potent mitogenic growth factor. Alternatively spliced transcript variants encoding different isoforms have been described for this gene.[provided by NCBI RefSeq, Jul 2011]
**ID2**	**GIG8; ID2A; ID2H; bHLHb26**	inhibitor of DNA binding 2, dominant negative helix-loop-helix protein	NM_002166.4	The protein encoded by this gene belongs to the inhibitor of DNA binding family, members of which are transcriptional regulators that contain a helix-loop-helix (HLH) domain but not a basic domain. Members of the inhibitor of DNA binding family inhibit the functions of basic helix-loop-helix transcription factors in a dominant-negative manner by suppressing their heterodimerization partners through the HLH domains. This protein may play a role in negatively regulating cell differentiation. A pseudogene of this gene is located on chromosome 3. [provided by NCBI RefSeq, Aug 2011]

Table S3 (continued) Genes composing the AID/APOBEC multigene signature. Gene symbols, synonyms, full names, accession numbers and a short description from NCBI are provided.

Symbol	Synonym	Name	NCBI accession number	Short functional description
**ID3**	**HEIR-1; bHLHb25**	inhibitor of DNA binding 3, dominant negative helix-loop-helix protein	NM_002167.4	The protein encoded by this gene is a helix-loop-helix (HLH) protein that can form heterodimers with other HLH proteins. However, the encoded protein lacks a basic DNA-binding domain and therefore inhibits the DNA binding of any HLH protein with which it interacts. [provided by NCBI RefSeq, Aug 2011]
**NANOG**		Nanog homeobox	NM_024865	Transcription regulator involved in inner cell mass and embryonic stem (ES) cells proliferation and self-renewal. Imposes pluripotency on ES cells and prevents their differentiation towards extraembryonic endoderm and trophectoderm lineages. Blocks bone morphogenetic protein-induced mesoderm differentiation of ES cells by physically interacting with SMAD1 and interfering with the recruitment of coactivators to the active SMAD transcriptional complexes. Acts as a transcriptional activator or repressor. Binds optimally to the DNA consensus sequence 5'-TAAT[GT][GT]-3' or 5'-[CG][GA][CG]C[GC]ATTAN[GC]-3'. Able to autorepress its expression in differentiating (ES) cells: binds to its own promoter following interaction with ZNF281/ZFP281, leading to recruitment of the NuRD complex and subsequent repression of expression. When overexpressed, promotes cells to enter into S phase and proliferation. [provided by UniProtKB/Swiss-Prot]
**NUGGC**	**SLIP-GC; C8orf80**	nuclear GTPase, germinal center associated	NM_001010906	Plays a role as replication-related GTPase protein in germinal center B-cell. [provided by UniProtKB/Swiss-Prot]
**PAX5**	**BSAP**	paired box 5	NM_001280547.1; NM_001280548.1; NM_001280549.1; NM_001280550.1; NM_001280551.1; NM_001280552.1; NM_001280553.1; NM_001280554.1; NM_001280555.1; NM_001280556.1; NM_016734.2	This gene encodes a member of the paired box (PAX) family of transcription factors. The central feature of this gene family is a novel, highly conserved DNA-binding motif, known as the paired box. Paired box transcription factors are important regulators in early development, and alterations in the expression of their genes are thought to contribute to neoplastic transformation. This gene encodes the B-cell lineage specific activator protein that is expressed at early, but not late stages of B-cell differentiation. Its expression has also been detected in developing CNS and testis and so the encoded protein may also play a role in neural development and spermatogenesis. This gene is located at 9p13, which is involved in t(9;14)(p13;q32) translocations recurring in small lymphocytic lymphomas of the plasmacytoid subtype, and in derived large-cell lymphomas. This translocation brings the potent E-mu enhancer of the IgH gene into close proximity of the PAX5 promoter, suggesting that the deregulation of transcription of this gene contributes to the pathogenesis of these lymphomas. Alternative splicing results in multiple transcript variants encoding different isoforms. [provided by NCBI RefSeq, Jul 2013]
**PRDM1**	**BLIMP1**	PR domain containing 1, with ZNF domain	NM_001198	This gene encodes a protein that acts as a repressor of beta-interferon gene expression. The protein binds specifically to the PRDI (positive regulatory domain I element) of the beta-IFN gene promoter. Transcription of this gene increases upon virus induction. Two alternatively spliced transcript variants that encode different isoforms have been reported. [provided by NCBI RefSeq, Jul 2008]
**PTPRC**	**CD45; LY5; B220; CD45R**	protein tyrosine phosphatase, receptor type, C	NM_001267798.1; NM_002838.4; NM_080921.3	The protein encoded by this gene is a member of the protein tyrosine phosphatase (PTP) family. PTPs are known to be signaling molecules that regulate a variety of cellular processes including cell growth, differentiation, mitosis, and oncogenic transformation. This PTP contains an extracellular domain, a single transmembrane segment and two tandem intracytoplasmic catalytic domains, and thus is classified as a receptor type PTP. This PTP has been shown to be an essential regulator of T- and B-cell antigen receptor signaling. It functions through either direct interaction with components of the antigen receptor complexes, or by activating various Src family kinases required for the antigen receptor signaling. This PTP also suppresses JAK kinases, and thus functions as a regulator of cytokine receptor signaling. Alternatively spliced transcripts variants of this gene, which encode distinct isoforms, have been reported. [provided by NCBI RefSeq, Jun 2012]
**XRCC5**	**KU80**	X-ray repair complementing defective repair in Chinese hamster cells 5 (double-strand-break rejoining)	NM_021141	The protein encoded by this gene is the 80-kilodalton subunit of the Ku heterodimer protein which is also known as ATP-dependant DNA helicase II or DNA repair protein XRCC5. Ku is the DNA-binding component of the DNA-dependent protein kinase, and it functions together with the DNA ligase IV-XRCC4 complex in the repair of DNA double-strand break by non-homologous end joining and the completion of V(D)J recombination events. This gene functionally complements Chinese hamster xrs-6, a mutant defective in DNA double-strand break repair and in ability to undergo V(D)J recombination. A rare microsatellite polymorphism in this gene is associated with cancer in patients of varying radiosensitivity. [provided by NCBI RefSeq, Jul 2008]
**XRCC6**	**KU70**	X-ray repair complementing defective repair in Chinese hamster cells 6	NM_001469.3	The p70/p80 autoantigen is a nuclear complex consisting of two subunits with molecular masses of approximately 70 and 80 kDa. The complex functions as a single-stranded DNA-dependent ATP-dependent helicase. The complex may be involved in the repair of nonhomologous DNA ends such as that required for double-strand break repair, transposition, and V(D)J recombination. High levels of autoantibodies to p70 and p80 have been found in some patients with systemic lupus erythematosus. [provided by NCBI RefSeq, Jul 2008]

Table S4 Univariate Cox regression analysis of clinicopathological variables and gene profiling-derived data sets for OS and PFS.

**Characteristics**	**OS**		**PFS**	
	HR (95% CI)	*P*	HR (95% CI)	*P*
Age	**1.03 (1.01 - 1.06)**	**0.011**	1.01 (0.99 - 1.03)	0.316
Histology	1.44 (0.70 - 2.94)	0.323	0.97 (0.54 - 1.73)	0.908
FIGO stage	**1.83 (1.02 - 3.29)**	**0.044**	**2.33 (1.53 - 3.54)**	**0.000**
Grading	**2.10 (1.03 - 4.31)**	**0.042**	**1.60 (1.00 - 2.54)**	**0.048**
Peritoneal carcinomatosis	**4.17 (1.78 - 9.78)**	**0.001**	**3.88 (2.27 - 6.61)**	**0.000**
Residual disease	**1.98 (1.15 - 3.44)**	**0.014**	**1.95 (1.29 - 2.94)**	**0.002**
AICDA	**1.18 (1.04 - 1.33)**	**0.008**	1.00 (0.92 - 1.08)	0.904
APOBEC3A	0.98 (0.86 - 1.12)	0.779	0.96 (0.88 - 1.06)	0.450
APOBEC3B	1.07 (0.92 - 1.24)	0.387	1.05 (0.94 - 1.17)	0.376
APOBEC3C	1.02 (0.82 - 1.27)	0.839	0.99 (0.85 - 1.16)	0.893
APOBEC3D	1.03 (0.87 - 1.22)	0.708	1.00 (0.90 - 1.11)	0.968
APOBEC3F	1.07 (0.84 - 1.36)	0.574	1.06 (0.89 - 1.27)	0.490
APOBEC3G	0.92 (0.80 - 1.08)	0.308	0.91 (0.82 - 1.02)	0.091
APOBEC3H	1.02 (0.88 - 1.20)	0.758	0.94 (0.86 - 1.03)	0.173
APOBEC4	1.01 (0.86 - 1.18)	0.898	1.03 (0.92 - 1.16)	0.628
ESR1	0.91 (0.78 - 1.05)	0.182	0.92 (0.83 - 1.02)	0.121
ESR2	0.99 (0.93 - 1.07)	0.867	0.96 (0.91 - 1.01)	0.154
FCER2 (CD23)	1.00 (0.90 - 1.12)	0.949	1.00 (0.93 - 1.07)	0.989
ID2	1.09 (0.89 - 1.34)	0.393	1.02 (0.91 - 1.14)	0.754
ID3	**1.36 (1.12 - 1.64)**	**0.002**	1.03 (0.93 - 1.15)	0.557
NANOG	1.03 (0.87 - 1.22)	0.756	0.98 (0.88 - 1.10)	0.767
NUGGC (SLIP-GC)	1.05 (0.91 - 1.21)	0.496	1.01 (0.92 - 1.12)	0.781
PAX5	1.01 (0.90 - 1.12)	0.899	1.00 (0.93 - 1.08)	0.947
PRDM1 (BLIMP1)	1.22 (0.99 - 1.50)	0.061	0.98 (0.89 - 1.08)	0.733
PTPRC (CD45)	1.15 (0.98 - 1.34)	0.079	1.00 (0.91 - 1.01)	0.979
XRCC5 (KU80)	1.02 (0.82 - 1.26)	0.873	0.96 (0.84 - 1.09)	0.532
XRCC6 (KU70)	1.16 (0.87 - 1.54)	0.325	1.00 (0.87 - 1.14)	0.955

Histology (non-serous vs. serous) was encoded as “0” for serous and “1” for non-serous; Peritoneal carcinomatosis (yes vs. no) was encoded as “0” for no and “1” for yes; Grading (Grade 1 and 2 vs. 3) was encoded as “0” for Grade 1 and 2 and “1” for Grade 3; Residual disease (yes vs. no) was encoded as “0” for no and “1” for yes. HR, hazard ratio; CI, confidence interval; bold, statistically significant.

Table S5 Correlation analysis for the AID/APOBEC multigene-derived variables performed across 186 ovarian carcinoma patients.

		AICDA	APOBEC3A	APOBEC3B	APOBEC3C	APOBEC3D	APOBEC3F	APOBEC 3G	APOBEC3H	APOBEC4	PAX5	ID2	ID3	PTPRC (CD45)	XRCC6 (Ku70)	XRCC5 (Ku80)	NUGGC	ESR1	ESR2	PRDM1	FCER2	NANOG
AICDA	Pearson corr.	1.000																				
	Sig. (2-tailed)																					
	Bonf.-H. corr.																					
APOBEC3A	Pearson corr.	0.196	1.000																			
	Sig. (2-tailed)	0.007																				
	Bonf.-H. corr.	0.524																				
APOBEC3B	Pearson corr.	0.158	0.229	1.000																		
	Sig. (2-tailed)	0.032	0.002																			
	Bonf.-H. corr.	1.000	0.125																			
APOBEC3C	Pearson corr.	**0.327**	0.243	0.230	1.000																	
	Sig. (2-tailed)	<0.001	0.001	0.002																		
	Bonf.-H. corr.	**0.001**	0.066	0.119																		
APOBEC3D	Pearson corr.	**0.288**	**0.540**	0.053	**0.554**	1.000																
	Sig. (2-tailed)	<0.001	<0.001	0.474	<0.001																	
	Bonf.-H. corr.	**0.006**	**<0.001**	1.000	**<0.001**																	
APOBEC3F	Pearson corr.	0.185	**0.260**	**0.321**	**0.776**	**0.558**	1.000															
	Sig. (2-tailed)	0.012	<0.001	<0.001	<0.001	<0.001																
	Bonf.-H. corr.	0.782	**0.030**	**0.001**	**<0.001**	**<0.001**																
APOBEC 3G	Pearson corr.	**0.263**	**0.397**	0.125	**0.550**	**0.709**	**0.456**	1.000														
	Sig. (2-tailed)	<0.001	<0.001	0.088	<0.001	<0.001	<0.001															
	Bonf.-H. corr.	**0.025**	**<0.001**	1.000	**<0.001**	**<0.001**	**<0.001**															
APOBEC3H	Pearson corr.	**0.266**	**0.386**	0.097	**0.388**	**0.669**	**0.314**	**0.529**	1.000													
	Sig. (2-tailed)	<0.001	<0.001	0.187	<0.001	<0.001	<0.001	<0.001														
	Bonf.-H. corr.	**0.022**	**<0.001**	1.000	**<0.001**	**<0.001**	**0.001**	**<0.001**														
APOBEC4	Pearson corr.	-0.056	0.063	0.169	**0.293**	0.181	**0.475**	0.098	0.160	1.000												
	Sig. (2-tailed)	0.448	0.390	0.021	<0.001	0.014	<0.001	0.185	0.029													
	Bonf.-H. corr.	1.000	1.000	1.000	**0.005**	0.902	**<0.001**	1.000	1.000													
PAX5	Pearson corr.	**0.311**	**0.340**	0.153	**0.375**	**0.503**	**0.318**	**0.418**	**0.330**	0.02	1.000											
	Sig. (2-tailed)	<0.001	<0.001	0.036	<0.001	<0.001	<0.001	<0.001	<0.001	0.788												
	Bonf.-H. corr.	**0.002**	**<0.001**	1.000	**<0.001**	**<0.001**	**0.001**	**<0.001**	**<0.001**	1.000												
ID2	Pearson corr.	0.241	**0.441**	0.000	**0.422**	**0.554**	**0.297**	**0.442**	**0.384**	0.074	**0.303**	1.000										
	Sig. (2-tailed)	0.001	<0.001	0.999	<0.001	<0.001	<0.001	<0.001	<0.001	0.313	<0.001											
	Bonf.-H. corr.	0.072	**<0.001**	1.000	**<0.001**	**<0.001**	**0.004**	**<0.001**	**<0.001**	1.000	**0.003**											
ID3	Pearson corr.	0.248	**0.263**	-0.067	**0.357**	**0.544**	**0.337**	**0.444**	**0.335**	0.156	**0.281**	**0.653**	1.000									
	Sig. (2-tailed)	0.001	<0.001	0.363	<0.001	<0.001	<0.001	<0.001	<0.001	0.033	<0.001	<0.001										
	Bonf.-H. corr.	0.053	**0.026**	1.000	**<0.001**	**<0.001**	**<0.001**	**<0.001**	**<0.001**	1.000	**0.010**	**<0.001**										
PTPRC (CD45)	Pearson corr.	**0.521**	**0.442**	0.234	**0.604**	**0.686**	**0.398**	**0.619**	**0.607**	-0.074	**0.599**	**0.567**	**0.483**	1.000								
	Sig. (2-tailed)	<0.001	<0.001	0.001	<0.001	<0.001	<0.001	<0.001	<0.001	0.316	<0.001	<0.001	<0.001									
	Bonf.-H. corr.	**<0.001**	**<0.001**	0.099	**<0.001**	**<0.001**	**<0.001**	**<0.001**	**<0.001**	1.000	**<0.001**	**<0.001**	**<0.001**									
XRCC6 (Ku70)	Pearson corr.	**0.276**	**0.464**	0.146	**0.450**	**0.726**	**0.524**	**0.526**	**0.529**	**0.296**	**0.304**	**0.632**	**0.643**	**0.540**	1.000							
	Sig. (2-tailed)	<0.001	<0.001	0.046	<0.001	<0.001	<0.001	<0.001	<0.001	<0.001	<0.001	<0.001	<0.001	<0.001								
	Bonf.-H. corr.	**0.012**	**<0.001**	1.000	**<0.001**	**<0.001**	**<0.001**	**<0.001**	**<0.001**	**0.004**	**0.002**	**<0.001**	**<0.001**	**<0.001**								
XRCC5 (Ku80)	Pearson corr.	0.212	**0.432**	0.131	**0.286**	**0.581**	**0.495**	**0.443**	**0.457**	**0.449**	**0.26**	**0.478**	**0.525**	**0.331**	**0.811**	1.000						
	Sig. (2-tailed)	0.004	<0.001	0.074	<0.001	<0.001	<0.001	<0.001	<0.001	<0.001	<0.001	<0.001	<0.001	<0.001	<0.001							
	Bonf.-H. corr.	0.265	**<0.001**	1.000	**0.007**	**<0.001**	**<0.001**	**<0.001**	**<0.001**	**<0.001**	**0.029**	**<0.001**	**<0.001**	**<0.001**	**<0.001**							
NUGGC (SLIP-GC)	Pearson corr.	**0.321**	**0.394**	0.088	**0.475**	**0.628**	**0.373**	**0.547**	**0.528**	0.132	**0.546**	**0.480**	**0.479**	**0.638**	**0.476**	**0.410**	1.000					
	Sig. (2-tailed)	<0.001	<0.001	0.230	<0.001	<0.001	<0.001	<0.001	<0.001	0.072	<0.001	<0.001	<0.001	<0.001	<0.001	<0.001						
	Bonf.-H. corr.	**0.001**	**<0.001**	1.000	**<0.001**	**<0.001**	**<0.001**	**<0.001**	**<0.001**	1.000	**<0.001**	**<0.001**	**<0.001**	**<0.001**	**<0.001**	**<0.001**						
ESR1	Pearson corr.	-0.051	-0.021	0.031	0.140	0.225	0.193	0.150	0.169	0.135	-0.001	-0.080	-0.048	0.040	0.170	0.164	0.123	1.000				
	Sig. (2-tailed)	0.493	0.780	0.679	0.058	0.002	0.008	0.041	0.021	0.065	0.986	0.278	0.514	0.586	0.020	0.025	0.095					
	Bonf.-H. corr.	1.000	1.000	1.000	1.000	0.145	0.573	1.000	1.000	1.000	1.000	1.000	1.000	1.000	1.000	1.000	1.000					
ESR2	Pearson corr.	-0.082	-0.088	-0.173	0.077	0.045	0.038	0.110	-0.087	0.167	-0.023	0.049	0.116	-0.066	0.023	-0.060	0.158	0.131	1.000			
	Sig. (2-tailed)	0.265	0.231	0.018	0.298	0.545	0.605	0.136	0.236	0.023	0.754	0.505	0.116	0.374	0.751	0.416	0.031	0.074				
	Bonf.-H. corr.	1.000	1.000	1.000	1.000	1.000	1.000	1.000	1.000	1.000	1.000	1.000	1.000	1.000	1.000	1.000	1.000	1.000				
PRDM1 (BLIMP1)	Pearson corr.	**0.408**	**0.451**	0.095	**0.398**	**0.705**	**0.399**	**0.538**	**0.584**	0.159	**0.396**	**0.590**	**0.732**	**0.679**	**0.734**	**0.657**	**0.632**	0.046	0.000	1.000		
	Sig. (2-tailed)	<0.001	<0.001	0.197	<0.001	<0.001	<0.001	<0.001	<0.001	0.030	<0.001	<0.001	<0.001	<0.001	<0.001	<0.001	<0.001	0.534	0.995			
	Bonf.-H. corr.	**<0.001**	**<0.001**	1.000	**<0.001**	**<0.001**	**<0.001**	**<0.001**	**<0.001**	1.000	**<0.001**	**<0.001**	**<0.001**	**<0.001**	**<0.001**	**<0.001**	**<0.001**	1.000	1.000			
FCER2 (CD23)	Pearson corr.	0.249	0.245	0.112	**0.416**	**0.375**	**0.332**	**0.384**	0.178	0.071	**0.621**	0.245	**0.287**	**0.473**	0.240	0.190	**0.447**	-0.039	0.063	**0.374**	1.000	
	Sig. (2-tailed)	0.001	0.001	0.129	<0.001	<0.001	<0.001	<0.001	0.015	0.338	<0.001	0.001	<0.001	<0.001	0.001	0.009	<0.001	0.593	0.390	<0.001		
	Bonf.-H. corr.	0.051	0.060	1.000	**<0.001**	**<0.001**	**<0.001**	**<0.001**	0.990	1.000	**<0.001**	0.060	**0.007**	**<0.001**	0.075	0.644	**<0.001**	1.000	1.000	**<0.001**		
NANOG	Pearson corr.	0.090	**0.302**	0.051	0.237	**0.548**	**0.477**	**0.379**	**0.430**	**0.509**	0.147	**0.391**	**0.464**	0.220	**0.688**	**0.789**	**0.306**	**0.257**	0.061	**0.569**	0.101	1.000
	Sig. (2-tailed)	0.220	<0.001	0.492	0.001	<0.001	<0.001	<0.001	<0.001	<0.001	0.045	<0.001	<0.001	0.003	<0.001	<0.001	<0.001	<0.001	0.406	<0.001	0.171	
	Bonf.-H. corr.	1.000	**0.003**	1.000	0.087	**<0.001**	**<0.001**	**<0.001**	**<0.001**	**<0.001**	1.000	**<0.001**	**<0.001**	0.185	**<0.001**	**<0.001**	**0.002**	**0.033**	1.000	**<0.001**	1.000	

Pearson correlation matrix *(Pearson corr.*) and the corresponding matrix of probabilities [*Sig. (2-tailed)*] as well as matrix of Bonferroni-Holm probabilities (*Bonf.-H. corr.*) are shown. Color code for statistically significant co-regularities: *red* for correlation coefficient ≥0.6; *blue* for correlation coefficient <0.6; statistically significant values are highlighted in bold.

Table S6 Relative importance of individual variables in multivariable models (ridge) for PFS.

Clinics

Pos.	Variables	beta	HR	STDBETA
1.	Peritoneal carcinomatosis	0.77	2.15	0.35
2.	Residual disease	0.33	1.39	0.15
3.	FIGO stage	0.41	1.51	0.15
4.	Histology	0.27	1.31	0.09
5.	Grading	0.19	1.21	0.09
6.	Age	0.00	1.00	0.05

AID/APOBEC

Pos.	Variables	beta	HR	STDBETA
	AICDA	[A]		
	APOBEC3A	[A]		
	APOBEC3B	[A]		
	APOBEC3C	[A]		
	APOBEC3D	[A]		
	APOBEC3F	[A]		
	APOBEC3G	[A]		
	APOBEC3H	[A]		
	APOBEC4	[A]		
	ESR1	[A]		
	ESR2	[A]		
	FCER2	[A]		
	ID2	[A]		
	ID3	[A]		
	NANOG	[A]		
	NUGGC	[A]		
	PAX5	[A]		
	PRDM1	[A]		
	PTPRC (CD45)	[A]		
	XRCC5 (Ku80)	[A]		
	XRCC6 (Ku70)	[A]		

Combined

Pos.	Variables	beta	HR	STDBETA
1.	Peritoneal carcinomatosis	0.56	1.75	0.25
2.	Residual disease	0.27	1.32	0.12
3.	FIGO stage	0.30	1.35	0.11
4.	*APOBEC3G*	-0.05	0.95	-0.09
5.	Grading	0.16	1.18	0.07
6.	*APOBEC3D*	0.04	1.04	0.06
7.	*APOBEC3H*	-0.03	0.97	-0.06
8.	*ID3*	0.03	1.03	0.05
9.	*ESR2*	-0.01	0.99	-0.05
10.	Histology	0.15	1.16	0.05
11.	*ESR1*	-0.02	0.98	-0.04
12.	*APOBEC3A*	-0.02	0.98	-0.04
13.	*APOBEC3F*	0.04	1.04	0.04
14.	Age	0.00	1.00	0.04
15.	*APOBEC3B*	0.02	1.02	0.03
16.	*APOBEC4*	0.02	1.02	0.03
17.	*PTPRC (CD45)*	0.02	1.02	0.03
18.	*NUGGC*	0.01	1.02	0.03
19.	*ID2*	0.01	1.02	0.03
20.	*XRCC5 (Ku80)*	-0.02	0.98	-0.03
21.	*XRCC6 (Ku70)*	0.02	1.02	0.02
22.	*PRDM1*	-0.01	0.99	-0.02
23.	*APOBEC3C*	-0.01	0.99	-0.02
24.	*FCER2*	0.00	1.00	0.01
25.	*AICDA*	0.00	1.00	-0.01
26.	*PAX5*	0.00	1.00	0.00
27.	*NANOG*	0.00	1.00	0.00

Histology (non-serous vs. serous) was encoded as "0" for serous and "1" for non-serous; Peritoneal carcinomatosis (yes vs. no) was encoded as "0" for no and "1" for yes; Grading (Grade 1 and 2 vs. 3) was encoded as "0" for Grade 1 and 2 and "1" for Grade 3; Residual disease (yes vs. no) was encoded as "0" for no and "1" for yes. beta, regression coefficient (log hazard ratio), HR, hazard ratio, STDBETA, standardized regression coefficients. [A] not considered for stable model-building.

Table S7 Comparative analysis of multivariable models (LASSO) for prognostication of OS and PFS.

	OS			PFS		
	**PEV %**	**c-index**	***P***	**PEV %**	**c-index**	***P***
Clinics	7.54	0.67	<0.001	16.26	0.64	<0.001
AID/APOBEC	5.76	0.64	<0.001	n.a.	n.a.	n.a.
Combined	10.80	0.70	<0.001; 0.006*	15.49	0.61	<0.001, 0.380*

Results of Cox regression model (LASSO) including a leave-one-out cross-validation resampling procedure are shown. P-values were calculated by univariate Cox regression using the cross-validated predictors from each model. Models were built up for (i) clinicopathological variables designated as Clinics; (ii) AID/APOBEC multigene-based variables designated as AID/APOBEC; (iii) their combination designated as Combined. PEV, proportion of explained variation; c-index, concordance index.* p-value for added value of AID/APOBEC on top of Clinics in bivariable models with cross-validated predictors.

Table S8 Relative importance of individual variables in multivariable models (LASSO) for OS.

Clinics

Pos.	Variables	beta	HR	STDBETA
1.	Peritoneal carcinomatosis	1.13	3.11	0.51
2.	Age	0.03	1.03	0.32
3.	Histology	0.68	1.98	0.22
4.	FIGO stage	0.49	1.63	0.18
5.	Residual disease	0.31	1.37	0.14
6.	Grading	0.29	1.34	0.13

AID/APOBEC

Pos.	Variables	beta	HR	STDBETA
1.	*ID3*	0.26	1.30	0.45
2.	*AICDA*	0.11	1.12	0.26
3.	*APOBEC3G*	-0.16	0.85	-0.26
4.	*APOBEC3B*	0.02	1.02	0.04
5.	*ESR1*	-0.02	0.98	-0.04
	APOBEC3A	A		
	APOBEC3C	A		
	APOBEC3D	A		
	APOBEC3F	A		
	APOBEC3H	A		
	APOBEC4	A		
	ESR2	A		
	FCER2	A		
	ID2	A		
	NANOG	A		
	NUGGC	A		
	PAX5	A		
	PRDM1	A		
	PTPRC (CD45)	A		
	XRCC5 (Ku80)	A		
	XRCC6 (Ku70)	A		

Combined

Pos.	Variables	beta	HR	STDBETA
1.	*ID3*	0.24	1.27	0.41
2.	Peritoneal carcinomatosis	0.89	2.43	0.40
3.	*AICDA*	0.11	1.12	0.27
4.	Age	0.02	1.02	0.26
5.	*APOBEC3G*	-0.13	0.88	-0.21
6.	Grading	0.31	1.36	0.14
7.	Histology	0.37	1.45	0.12
8.	Residual disease	0.22	1.25	0.10
9.	FIGO stage	0.24	1.28	0.09
	APOBEC3A	A		
	APOBEC3B	A		
	APOBEC3C	A		
	APOBEC3D	A		
	APOBEC3F	A		
	APOBEC3H	A		
	APOBEC4	A		
	ESR1	A		
	ESR2	A		
	FCER2	A		
	ID2	A		
	NANOG	A		
	NUGGC	A		
	PAX5	A		
	PRDM1	A		
	PTPRC (CD45)	A		
	XRCC5 (Ku80)	A		
	XRCC6 (Ku70)	A		

Histology (non-serous vs. serous) was encoded as “0” for serous and “1” for non-serous; Peritoneal carcinomatosis (yes vs. no) was encoded as “0” for no and “1” for yes; Grading (Grade 1 and 2 vs. 3) was encoded as “0” for Grade 1 and 2 and “1” for Grade 3; Residual disease (yes vs. no) was encoded as “0” for no and “1” for yes. beta, regression coefficient (log hazard ratio), HR, hazard ratio, STDBETA, standardized regression coefficients; A not selected by cumulative inclusion of covariates into model.

Table S9 Relative importance of individual variables in multivariable models (LASSO) for PFS.

Clinics

Pos.	Variables	beta	HR	STDBETA
1.	Peritoneal carcinomatosis	1.06	2.89	0.48
2.	FIGO stage	0.43	1.53	0.16
3.	Residual disease	0.28	1.33	0.13
4.	Histology	0.23	1.26	0.08
5.	Grading	0.11	1.11	0.05
	Age	A		

AID/APOBEC

Pos.	Variables	beta	HR	STDBETA
	AICDA	B		
	APOBEC3A	B		
	APOBEC3B	B		
	APOBEC3C	B		
	APOBEC3D	B		
	APOBEC3F	B		
	APOBEC3G	B		
	APOBEC3H	B		
	APOBEC4	B		
	FCER2	B		
	ESR1	B		
	ESR2	B		
	ID2	B		
	ID3	B		
	NANOG	B		
	NUGGC	B		
	PAX5	B		
	PRDM1	B		
	PTPRC (CD45)	B		
	XRCC5 (Ku80)	B		
	XRCC6 (Ku70)	B		

Combined

Pos.	Variables	beta	HR	STDBETA
1.	Peritoneal carcinomatosis	0.91	2.49	0.41
2.	FIGO stage	0.29	1.34	0.11
3.	Residual disease	0.20	1.22	0.09
4.	Grading	0.04	1.04	0.02
	Age	A		
	Histology	A		
	AICDA	A		
	APOBEC3A	A		
	APOBEC3B	A		
	APOBEC3C	A		
	APOBEC3D	A		
	APOBEC3F	A		
	APOBEC3G	A		
	APOBEC3H	A		
	APOBEC4	A		
	FCER2	A		
	ESR1	A		
	ESR2	A		
	ID2	A		
	ID3	A		
	NANOG	A		
	NUGGC	A		
	PAX5	A		
	PRDM1	A		
	PTPRC (CD45)	A		
	XRCC5 (Ku80)	A		
	XRCC6 (Ku70)	A		

Histology (non-serous vs. serous) was encoded as “0” for serous and “1” for non-serous; Peritoneal carcinomatosis (yes vs. no) was encoded as “0” for no and “1” for yes; Grading (Grade 1 and 2 vs. 3) was encoded as “0” for Grade 1 and 2 and “1” for Grade 3; Residual disease (yes vs. no) was encoded as “0” for no and “1” for yes. beta, regression coefficient (log hazard ratio), HR, hazard ratio, STDBETA, standardized regression coefficients; A not selected by cumulative inclusion of covariates into model; B not considered for stable model-building.

Table S10 The top 50 Affymetrix probe sets co-regulated with *APOBEC3G* in ovarian cancer.

Rank	Score	Probe set	Gene Symbol	Description
	1.000	204205_at	APOBEC3G	apolipoprotein B mRNA editing enzyme, catalytic polypeptide-like 3G
1	0.811	221087_s_at	APOL3	apolipoprotein L, 3
2	0.799	239587_at	TLR3	toll-like receptor 3
3	0.798	211368_s_at	CASP1	caspase 1, apoptosis-related cysteine peptidase
4	0.787	227210_at	SFMBT2	Scm-like with four mbt domains 2
5	0.784	206271_at	TLR3	toll-like receptor 3
6	0.783	1552701_a_at	CARD16	caspase recruitment domain family, member 16
7	0.780	1552703_s_at	CASP1,	caspase 1, apoptosis-related cysteine peptidase,caspase recruitment
8	0.779	204070_at	RARRES3	retinoic acid receptor responder (tazarotene induced) 3
9	0.775	204646_at	DPYD	dihydropyrimidine dehydrogenase
10	0.773	209584_x_at	APOBEC3C	apolipoprotein B mRNA editing enzyme, catalytic polypeptide-like 3C
11	0.772	203148_s_at	TRIM14	tripartite motif containing 14
12	0.768	213537_at	HLA-DPA1	major histocompatibility complex, class II, DP alpha 1
13	0.768	223502_s_at	TNFSF13B	tumor necrosis factor (ligand) superfamily, member 13b
14	0.767	211990_at	HLA-DPA1	major histocompatibility complex, class II, DP alpha 1
15	0.766	201137_s_at	HLA-DPB1	major histocompatibility complex, class II, DP beta 1
16	0.766	209970_x_at	CASP1	caspase 1, apoptosis-related cysteine peptidase
17	0.763	238725_at	IRF1	interferon regulatory factor 1
18	0.762	205992_s_at	IL15	interleukin 15
19	0.754	232617_at	CTSS	cathepsin S
20	0.754	219666_at	MS4A6A	membrane-spanning 4-domains, subfamily A, member 6A
21	0.754	228617_at	XAF1	XIAP associated factor 1
22	0.753	214995_s_at	APOBEC3G, APOBEC3F	apolipoprotein B mRNA editing enzyme, catalytic polypeptide-like 3G,apolipoprotein B mRNA editing enzyme, catalytic polypeptide-like 3F
23	0.750	204279_at	PSMB9	proteasome (prosome, macropain) subunit, beta type, 9 (large
24	0.748	1553906_s_at	FGD2	FYVE, RhoGEF and PH domain containing 2
25	0.746	228532_at	C1orf162	chromosome 1 open reading frame 162
26	0.746	204655_at	CCL5	chemokine (C-C motif) ligand 5
27	0.746	213293_s_at	TRIM22	tripartite motif containing 22
28	0.745	230036_at	SAMD9L	sterile alpha motif domain containing 9-like
29	0.743	204446_s_at	ALOX5	arachidonate 5-lipoxygenase
30	0.743	228152_s_at	DDX60L	DEAD (Asp-Glu-Ala-Asp) box polypeptide 60-like
31	0.743	229041_s_at	LOC100505746	uncharacterized LOC100505746
32	0.740	229723_at	TAGAP	T-cell activation RhoGTPase activating protein
33	0.738	219716_at	APOL6	apolipoprotein L, 6
34	0.737	1555852_at	LOC100507463	uncharacterized LOC100507463
35	0.737	1557116_at	APOL6	apolipoprotein L, 6
36	0.731	209354_at	TNFRSF14	tumor necrosis factor receptor superfamily, member 14
37	0.730	224414_s_at	CARD6	caspase recruitment domain family, member 6
38	0.730	214255_at	ATP10A	ATPase, class V, type 10A
39	0.730	219209_at	IFIH1	interferon induced with helicase C domain 1
40	0.730	229968_at		
41	0.729	205786_s_at	ITGAM	integrin, alpha M (complement component 3 receptor 3 subunit)
42	0.728	1405_i_at	CCL5	chemokine (C-C motif) ligand 5
43	0.727	202748_at	GBP2	guanylate binding protein 2, interferon-inducible
44	0.725	202659_at	PSMB10	proteasome (prosome, macropain) subunit, beta type, 10
45	0.723	227188_at	C21orf63	chromosome 21 open reading frame 63
46	0.722	221698_s_at	CLEC7A	C-type lectin domain family 7, member A
47	0.722	241869_at	APOL6	apolipoprotein L, 6
48	0.722	205101_at	CIITA	class II, major histocompatibility complex, transactivator
49	0.720	211366_x_at	CASP1	caspase 1, apoptosis-related cysteine peptidase
50	0.719	226878_at	HLA-DOA	major histocompatibility complex, class II, DO alpha

The probe sets were identified using GENEVESTIGATOR and ranked according to the Pearson correlation coefficient (Score).

Table S11 The top 50 Affymetrix probe sets co-regulated with *ESR1* in ovarian cancer.

Rank	Score	Probe set	Gene Symbol	Description
	1.000	205225_at	ESR1	estrogen receptor 1
1	0.809	209692_at	EYA2	eyes absent homolog 2 (Drosophila)
2	0.802	215552_s_at	ESR1	estrogen receptor 1
3	0.779	211235_s_at	ESR1	estrogen receptor 1
4	0.767	1559477_s_at	MEIS1	Meis homeobox 1
5	0.762	209794_at	SRGAP3	SLIT-ROBO Rho GTPase activating protein 3
6	0.759	204069_at	MEIS1	Meis homeobox 1
7	0.750	230943_at	SOX17	SRY (sex determining region Y)-box 17
8	0.744	211234_x_at	ESR1	estrogen receptor 1
9	0.742	222835_at	THSD4	thrombospondin, type I, domain containing 4
10	0.740	211233_x_at	ESR1	estrogen receptor 1
11	0.736	242172_at	MEIS1	Meis homeobox 1
12	0.736	219440_at	RAI2	retinoic acid induced 2
13	0.722	225996_at	LONRF2	LON peptidase N-terminal domain and ring finger 2
14	0.721	219993_at	SOX17	SRY (sex determining region Y)-box 17
15	0.719	232869_at	SRGAP3	SLIT-ROBO Rho GTPase activating protein 3
16	0.718	225623_at	KIAA1737	KIAA1737
17	0.716	204187_at	GMPR	guanosine monophosphate reductase
18	0.706	231192_at	LPAR3	lysophosphatidic acid receptor 3
19	0.704	220196_at	MUC16	mucin 16, cell surface associated
20	0.703	1554480_a_at	ARMC10	armadillo repeat containing 10
21	0.699	226192_at	AR	androgen receptor
22	0.695	1553089_a_at	WFDC2	WAP four-disulfide core domain 2
23	0.695	1556097_at	HOMER2	homer homolog 2 (Drosophila)
24	0.694	239556_at	LOC645513	uncharacterized LOC645513
25	0.688	232944_at	THSD4	thrombospondin, type I, domain containing 4
26	0.687	241470_x_at		
27	0.684	228195_at	C2orf88	chromosome 2 open reading frame 88
28	0.684	226197_at	AR	androgen receptor
29	0.683	218614_at	C12orf35	chromosome 12 open reading frame 35
30	0.682	1561956_at	NPAS3	neuronal PAS domain protein 3
31	0.680	231817_at	USP53	ubiquitin specific peptidase 53
32	0.680	1569361_a_at	LOC100129098	uncharacterized LOC100129098
33	0.673	230083_at	USP53	ubiquitin specific peptidase 53
34	0.671	226506_at	THSD4	thrombospondin, type I, domain containing 4
35	0.671	226016_at	CD47	CD47 molecule
36	0.664	211596_s_at	LRIG1	leucine-rich repeats and immunoglobulin-like domains 1
37	0.663	1552261_at	WFDC2	WAP four-disulfide core domain 2
38	0.662	239611_at	NPAS3	neuronal PAS domain protein 3
39	0.660	212909_at	LYPD1	LY6/PLAUR domain containing 1
40	0.657	205317_s_at	SLC15A2	solute carrier family 15 (H+/peptide transporter), member 2
41	0.657	229281_at	NPAS3	neuronal PAS domain protein 3
42	0.657	219455_at	C7orf63	chromosome 7 open reading frame 63
43	0.656	227379_at	MBOAT1	membrane bound O-acyltransferase domain containing 1
44	0.655	228345_at	CHIC1	cysteine-rich hydrophobic domain 1
45	0.652	205316_at	SLC15A2	solute carrier family 15 (H+/peptide transporter), member 2
46	0.652	230412_at	NPAS3	neuronal PAS domain protein 3
47	0.644	217080_s_at	HOMER2	homer homolog 2 (Drosophila)
48	0.643	206799_at	SCGB1D2	secretoglobin, family 1D, member 2
49	0.643	229903_x_at	RNPC3	RNA-binding region (RNP1, RRM) containing 3
50	0.642	227152_at	C12orf35	chromosome 12 open reading frame 35

The probe sets were identified using GENEVESTIGATOR and ranked according to the Pearson correlation coefficient (Score).

Table S12 The top 50 Affymetrix probe sets co-regulated with *ID2* in ovarian cancer.

Rank	Score	Probe set	Gene Symbol	Description
	1.000	201565_s_at	ID2	inhibitor of DNA binding 2, dominant negative helix-loop-helix
1	0.942	201566_x_at	ID2	inhibitor of DNA binding 2, dominant negative helix-loop-helix
2	0.810	208937_s_at	ID1	inhibitor of DNA binding 1, dominant negative helix-loop-helix
3	0.801	213139_at	SNAI2	snail homolog 2 (Drosophila)
4	0.796	201162_at	IGFBP7	insulin-like growth factor binding protein 7
5	0.793	209156_s_at	COL6A2	collagen, type VI, alpha 2
6	0.792	202007_at	NID1	nidogen 1
7	0.784	233555_s_at	SULF2	sulfatase 2
8	0.770	207826_s_at	ID3	inhibitor of DNA binding 3, dominant negative helix-loop-helix
9	0.767	217892_s_at	LIMA1	LIM domain and actin binding 1
10	0.766	215719_x_at	FAS	Fas (TNF receptor superfamily, member 6)
11	0.766	212091_s_at	COL6A1	collagen, type VI, alpha 1
12	0.765	201508_at	IGFBP4	insulin-like growth factor binding protein 4
13	0.765	224724_at	SULF2	sulfatase 2
14	0.765	218613_at	PSD3	pleckstrin and Sec7 domain containing 3
15	0.758	212096_s_at	MTUS1	microtubule associated tumor suppressor 1
16	0.753	232935_at	LHFP	lipoma HMGIC fusion partner
17	0.753	212158_at	SDC2	syndecan 2
18	0.748	218309_at	CAMK2N1	calcium/calmodulin-dependent protein kinase II inhibitor 1
19	0.748	202609_at	EPS8	epidermal growth factor receptor pathway substrate 8
20	0.747	222303_at	ETS2	v-ets avian erythroblastosis virus E26 oncogene homolog 2
21	0.744	210762_s_at	DLC1	deleted in liver cancer 1
22	0.743	212099_at	RHOB	ras homolog family member B
23	0.743	213488_at	SNED1	sushi, nidogen and EGF-like domains 1
24	0.738	223287_s_at	FOXP1	forkhead box P1
25	0.735	202310_s_at	COL1A1	collagen, type I, alpha 1
26	0.735	225056_at	SIPA1L2	signal-induced proliferation-associated 1 like 2
27	0.735	201666_at	TIMP1	TIMP metallopeptidase inhibitor 1
28	0.734	225524_at	ANTXR2	anthrax toxin receptor 2
29	0.732	207173_x_at	CDH11	cadherin 11, type 2, OB-cadherin (osteoblast)
30	0.730	203243_s_at	PDLIM5	PDZ and LIM domain 5
31	0.728	201983_s_at	EGFR	epidermal growth factor receptor
32	0.727	200974_at	ACTA2	actin, alpha 2, smooth muscle, aorta
33	0.726	201944_at	HEXB	hexosaminidase B (beta polypeptide)
34	0.726	218831_s_at	FCGRT	Fc fragment of IgG, receptor, transporter, alpha
35	0.726	204114_at	NID2	nidogen 2 (osteonidogen)
36	0.725	201809_s_at	ENG	endoglin
37	0.724	218686_s_at	RHBDF1	rhomboid 5 homolog 1 (Drosophila)
38	0.720	203058_s_at	PAPSS2	3'-phosphoadenosine 5'-phosphosulfate synthase 2
39	0.720	201531_at	ZFP36	zinc finger protein 36, C3H type, homolog (mouse)
40	0.720	217763_s_at	RAB31	RAB31, member RAS oncogene family
41	0.720	209147_s_at	PPAP2A	phosphatidic acid phosphatase type 2A
42	0.719	212294_at	GNG12	guanine nucleotide binding protein (G protein), gamma 12
43	0.719	202762_at	ROCK2	Rho-associated, coiled-coil containing protein kinase 2
44	0.718	223028_s_at	SNX9	sorting nexin 9
45	0.716	223391_at	SGPP1	sphingosine-1-phosphate phosphatase 1
46	0.716	219237_s_at	DNAJB14	DnaJ (Hsp40) homolog, subfamily B, member 14
47	0.716	218086_at	NPDC1	neural proliferation, differentiation and control, 1
48	0.716	205466_s_at	HS3ST1	heparan sulfate (glucosamine) 3-O-sulfotransferase 1
49	0.715	202284_s_at	CDKN1A	cyclin-dependent kinase inhibitor 1A (p21, Cip1)
50	0.714	209101_at	CTGF	connective tissue growth factor

The probe sets were identified using GENEVESTIGATOR and ranked according to the Pearson correlation coefficient (Score).

Table S13 The top 50 Affymetrix probe sets co-regulated with *ID3* in ovarian cancer.

Rank	Score	Probe set	Gene Symbol	Description
	1	207826_s_at	ID3	inhibitor of DNA binding 3, dominant negative helix-loop-helix
1	0.8921	208937_s_at	ID1	inhibitor of DNA binding 1, dominant negative helix-loop-helix
2	0.8073	211651_s_at	LAMB1	laminin, beta 1
3	0.7842	210139_s_at	PMP22	peripheral myelin protein 22
4	0.7758	201565_s_at	ID2	inhibitor of DNA binding 2, dominant negative helix-loop-helix
5	0.7751	208782_at	FSTL1	follistatin-like 1
6	0.773	201566_x_at	ID2	inhibitor of DNA binding 2, dominant negative helix-loop-helix
7	0.772	203325_s_at	COL5A1	collagen, type V, alpha 1
8	0.7663	201505_at	LAMB1	laminin, beta 1
9	0.7591	233555_s_at	SULF2	sulfatase 2
10	0.7589	224724_at	SULF2	sulfatase 2
11	0.7537	212895_s_at	ABR	active BCR-related
12	0.7535	202007_at	NID1	nidogen 1
13	0.7435	212488_at	COL5A1	collagen, type V, alpha 1
14	0.7422	213139_at	SNAI2	snail homolog 2 (Drosophila)
15	0.7363	219179_at	DACT1	dapper, antagonist of beta-catenin, homolog 1 (Xenopus laevis)
16	0.7319	207172_s_at	CDH11	cadherin 11, type 2, OB-cadherin (osteoblast)
17	0.7293	212154_at	SDC2	syndecan 2
18	0.729	212158_at	SDC2	syndecan 2
19	0.7286	212157_at	SDC2	syndecan 2
20	0.7274	215646_s_at	VCAN	versican
21	0.7243	222449_at	PMEPA1	prostate transmembrane protein, androgen induced 1
22	0.724	207173_x_at	CDH11	cadherin 11, type 2, OB-cadherin (osteoblast)
23	0.7226	212423_at	ZCCHC24	zinc finger, CCHC domain containing 24
24	0.7199	201508_at	IGFBP4	insulin-like growth factor binding protein 4
25	0.7159	213905_x_at	BGN	biglycan
26	0.7124	211571_s_at	VCAN	versican
27	0.7108	212464_s_at	FN1	fibronectin 1
28	0.707	202766_s_at	FBN1	fibrillin 1
29	0.7068	212489_at	COL5A1	collagen, type V, alpha 1
30	0.7057	227080_at	ZNF697	zinc finger protein 697
31	0.7053	226695_at	PRRX1	paired related homeobox 1
32	0.7047	209156_s_at	COL6A2	collagen, type VI, alpha 2
33	0.7039	210495_x_at	FN1	fibronectin 1
34	0.7038	216442_x_at	FN1	fibronectin 1
35	0.7002	218613_at	PSD3	pleckstrin and Sec7 domain containing 3
36	0.6992	217892_s_at	LIMA1	LIM domain and actin binding 1
37	0.6989	203710_at	ITPR1	inositol 1,4,5-trisphosphate receptor, type 1
38	0.697	204163_at	EMILIN1	elastin microfibril interfacer 1
39	0.6966	202975_s_at	RHOBTB3	Rho-related BTB domain containing 3
40	0.6943	202765_s_at	FBN1	fibrillin 1
41	0.6932	225202_at	RHOBTB3	Rho-related BTB domain containing 3
42	0.6919	202196_s_at	DKK3	dickkopf 3 homolog (Xenopus laevis)
43	0.6914	212813_at	JAM3	junctional adhesion molecule 3
44	0.6904	227719_at	SMAD9	SMAD family member 9
45	0.6898	201792_at	AEBP1	AE binding protein 1
46	0.6897	200827_at	PLOD1	procollagen-lysine, 2-oxoglutarate 5-dioxygenase 1
47	0.6889	212099_at	RHOB	ras homolog family member B
48	0.6864	202363_at	SPOCK1	sparc/osteonectin, cwcv and kazal-like domains proteoglycan
49	0.6863	209147_s_at	PPAP2A	phosphatidic acid phosphatase type 2A
50	0.6855	210762_s_at	DLC1	deleted in liver cancer 1

The probe sets were identified using GENEVESTIGATOR and ranked according to the Pearson correlation coefficient (Score).

Table S14 The top 50 Affymetrix probe sets co-regulated with *PTPRC/CD45* in ovarian cancer.

Rank	Score	Probe set	Gene Symbol	Description
	1	207238_s_at	PTPRC	protein tyrosine phosphatase, receptor type, C
1	0.9778	212588_at	PTPRC	protein tyrosine phosphatase, receptor type, C
2	0.9775	204118_at	CD48	CD48 molecule
3	0.9769	227266_s_at	FYB	FYN binding protein
4	0.9732	205270_s_at	LCP2	lymphocyte cytosolic protein 2 (SH2 domain containing leukocyte protein of 76kDa)
5	0.9713	224356_x_at	MS4A6A	membrane-spanning 4-domains, subfamily A, member 6A
6	0.9701	223922_x_at	MS4A6A	membrane-spanning 4-domains, subfamily A, member 6A
7	0.9677	210895_s_at	CD86	CD86 molecule
8	0.9672	220330_s_at	SAMSN1	SAM domain, SH3 domain and nuclear localization signals 1
9	0.9669	223280_x_at	MS4A6A	membrane-spanning 4-domains, subfamily A, member 6A
10	0.9662	205269_at	LCP2	lymphocyte cytosolic protein 2 (SH2 domain containing leukocyte protein of 76kDa)
11	0.9657	203416_at	CD53	CD53 molecule
12	0.9656	211742_s_at	EVI2B	ecotropic viral integration site 2B
13	0.9654	226841_at	MPEG1	macrophage expressed 1
14	0.9646	205831_at	CD2	CD2 molecule
15	0.9638	213193_x_at	TRBC1	T cell receptor beta constant 1
16	0.9631	230391_at	CD84	CD84 molecule
17	0.963	205488_at	GZMA	granzyme A (granzyme 1, cytotoxic T-lymphocyte-associated serine esterase 3)
18	0.9629	203471_s_at	PLEK	pleckstrin
19	0.9623	1405_i_at	CCL5	chemokine (C-C motif) ligand 5
20	0.9621	210915_x_at	TRBC1	T cell receptor beta constant 1
21	0.962	226818_at	MPEG1	macrophage expressed 1
22	0.962	1555759_a_at	CCL5	chemokine (C-C motif) ligand 5
23	0.9609	204959_at	MNDA	myeloid cell nuclear differentiation antigen
24	0.9605	209901_x_at	AIF1	allograft inflammatory factor 1
25	0.958	204923_at	SASH3	SAM and SH3 domain containing 3
26	0.9576	1555638_a_at	SAMSN1	SAM domain, SH3 domain and nuclear localization signals 1
27	0.9571	219243_at	GIMAP4	GTPase, IMAP family member 4
28	0.9571	210644_s_at	LAIR1	leukocyte-associated immunoglobulin-like receptor 1
29	0.9566	213095_x_at	AIF1	allograft inflammatory factor 1
30	0.9555	210972_x_at	YME1L1, TRAV20	YME1-like 1 (S. cerevisiae),T cell receptor alpha variable 20,T cell receptor alpha joining 17,T cell receptor alpha constant
31	0.9554	204774_at	EVI2A	ecotropic viral integration site 2A
32	0.9551	206715_at	TFEC	transcription factor EC
33	0.9547	211796_s_at	TRBC2, TRBC1IL23A	T cell receptor beta constant 2,T cell receptor beta constant 1,interleukin 23, alpha subunit p19
34	0.9534	204655_at	CCL5	chemokine (C-C motif) ligand 5
35	0.9531	1554899_s_at	FCER1G	Fc fragment of IgE, high affinity I, receptor for; gamma polypeptide
36	0.9526	205159_at	CSF2RB	colony stimulating factor 2 receptor, beta, low -affinity (granulocyte-macrophage)
37	0.952	230422_at	FPR3	formyl peptide receptor 3
38	0.9509	204912_at	IL10RA	interleukin 10 receptor, alpha
39	0.9504	219666_at	MS4A6A	membrane-spanning 4-domains, subfamily A, member 6A
40	0.9503	206991_s_at	CCR5	chemokine (C-C motif) receptor 5 (gene/pseudogene)
41	0.9494	209906_at	C3AR1	complement component 3a receptor 1
42	0.9489	214574_x_at	LST1	leukocyte specific transcript 1
43	0.9475	213160_at	DOCK2	dedicator of cytokinesis 2
44	0.9475	203761_at	SLA	Src-like-adaptor
45	0.9463	203922_s_at	CYBB	cytochrome b-245, beta polypeptide
46	0.9463	211795_s_at	FYB	FYN binding protein
47	0.9457	208018_s_at	HCK	hemopoietic cell kinase
48	0.9457	229560_at	TLR8	toll-like receptor 8
49	0.9446	215051_x_at	AIF1	allograft inflammatory factor 1
50	0.9436	213566_at	RNASE6	ribonuclease, RNase A family, k6

The probe sets were identified using GENEVESTIGATOR and ranked according to the Pearson correlation coefficient (Score).

Table S15 The 10-top-AID/APOBEC signature-linked Canonical Pathways identified by systems biology approach (algorithm II).

Canonical output_mixed_and_3_individual [A]									Related studies [B]	
Mixed				APOBEC3G	ESR1	ID2	ID3	PTPRC (CD45)		
Pos.	Canonical pathways	P-value	Molecules	Molecules	Molecules	Molecules	Molecules	Molecules	Ovarian Cancer	Others
1	Hepatic Fibrosis / Hepatic Stellate Cell Activation	8.32E-08	COL1A1, IGFBP4, CCR5, FN1, CTGF, TIMP1, ACTA2, IL10RA, CCL5, FAS, EGFR			COL1A1, IGFBP4, CTGF, TIMP1, ACTA2, FAS, EGFR	IGFBP4, FN1	CCR5, IL10RA, CCL5	Batista et al. 2013<br>Mateescu et al. 2011<br>Samrao et al. 2012	Hatzirodos et al. 2014
2	Crosstalk between Dendritic Cells and Natural Killer Cells	2.09E-04	CSF2RB, ACTA2, IL15, CD86, TLR3, FAS	IL15, TLR3		ACTA2, FAS		CSF2RB, CD86		Wong et al. 2013<br>Harizi et al. 2013

Canonical 10-top-output_mixed_and_2_individual [A]									Related studies [B]	
Mixed				APOBEC3G	ESR1	ID2	ID3	PTPRC (CD45)		
Pos.	Canonical pathways	P-value	Molecules	Molecules	Molecules	Molecules	Molecules	Molecules	Ovarian Cancer	Others
1	Altered T Cell and B Cell Signaling in Rheumatoid Arthritis	1.51E-06	HLA-DOA, IL15, TLR8, FCER1G, CD86, TLR3, TNFSF13B, FAS	HLA-DOA, IL15, TLR3, TNFSF13B				TLR8, FCER1G, CD86		Choy et al. 2012
2	Communication between Innate and Adaptive Immune Cells	2.45E-05	IL15, TLR8, FCER1G, CD86, CCL5, TLR3, TNFSF13B	IL15, CCL5, TLR3, TNFSF13B				TLR8, FCER1G, CD86, CCL5	Scarlett et al. 2009	
3	Role of Pattern Recognition Receptors in Recognition of Bacteria and Viruses	3.47E-05	IFIH1, CLEC7A, TLR8, CASP1, CCL5, TLR3, C3AR1	IFIH1, CLEC7A, CASP1, CCL5, TLR3				TLR8, CCL5, C3AR1		Zhou et al. 2009<br>Peng et al. 2006<br>Casartelli et al. 2010
4	Allograft Rejection Signaling	1.35E-04	HLA-DOA, FCER1G, CD86, HLA-DPB1, HLA-DPA1, FAS	HLA-DOA, HLA-DPB1, HLA-DPA1						Wasiuk et al. 2012
5	CCR5 Signaling in Macrophages	5.25E-04	CCR5, FCER1G, CCL5, GNG12, FAS			GNG12, FAS		CCR5, FCER1G, CCL5	Milliken et al. 2002<br>Tsukishiro et al. 2006	Lee et al. 2003
6	RhoGDI Signaling	1.29E-03	ROCK2, RHOB, ACTA2, DLC1, ESR1, CDH11, GNG12			ROCK2, RHOB, ACTA2, DLC1, CDH11, GNG12	RHOB, DLC1, CDH11			Harding et al. 2010
7	TREM1 Signaling	2.34E-03	TLR8, CASP1, CD86, TLR3	CASP1, TLR3				TLR8, CD86		Ford et al. 2009
8	Pathogenesis of Multiple Sclerosis	3.09E-03	CCR5, CCL5	CCL5				CCR5, CCL5		
9	Type I Diabetes Mellitus Signaling	3.63E-03	HLA-DOA, FCER1G, CD86, IRF1, FAS	HLA-DOA, IRF1				FCER1G, CD86	Lee et al. 2013	
10	Tec Kinase Signaling	3.72E-03	RHOB, ACTA2, HCK, FCER1G, GNG12, FAS			RHOB, ACTA2, GNG12, FAS		FCER1G, CD86		Potter et al. 2014

[A] IPA nomenclature was used for canonical pathways. The 10-top-output_mixed_and_3/2 individual results are shown; the ranking is based on the corresponding IPA-based p-value of the mixed_output results; the molecules mapped to the pathway are listed. In respect of canonical pathways, an overlap between output_mixed and 4 or all 5 output_individual was not observed, thus, results of "mixed_and_3 individual" are shown. Since analysis for the 10-top-output_mixed_and_3 individual revealed only two Canonical Pathways, the results of the 10-top-output_mixed_and_2 individual are additionally shown.

[B] Literature search-based results annotating those canonical pathways in ovarian cancer or other related studies.

Table S16 The top-AID/APOBEC signature-linked Upstream Regulators identified by systems biology approach (algorithm II; overlap with 4 individual target genes).

Upstream Regulators-top-output_mixed_and_4_individual								
Pos.	Upstream regulators	Mixed		APOBEC3G	ESR1	ID2	ID3	PTPRC (CD45)
		P-value	Molecules	Molecules	Molecules	Molecules	Molecules	Molecules
1	TNF	1.25E-16	ABR, AEBP1, ALOX5, AR, BGN, CARD16, CARD6, CASP1, CCL5, CCR5, CD47, CD86, CDH11, CDKN1A, CIITA, COL1A1, CSF2RB, CTGF, CTSS, CYBB, EGFR, ENG, ESR1, FAS, FCER1G, FCGRT, FN1, GBP2, HEXB, ID1, ID3, IFIH1, IGFBP4, IL10RA, IL15, IRF1, ITGAM, ITPR1, NID1, PPAP2A, PSMB10, PSMB9, RARRES3, SDC2, TIMP1, TLR3, T	CASP1, CCL5, CTSS, GBP2, IL15, IRF1, ITGAM, PSMB10, PSMB9, RARRES3, TLR3, TNFSF13B		CDH11, CDKN1A, EGFR, FAS, HEXB, NID1, PPAP2A, TIMP1	AEBP1, CDH11, FN1, ITPR1, NID1, PPAP2A	CCL5, CCR5, CD86, CYBB
2	APOE	3.80E-10	ACTA2, BGN, CCL5, CCR5, CD86, COL1A1, CTGF, CTSS, CYBB, EMILIN1, IL10RA, ITGAM, PMEPA1, TIMP1, TNFRSF14	CCL5, CTSS, ITGAM, TNFRSF14		ACTA2, COL1A1, CTGF, TIMP1	BGN, EMILIN1, PMEPA1	CCL5, CCR5, CD86, CYBB, IL10RA
3	CD44	1.59E-07	BGN, CCL5, CCR5, CDKN1A, CLEC7A, COL1A1, EMILIN1, FAS, FN1, PMEPA1, TLR8	CCL5, CLEC7A		COL1A1, FAS	BGN, EMILIN1, PMEPA1	CCL5, CCR5, TLR8
4	TICAM1	2.21E-05	CCL5, CD86, IL15, IRF1, PPAP2A, RHOB, TFEC, TLR3	CCL5, IL15, IRF1, TLR3		PPAP2A, RHOB	PPAP2A, RHOB	CCL5, CD86, TFEC
5	Ifi204 (includes others)	3.16E-03	CCL5, ID2	CCL5		ID2	ID2	CCL5

In respect of upstream regulators, an overlap between output_mixed and all 5 output_individual was not observed, thus, the top-output_mixed_and_4 individual are shown; the ranking is based on the corresponding IPA-based p-value of the output_mixed; the molecules associated with the upstream regulator are listed.

Table S17 The 10-top-AID/APOBEC signature-linked Upstream Regulators identified by systems biology approach (algorithm II; overlap with 3 individual target genes).

Upstream Regulators-10-top-output_mixed_and_3_individual								
Pos.	Upstream Regulator	Mixed		APOBEC3G	ESR1	ID2	ID3	PTPRC (CD45)
		P-value	Molecules	Molecules	Molecules	Molecules	Molecules	Molecules
1	Interferon alpha	3.48E-19	APOBEC3F, APOBEC3G, APOL3/APOL4, C3AR1, CASP1, CCL5, CCR5, CD86, CDKN1A, CIITA, CSF2RB, EGFR, FAS, GBP2, IFIH1, IGFBP4, IL10RA, IL15, IRF1, ITGAM, MNDA, PSMB9, RARRES3, TLR3, TLR8, TNFSF13B, TRIM22	APOBEC3F, APOBEC3G, APOL3/APOL4, CASP1, CIITA, IL15, IRF1, TLR3, TNFSF13B		CDKN1A, IGFBP4		CSF2RB, TLR8
2	TGFB1	8.38E-18	ACTA2, ALOX5, BGN, CASP1, CCL5, CCR5, CD86, CDH11, CDKN1A, CIITA, COL1A1, COL5A1, COL6A1, COL6A2, CTGF, CTSS, CYBB, DKK3, DOCK2, EMILIN1, ENG, FAS, FBN1, FCER1G, FN1, GMPR, GZMA, HEXB, ID1, ID2, ID3, IFIH1, IGFBP4, IGFBP7, IL10RA, IL15, IRF1, ITGAM, ITPR1, PDLIM5, PLOD1, PMEPA1, PTPRC, RAB31, RHOB,	ALOX5, CCL5, IRF1, ITGAM		ACTA2, CDH11, CDKN1A, COL1A1, CTGF, ENG, FAS, HEXB, ID1, RAB31, RHOB, SNAI2, TIMP1, ZFP36	BGN, CDH11, COL5A1, FBN1, FN1, ID1, PMEPA1, RHOB, SNAI2, SPOCK1	
3	IL10	1.91E-15	ACTA2, CCL5, CCR5, CD2, CD86, CDKN1A, CIITA, CLEC7A, COL1A1, CSF2RB, CTSS, FAS, FCER1G, GZMA, IGFBP4, IL10RA, IRF1, PSMB9, TIMP1, TLR3, TLR8, TNFSF13B, VCAN, ZFP36	CCL5, CIITA, TLR3		ACTA2, CDKN1A, TIMP1, ZFP36		CCL5, CD2, CD86
4	STAT3	5.98E-15	CASP1, CCL5, CCR5, CD86, CDKN1A, CIITA, COL5A1, ESR1, FAS, FCER1G, FN1, GBP2, ID2, IFIH1, IRF1, ITGAM, PSMB9, SMAD9, TIMP1, TLR3, TRIM14, TRIM22, VCAN, XAF1, ZFP36	CASP1, CIITA, GBP2, IRF1, ITGAM, PSMB9, TLR3, XAF1		CDKN1A, FCGRT	FN1, SMAD9, VCAN	
5	IL13	7.28E-14	C3AR1, CASP1, CCL5, CCR5, CD48, CD86, CLEC7A, COL1A1, COL6A2, CTGF, CTSS, CYBB, EGFR, FAS, FGD2, HOMER2, NID1, SAMSN1, SLA, SNAI2, TFEC, TIMP1, TNFSF13B	CASP1, CCL5, CTSS, FGD2		FAS, NID1, TIMP1		C3AR1, CCL5, CCR5, CD48, CD86, CYBB, SAMSN1, SLA
6	Alpha catenin	1.43E-11	BGN, CDH11, COL1A1, COL5A1, COL6A1, COL6A2, ENG, FSTL1, IGFBP4, IGFBP7, IRF1, ITGAM, TIMP1	IRF1, ITGAM		CDH11, COL1A1, COL6A1, COL6A2, ENG, IGFBP4, IGFBP7, TIMP1	BGN, CDH11, COL5A1, COL6A2, FSTL1, IGFBP4	
7	IFNB1	6.05E-11	CARD6, CASP1, CCL5, CCR5, CD86, CDKN1A, DKK3, FBN1, GBP2, IFIH1, IRF1, LAMB1, NID2, RARRES3, TIMP1, TLR3, XAF1	CASP1, CCL5, IFIH1, IRF1, TLR3			DKK3, FBN1, LAMB1	CCL5, CD86
8	TGFBR1	9.96E-09	ACTA2, BGN, CD86, CDKN1A, COL1A1, CTGF, FN1, ZFP36			ACTA2, CTGF	FN1	CD86
9	SPI1	1.51E-08	CDKN1A, CIITA, CSF2RB, CTSS, CYBB, FCER1G, FCGRT, ID2, ITGAM, PTPRC, TFEC, TNFRSF14	CIITA, CTSS, ITGAM, TNFRSF14		CDKN1A, FCGRT		CSF2RB, CYBB, PTPRC
10	CD40	2.6E-08	APOBEC3G, CCL5, CD86, CDKN1A, FAS, IL10RA, IL15, IRF1, ITGAM, PSMB10, PSMB9, SAMSN1, TNFSF13B	APOBEC3G, CCL5, IRF1, PSMB10, PSMB9, TNFSF13B		CDKN1A, FAS		CCL5, CD86, SAMSN1

Since analysis for the 10-top-output_mixed_and_4 individual revealed only five Upstream Regulators, the results of the 10-topoutput_ mixed_and_3 individual are additionally shown. The ranking is based on the corresponding IPA-based p-value of the output_mixed; the molecules associated with the upstream regulator are listed.

References of Supplemental Material

Batista L, Gruosso T, Mechta-Grigoriou F. 2013. Ovarian cancer emerging subtypes: role of oxidative stress and fibrosis in tumour development and response to treatment. *The international journal of biochemistry & cell biology* 45(6): 1092-1098.

Casartelli N, Guivel-Benhassine F, Bouziat R, Brandler S, Schwartz O, Moris A. 2010. The antiviral factor APOBEC3G improves CTL recognition of cultured HIV-infected T cells. *The Journal of experimental medicine* 207(1): 39-49.

Choy E. 2012. Understanding the dynamics: pathways involved in the pathogenesis of rheumatoid arthritis. *Rheumatology (Oxford)* 51 Suppl 5: v3-11.

Domcke S, Sinha R, Levine DA, Sander C, Schultz N. 2013. Evaluating cell lines as tumour models by comparison of genomic profiles. *Nat Commun* 4:2126.

Ford JW, McVicar DW. 2009. TREM and TREM-like receptors in inflammation and disease. *Current opinion in immunology* 21(1): 38-46.

Harding MA, Theodorescu D. 2010. RhoGDI signaling provides targets for cancer therapy. *Eur J Cancer* 46(7): 1252-1259.

Harizi H. 2013. Reciprocal crosstalk between dendritic cells and natural killer cells under the effects of PGE2 in immunity and immunopathology. *Cellular & molecular immunology* 10(3): 213-221.

Hatzirodos N, Hummitzsch K, Irving-Rodgers HF, Harland ML, Morris SE, Rodgers RJ. 2014. Transcriptome profiling of granulosa cells from bovine ovarian follicles during atresia. *BMC genomics* 15: 40.

Kim EY, Bhattacharya T, Kunstman K, Swantek P, Koning FA, Malim MH, Wolinsky SM. 2010. Human APOBEC3G-mediated editing can promote HIV-1 sequence diversification and accelerate adaptation to selective pressure. *Journal of virology* 84(19): 10402-10405.

Lee C, Liu QH, Tomkowicz B, Yi Y, Freedman BD, Collman RG. 2003. Macrophage activation through CCR5- and CXCR4-mediated gp120-elicited signaling pathways. *Journal of leukocyte biology* 74(5): 676-682.

Lee JY, Jeon I, Kim JW, Song YS, Yoon JM, Park SM. 2013. Diabetes mellitus and ovarian cancer risk: a systematic review and meta-analysis of observational studies. *International journal of gynecological cancer : official journal of the International Gynecological Cancer Society* 23(3): 402-412.

Leonard B, Hart SN, Burns MB, Carpenter MA, Temiz NA, Rathore A, Vogel RI, Nikas JB, Law EK, Brown WL et al. 2013. APOBEC3B upregulation and genomic mutation patterns in serous ovarian carcinoma. *Cancer research* 73(24): 7222-7231.

Mateescu B, Batista L, Cardon M, Gruosso T, de Feraudy Y, Mariani O, Nicolas A, Meyniel JP, Cottu P, Sastre-Garau X et al. 2011. miR-141 and miR-200a act on ovarian tumorigenesis by controlling oxidative stress response. *Nature medicine* 17(12): 1627-1635.

Milliken D, Scotton C, Raju S, Balkwill F, Wilson J. 2002. Analysis of chemokines and chemokine receptor expression in ovarian cancer ascites. *Clinical cancer research : an official journal of the American Association for Cancer Research* 8(4): 1108-1114.

Peng G, Lei KJ, Jin W, Greenwell-Wild T, Wahl SM. 2006. Induction of APOBEC3 family proteins, a defensive maneuver underlying interferon-induced anti-HIV-1 activity. *The Journal of experimental medicine* 203(1): 41-46.

Potter DS, Kelly P, Denneny O, Juvin V, Stephens LR, Dive C, Morrow CJ. 2014. BMX acts downstream of PI3K to promote colorectal cancer cell survival and pathway inhibition sensitizes to the BH3 mimetic ABT-737. *Neoplasia* 16(2): 147-157.

Samrao D, Wang D, Ough F, Lin YG, Liu S, Menesses T, Yessaian A, Turner N, Pejovic T, Mhawech-Fauceglia P. 2012. Histologic parameters predictive of disease outcome in women with advanced stage ovarian carcinoma treated with neoadjuvant chemotherapy. *Translational oncology* 5(6): 469-474.

Scarlett UK, Cubillos-Ruiz JR, Nesbeth YC, Martinez DG, Engle X, Gewirtz AT, Ahonen CL, Conejo-Garcia JR. 2009. In situ stimulation of CD40 and Toll-like receptor 3 transforms ovarian

cancer-infiltrating dendritic cells from immunosuppressive to immunostimulatory cells. *Cancer research* 69(18): 7329-7337.

Tsukishiro S, Suzumori N, Nishikawa H, Arakawa A, Suzumori K. 2006. Elevated serum RANTES levels in patients with ovarian cancer correlate with the extent of the disorder. *Gynecologic oncology* 102(3): 542-545.

Wasiuk A, Dalton DK, Schpero WL, Stan RV, Conejo-Garcia JR, Noelle RJ. 2012. Mast cells impair the development of protective anti-tumor immunity. *Cancer immunology, immunotherapy : CII* 61(12): 2273-2282.

Wong JL, Berk E, Edwards RP, Kalinski P. 2013. IL-18-primed helper NK cells collaborate with dendritic cells to promote recruitment of effector CD8+ T cells to the tumor microenvironment. *Cancer research* 73(15): 4653-4662.

Zhou L, Wang X, Wang YJ, Zhou Y, Hu S, Ye L, Hou W, Li H, Ho WZ. 2009. Activation of toll-like receptor-3 induces interferon-lambda expression in human neuronal cells. *Neuroscience* 159(2): 629-637.
